# Supplementary material for: Targeting undruggable carbohydrate recognition sites through focused fragment library design
Source: Commun Chem. 2022 May 20;5:64. doi: 10.1038/s42004-022-00679-3 (PMC9814205; doi:10.1038/s42004-022-00679-3)
Supplement: Supplementary file 1 — Supplementary Information [file 42004_2022_679_MOESM1_ESM.pdf]

Supporting Information

Targeting undruggable carbohydrate recognition sites through focused fragment library design

Elena Shanina[1,2], Sakonwan Kuhaudomlarp[3,4,5], Eike Siebs[6,7,8], Felix F. Fuchsberger[1,2,9,10], Maxime Denis[9,10], Priscila da Silva Figueiredo Celestino Gomes[11,12], Mads H. Clausen[13], Peter H. Seeberger[1,2], Didier Rognan[11], Alexander Titz[6,7,8], Anne Imberty[3] & Christoph Rademacher[1,2,9,10*]

[1] Max Planck Institute of Colloids and Interfaces, Department of Biomolecular Systems, Am Mühlenberg 1, 14424 Potsdam, Germany;
[2] Freie Universität Berlin, Department of Chemistry and Biochemistry, Arnimallee 22, 14195 Berlin, Germany;
[3] University Grenoble Alpes, CNRS, CERMAV, Grenoble, France;
[4] Department of Biochemistry, Faculty of Science, Mahidol University, 10400 Bangkok, Thailand;
[5] Center for Excellence in Protein and Enzyme Technology, Faculty of Science, Mahidol University, 10400 Bangkok, Thailand;
[6] Chemical Biology of Carbohydrates (CBCH), Helmholtz Institute for Pharmaceutical Research Saarland (HIPS), Helmholtz Centre for Infection Research, 66123 Saarbrücken, Germany;
[7] Saarland University, Department of Chemistry, 66123 Saarbrücken, Germany;
[8] German Center for Infection Research (DZIF), Hannover-Braunschweig, Germany;
[9] University of Vienna, Department of Pharmaceutical Sciences, Althanstrasse 14, 1090 Vienna, Austria;
email: christoph.rademacher@univie.ac.at
[10] University of Vienna, Department of Microbiology, Immunology and Genetics, Max F. Berutz Labs, Biocenter 5, 1030 Vienna, Austria;
[11] Laboratoire d'Innovation Thérapeutique, UMR 7200 CNRS-Université de Strasbourg, 67400 Illkirch, France;
[12] Department of Physics, College of Sciences and Mathematics, Auburn University, 36849 Auburn, USA;
[13] Technical University of Denmark, Center for Nanomedicine and Theranostics, Department of Chemistry, Kemitorvet 207, 2800 Kongens Lyngby, Denmark.

Table of Contents

Supplementary Results and Discussion 3

Virtual screening of LecA hits 3
Virtual screening of LecB hits 4
Evaluation of virtual screening hits for LecA 4
Evaluation of virtual screening hits for LecB 4
Druggability assessment of Ca^{2+}-dependent lectins 5
*Chemical derivatization of hydroxamate **1*** 5
TROSY NMR study of hydroxamate derivatives 6
SPR analysis of hydroxamate derivatives 6
Competitive fluorescence polarization assay and PrOF NMR of LecA with hydroxamate derivatives 7
*Competitive ^{19}F T_2-filtered NMR with hydroxamate derivative **5*** 8
*Docking study of malonate **58** with metal-dependent lectins* 8
PA-IL (LecA) 8
PA-IIL (LecB) 9
DC-SIGN CRD (CD209) 9
*Competitive ^{19}F T_2-filtered NMR with malonate derivative **61*** 9
SAR study of malonate derivatives with LecB 10
SAR study of malonate derivatives with DC-SIGN 10

Supplementary Materials and Methods 11

Virtual screening of drug-like molecules targeting LecA 11
Virtual screening of drug-like molecules targeting LecB 11
Crystallographic study 12
Chemical synthesis of hydroxamate derivatives 12

Supplementary Figures 18

Figure S1 *Virtual screening of fragment and drug-like libraries for LecA.* 19
Figure S2 *MBP-like fragments identified for LecA.* 19
Figure S3 *MBP-like fragments identified for LecB.* 20
Figure S4 *Ranking hydroxamate derivatives in TROSY NMR.* 22
Figure S5 *SPR analysis of hydroxamate derivatives.* 23
Figure S6 *Investigation of the interactions between **35** and DC-SIGN or Langerin.* 24
Figure S7 *Competitive ^{19}F T_2-filtered NMR study using hydroxamate derivative **5** as a reporter.* 25
Figure S8 *PrOF NMR of hydroxamate derivatives.* 26
Figure S9 *Crystal structure of LecA in complex with **35**.* 27
Figure S10 *LecA interacts with **58** malonate derivatives.* 28
Figure S11 *Docking study of LecB with **58**.* 29
Figure S12 *Interaction map of **58** with DC-SIGN CRD.* 30
Figure S13 *^{1}H-^{15}N HSQC/TROSY NMR: **58** binding is Ca^{2+}-dependent.* 31
Figure S14 *TROSY NMR of ^{15}N LecB with **58** analogs.* 32
Figure S15 *Titration TROSY NMR studies of **58** derivatives and LecB.* 33
Figure S16 *Interaction study of DC-SIGN CRD with malonates.* 33
Figure S17 *^{1}H-^{15}N HSQC NMR titration study for malonate derivatives with ^{15}N DC-SIGN CRD.* 34

Supplementary Tables 34

Table S1 *List of LecA PDB structures and co-crystalized ligands analyzed for virtual screening.* 34
Table S2 *List of LecB PDB structures and co-crystalized ligands analyzed for virtual screening.* 35
Table S3 *Commercial and synthesized hydroxamates.* 36
Table S4 *Quantitative analysis of PrOF NMR.* 39
Table S5 *Statistics for data collection and refinement of LecA-**35** complex.* 39
Table S6 *Commercial derivatives of malonic acid **58**.* 40
Table S7 *List of assigned resonances in ^{15}N DC-SIGN CRD.* 40
Table S8 *List of resonance IDs in ^{15}N LecB.* 43
Table S9 *List of resonances in ^{15}N LecA.* 45

Supplementary Schemes 47

Supplementary Note 1 49

NMR Spectra 49

References 62

Supplementary Results and Discussion

Virtual screening of LecA hits

To predict the druggability of the carbohydrate-binding site of a Ca^{2+}-dependent lectin LecA, we used the Bioinfo Database (http://bioinfo-pharma.u-strasbg.fr/bioinfo/), a curated database of commercially available drug-like compounds, filtered for molecules with at least two potential hydrogen bond donors and acceptors to match the polar nature of the carbohydrates. Approximately 3 million molecules were docked into the crystallographic structure of LecA. First, we carefully inspected all available crystallographic LecA structures (**Table S1**) complexed with small molecules to see if there was a considerable side-chain variation that could impact the virtual screening process. As result, the carbohydrate-binding site of LecA showed to be conserved through all structures. The key aspect of post-processing of docking poses was based on the interaction similarity pattern between the docked compounds and the available ligands co-crystallized with LecA. The similarity was calculated using GRIM[1], a knowledge-based approach to convert protein-ligand complexes in interaction pattern graphs and score docking solutions by similarity of predicted interaction patterns to that already visited in lectin-carbohydrate complexes from the PDB. The similarity was quantified using the GRIM score (GrSc). Since most of the co-crystallized ligands were carbohydrate-like, we expected to have drug-like hits with similar interactions with the carbohydrate-binding site residues. Other additional filters were used to ensure the elimination of the carbohydrate-like molecules, but still maintaining the correct interaction pattern, such as the presence of interaction with the Ca^{2+} ion. In addition, the molecules with the tetrahydrofuran or tetrahydropyran scaffolds and number of polar interactions with binding site residues superior to 3 were discarded. The remaining ligands were clustered by the maximum common substructure (MCS) to select a chemically diverse

set of compounds and after visual inspection for the presence of key hydrogen bonds shared by galactose and the binding site residues as shown on example of the compound **1c** (**Figure S1a**), a total of 46 hits were selected as hits and 37 were purchased for testing.

Virtual screening of LecB hits

A similar protocol was followed for the virtual screening of LecB. The available crystal structures were analyzed (**Table S2**), but no significant variation was found for the fucose binding site residues. We screened the same database in our virtual screening protocol, the commercially available drug-like compounds from Bioinfo and the same filtering was applied for LecB. Post-processing of docking poses followed the same principle applied for LecA, which mainly focused on comparing the interaction patterns between the docked compounds and the available co-crystallized ligands with LecB using GRIM. Pose filtering followed the same criteria described for LecA and an additional filter was added for LecB, in which shape similarity with fucose was considered to improve the hit number. After selecting a diverse set through clustering and visually inspecting the molecules for the presence of key hydrogen bonds, a total of 42 molecules were selected as hits.

Evaluation of virtual screening hits for LecA

To evaluate the 37 commercial fragments identified in virtual screening (VS), we performed experimental analysis using SPR and ^{1}H-^{15}N TROSY NMR (hereafter, TROSY NMR). First, we tested compound binding in SPR, where 19 compounds demonstrated a dose-dependent response. However, compounds perturbed the resonances in ^{15}N-labeled LecA in a similar manner to methyl α-D-galactoside (hereafter, MeGal) in TROSY NMR only slightly resulting in 8 compounds (**Figures S1b-c**). Altogether, we identified two hits **1g-h**, which were confirmed in both SPR and TROSY NMR (**Figure S1e**), whereas the compound **1c** demonstrating the strongest effect in TROSY NMR was not confirmed in SPR (**Figures S1d** and **S1f**). Therefore, we performed a biochemical study based on fluorescence polarization, which did not confirm competitive properties of VS hits (*data not shown*). This suggests that hits observed in TROSY NMR and SPR were very weak ligands of LecA. Cumulatively, virtual screening identified 37 compounds that could potentially bind to the carbohydrate-binding site of LecA, but the experimental validation did not result in hits for future fragment evaluation studies.

Evaluation of virtual screening hits for LecB

In virtual screening against LecB, we identified 42 commercially available fragments. To validate these compounds, we performed TROSY NMR and FP assay. Due to the low hit rates for LecB, we combined fragments in mixtures of 10 compounds and validated its binding to LecB in TROSY NMR. The changes in spectra in the presence of compounds were compared to the positive control methyl α-L-fucoside (hereafter, MeFuc) delivering no hits for the carbohydrate-binding site of LecB (*not shown*). Finally, the analysis of compounds in a fluorescence polarization assay did not confirm hits from virtual screening (*not shown*). Taken together, virtual screening identified 42 compounds that

could potentially bind to the carbohydrate-binding site of LecB, but these were not confirmed experimentally.

Druggability assessment of Ca^{2+}-dependent lectins

To further explore the druggability of the Ca^{2+}-dependent lectins experimentally, we employed a ^{19}F and TROSY NMR screening of fragment mixtures. Here, we employed three fragment libraries and compared their efficiencies in targeting Ca^{2+}-dependent lectins. In ^{19}F NMR, all libraries were screened in presence of 5 mM EDTA to determine fragments targeting the Ca^{2+}-bearing orthosteric site of lectins, which was achieved by the addition of 10 mM $CaCl_2$ during the next screening round. First, the 3F library of 115 fluorinated, natural-product-like and Fsp^3-rich fragments with diverse shapes was screened against LecA, LecB, Langerin and BambL using ^{19}F and T_2-filtered NMR.[2] No fragments from the 3F library bound to lectins, whereas a moderate hit rate (9%) was reported for DC-SIGN previously.[2] Thus, 3F library was suited for targeting DC-SIGN. Given that hit rates from fragment screening for druggable targets are between 5–15%,[3] screening of the diversity-oriented (general) fragment library[4] delivered moderate-to-high druggability scores of Langerin (15.7%), DC-SIGN (13.5%) and BambL (48%).[4-5] Therefore, we screened the general library consisting of 350 fluorinated fragments against LecA and LecB using ^{19}F and T_2-filtered NMR, whereas 650 non-fluorinated fragments were screened against LecA additionally in TROSY NMR. Interestingly, we observed similar hit rates for LecA (15%) and LecB (14%) with many fragments containing a metal-coordinating scaffold as shown on examples of LecA and LecB with compounds **1i-r** and **1w-x**, respectively (**Figures S2a** and **S3a**). Moreover, the ^{19}F resonances of these compounds were perturbed in ^{19}F NMR in presence of 10 mM $CaCl_2$ confirming its metal chelating properties (**Figure S3b**). Next, we evaluated hits for LecA and LecB in TROSY NMR. Out of 24 fragments, **1w** was the most potent scaffold for targeting LecB as it perturbed resonances similarly to the positive control methyl α-L-fucoside (MeFuc, **Figures S3c-d**). For LecA, two non- (**1** and **1q**) and two fluorinated (**1m-n**) hits were confirmed, whereas hydroxamate **1** perturbed 40% of residues in ^{15}N LecA similar to the positive control methyl α-D-galactoside (MeGal), suggesting **1** targeted the carbohydrate-binding site (**Figures S2b-d**). Moreover, a dose-dependent binding of **1** to LecA was confirmed in SPR (**Figure S2e**). Interestingly, we observed that **1** bound to LecA, but not LecB, demonstrating an early target selectivity of the compound (**Figure S3d**).

Altogether, fragment screening demonstrated the druggability of Ca^{2+}-dependent lectins despite the limitations of *in silico* approaches. Moreover, our work highlighted the importance of chemical fragment diversity in fragment screening and indicated that metal-coordinating fragments were the most potent drug-like molecules for targeting the Ca^{2+}-dependent lectins.

*Chemical derivatization of hydroxamate **1***

The expansion of the hydroxamic acid library was on the core structure of the hydroxamate **1**. The initial structure activity relationship (SAR) study was guided by NMR (**Table S3**, *Groups 1* and *2*), which was extended after receiving the first co-crystal

structure of the hydroxamic acid **35** with LecA (**Table S3**, *Group 3*). First, the hydroxamic acid functional group was methylated either on the oxygen or the nitrogen atoms, starting from 2-phenylacetyl chloride **S2** and performing an amidation with methoxyamine or *N*-methylhydroxylamine, respectively, resulting in compounds **7** and **8** (**Scheme 1A**). Removing the hydroxy or the amine group of **1** led to commercial compounds **3** and **7** (**Scheme 2**). Next, we modified the linker between the hydroxamic acid moiety and the phenyl ring by either removing the ring (**18**) or varying its length (**35**, **43** and **44**). The synthesis was performed with hydroxylamine and the corresponding acyl chlorides after synthesis from their acids using oxalyl chloride (**Scheme 1B**). Additionally, the phenyl ring was replaced with a cyclohexyl ring in the compound **6**. To cover a broad spectrum of hydroxamic acids, compounds with a substituted phenyl ring (**9**, **10**, **15** and **11**), cyclic linkers such as a thiazole (**27**), furane (**34**), and 1-hydroxypyridin-2(1*H*)-ones were purchased. Later, derivatives of **35** were synthesized using the corresponding acid and oxalyl chloride, followed by the reaction with hydroxylamine. Linker and hydroxamic acid functional group were kept unchanged and electron donating/ withdrawing substituents (**36**, **40**, **41** and **39**) were introduced on the phenyl ring. The ring was replaced by a thiophen residue (**37**) and compound **42** containing a double bond as a spacer was designed to increase rigidity.

TROSY NMR study of hydroxamate derivatives

The protein-observed 2D NMR experiments are valuable methods for the validation of weak protein-fragment interactions.[6] Here, we used the previously established TROSY NMR with ^{15}N-labeled LecA to rank binding of hydroxamates to LecA. For this, we derived the total number of chemical shift perturbations (CSPs) in ^{15}N LecA in the presence of **1** analogs and compared it to the positive control MeGal (**Figure S4a**). Briefly, we observed that the changes on the hydroxamic acid group (**3**, **7** and **8**) were not tolerated, whereas the compounds with a terminal benzyl group perturbed more residues in ^{15}N LecA compared to **1**, but less than MeGal. To elucidate the importance of the benzyl group, we replaced it with a methylcyclohexane group (**6**), which preserved its binding to LecA compared to a free hydroxamic acid (**18**). Moreover, the linker connecting both groups being flexible (**47**) or rigid (**42, 43**) were not tolerated and thus, resulted in two compounds (**2**, **35**) that preserved the binding to LecA (**Figures S4b-c**). Finally, the modifications on the linker position 2 (**29**) and benzyl group (**5**, **36**) are suitable for further fragment expansion. Interestingly, the structural re-scaffolding of hydroxamic acid to a cyclic form (**20**, **21**) preserved LecA binding, which has not been reported for metalloenzymes (MMPs) previously.[7] This is not surprising given the shallow binding site of LecA compared to rather deep active site pockets in metalloenzymes, which on the other hand require long and linear scaffolds as shown on example of marketed drugs for MMPs (**Table S3**, *Group 4*).

SPR analysis of hydroxamate derivatives

In order to establish structure-activity relationship (SAR) study of the hydroxamic acid derivatives by SPR, we assessed LecA binding capability of 27 commercial and 7 in-house synthetic hydroxamic acid derivatives by surface plasmon resonance (SPR). Binding responses of each compound were recorded at 0.2 and 1 mM to establish dose-

dependent responses. The binding responses were normalized as binding efficiency (%) to account for the molecular weight differences of the compounds and the amount of active immobilized LecA during the analyses (see the **Supplementary Materials and Methods** for normalization calculation). In agreement with TROSY NMR data, no binding response was observed when hydroxamic acid was replaced by amide (**3**), confirming that the binding to LecA was dependent on the presence of hydroxamic acid functional group.

Notably, compounds with a single terminal benzyl are predominant in our screen and elicit good dose-response and normalized binding responses. None of the in-house synthetic compounds showed positive dose-dependent binding responses, contradictory to the findings from NMR analyses (**Figures S5a-b**), whereas 8 of the commercial hydroxamic acid compounds (**4**, **9**, **11**, **15**, **26**, **27**, **29** and **35**) exhibited more than twice dose responses with the normalized binding responses greater than 10% (**Figure S5c**). This discrepancy emphasizes the importance of performing several orthogonal assays in the analysis of protein-fragment interaction. Especially, commercial compounds may contain impurities, such as metals, causing false-positive responses in SPR, which were taken care of in our in-house synthesized compounds.[8-9] Moreover, a rather weak affinity of hydroxamate derivatives was a limiting factor for employing SPR and thus, explaining the discrepancy with TROSY NMR results. Taken together, we concluded that SPR was not reliable to prioritize the hydroxamate derivatives and thus, other orthogonal methods were employed.

Competitive fluorescence polarization assay and PrOF NMR of LecA with hydroxamate derivatives

The hydroxamic acid derivatives were tested in a competitive binding assay (**Table S3**). The parent compound *N*-hydroxy-2-phenylacetamide (**2**) showed a low millimolar binding affinity to LecA in PrOF NMR (K_d = 6.1 ± 0.9 mM). We observed that the hydroxamic acid scaffold was essential for binding to the carbohydrate-binding site of LecA. The compounds, with methylated **3** or amide **7** lost their binding and inhibitory effect. A modification at the nitrogen atom was tolerated, but did not improve the binding affinity as shown by the methylated compound **8** (inh. = 20 ± 3%) compared to **2** (inh. = 18.3 ± 1%, K_d = 6.1 ± 0.9 mM). An interaction of LecA with the hydroxamic acid without the phenyl ring **18** could not be detected. None of the tested modifications on the phenyl ring of *N*-hydroxy-2-phenylacetamide **9**, **10**, **15**, **11** improved LecA binding (inh. = 14–16%). On the other hand, substitution of the phenyl ring with a cyclohexyl ring slightly improved the affinity (**6**, inh. = 21 ± 1%, K_d = 4.4 ± 0.6 mM). Notably, we observed that the optimal linker length between the phenyl ring and the hydroxamic acid moiety consists of 3 methylene groups (**35**, inh. = 26 ± 1%, K_d = 4.6 ± 0.9 mM). Longer linkers led to a binding decrease (**43**, inh. = 8 ± 3% and **44**, inh.= 17 ± 2%). Similarly, a diminished activity was observed for the rigid olefin (**42**, inh.= 13 ± 3%). Finally, substituted *N*-hydroxy-4-phenylbutanamide revealed that the electron donating substituents in *para* position could increase the binding affinity (-Me **36**, -OMe **40**, -OH **41**, inh. = 27–39%). On the other hand, the electron withdrawing groups dramatically decreased the potency (-NO_2 **39** inh. = 4 ± 2) indicating that a CH-π-stacking arises with LecA and the phenyl

ring. Interestingly, the 1-hydroxypyridin-2(1H)-one derivatives were as potent as *N*-hydroxy-4-phenylbutanamides (**19** inh. = 37 ± 2 and **20** inh. = 37 ± 1%), possibly due to the difference in predicted[10] pK_A values for the linear (**35** $pK_A \approx 9.4 \pm 0.2$) and cyclic hydroxamic acids with lower pK_A (**19** $pK_A \approx 6.0 \pm 0.1$) leading to the stronger chelating effects on the Ca^{2+} ion and hence increased affinity.

*Competitive ^{19}F T_2-filtered NMR with hydroxamate derivative **5***

We evaluated the selectivity and Ca^{2+}-dependency of the hydroxamate-LecA interaction and compared it to other Ca^{2+}-dependent lectins LecB, DC-SIGN and Langerin. For this, we used a hydroxamate derivative of **1** as a fluorinated reporter molecule (**5**) in a competitive ^{19}F NMR. Here, we tested 100 µM **5** binding in the absence (5 mM EDTA) and presence of 10 mM $CaCl_2$ alone or 10 µM lectins. Both Langerin and DC-SIGN demonstrated a Ca^{2+}-independent binding to **5**. For Langerin, the interaction persisted with and without $CaCl_2$ suggesting that **5** targeted a secondary site in Langerin. Notably, this interaction was weak, as it did not bind to ^{15}N-labeled Langerin CRD in ^{1}H-^{15}N HSQC NMR (**Figures S6a** and **S6c**). For DC-SIGN, we observed a partial recovery of the fluorine peak **5** in the presence of $CaCl_2$. Such behavior in ^{19}F NMR has been previously observed for fragments targeting the secondary sites in DC-SIGN.[11-12] Therefore, we validated **5** binding to DC-SIGN in ^{1}H-^{15}N HSQC and STD NMR (**Figures S6b** and **S6d**). Both assays revealed a very weak binding of **5** being partially competed with 30 mM D-mannose in STD NMR and fully competed with 5 mM EDTA, suggesting **5** bound to multiple sites in DC-SIGN. This is not surprising given the presence of an aryl ring in **5** besides the hydroxamic acid group, which has potentially interacted with a secondary site of DC-SIGN.

To rank the hydroxamate derivatives binding to LecA, we expected a stronger binding fragment to compete the reporter molecule **5** from the orthosteric site (**Figure S7**). In this study, **5** bound to LecA as shown by a decrease of the fluorine peak intensity, which recovered upon addition of 3 mM **35** and **2**, but not in the presence of the initial hit **1** or the negative control **3**. Given other 7 compounds showed rather a comparable competition, we concluded that **35** was the best ligand in this assay.

*Docking study of malonate **58** with metal-dependent lectins*

PA-IL (LecA)

Docking simulation of LecA (PDB: 4CP9) with compound **58** revealed that both carboxylates of the malonic acid fit in its carbohydrate-binding pocket (**Figures S10a-b**). Moreover, docking data suggested that only one carboxylate group of **58** interacted with the Ca^{2+} cofactor, whereas the other carboxylate could form a hydrogen bond with the water molecule buried in the pocket. This was in line with the SAR study using PrOF NMR, where compounds with only one carboxylate (**63**, **70**) failed to bind to the protein. Taken together, this indicated that the carbohydrate-binding site of LecA can accommodate two adjacent carboxylates (**58**) and that both of them are required: one to interact with Ca^{2+} ion, and one with the water molecule its carbohydrate-binding site of LecA.

PA-IIL (LecB)

The compound **58** was docked into the carbohydrate-binding site of LecB (PDB: 1OXC) resulting in two potential binding poses. The highest-ranking showed the pose with one carboxylate interacting with two Ca^2 ions in the carbohydrate-binding site of LecB, whereas the other carboxylate interacted with the protein surface through S22 and S23. Additionally, it revealed a potential interaction of T98 in LecB with the CF_2-group on the cyclopentyl group (**Figures 3a** and **S11b**). The second docking pose displayed both carboxylates interacting with the calcium atoms, as well as with the protein surface through G97 and S22 (**Figures 11a-b**). Together, the docking poses indicated that both carboxylates are required for binding being in agreement with the experimental data from the SAR study of **58** analogs, where compounds with one carboxylate moiety (**63**, **70**) failed to bind to the protein.

DC-SIGN CRD (CD209)

Docking simulation of DC-SIGN (PDB: 2XR5) revealed that **58** was able to coordinated the $Ca^{2+}{}_1$ ion in its carbohydrate binding-site through one carboxylate group, while the other carboxylate formed an H-bond with N344. Interestingly, a hydrogen atom of the cyclopentyl ring in alpha position to the CF_2 group was able to form a CH-π interaction with the aromatic ring of F313 (**Figure S12**).

Moreover, we observed **58** binding to a site bearing two Ca^{2+} ions in DC-SIGN CRD. However, **58** appeared to favor the binding to the carbohydrate-binding site of DC-SIGN CRD with $Ca^{2+}{}_1$ indicating that the interaction of **58** with DC-SIGN was not due to the sheer electrostatic forces alone. Moreover, the secondary binding site is more solvent exposed offering **58** less binding possibilities. Cumulatively, the malonate moiety alone does not provide the selectivity for lectin binding. However, the interactions with the amino acids neighboring the calcium ions can drive selective binding for malonate-containing fragments to the carbohydrate-binding site of DC-SIGN.

*Competitive ^{19}F T_2-filtered NMR with malonate derivative **61***

To investigate the Ca^{2+}-dependency and selectivity of the malonates-lectin interaction, we subjected metal-dependent lectins to binding studies in ^{19}F T_2-filtered NMR, where BambL was expected not to interaction with metal-binding pharmacophores (MBPs) such as malonates. Briefly, **61** bound to $CaCl_2$ alone as indicated by a chemical shift perturbation (CSP) of the fluorine peak in the presence of 10 mM $CaCl_2$, but not in its absence (5 mM EDTA). In the presence of $CaCl_2$ and 10 μM LecA, the fluorine peak decreased in the peak intensity demonstrating that this interaction was Ca^{2+}-dependent. We observed a similar pattern for LecB and DC-SIGN. For LecB, the competition experiment with 5 mM MeFuc showed a full recovery of the fluorine peak demonstrating that **58** targeted the carbohydrate-binding site of LecB. Moreover, TROSY NMR confirmed the Ca^{2+}-dependent binding of ^{15}N-labeled LecB to **58** followed by the displacement of **58** upon addition of EDTA (**Figure S13a**), which supported our ^{19}F NMR data. However, in case of 30 mM D-mannose and DC-SIGN, the fluorine peak of **61** recovered partially in the presence of the competitor. This observation was likely due to a weak affinity of 30 mM D-mannose for DC-SIGN (K_d = 3 mM compared to **61** (K_d = 1.9 mM) given **58** was displaced from DC-SIGN CRD upon addition of 10 mM EDTA in ^1H-

^{15}N HSQC NMR (**Figure S13b**). Further, the off-target effect of **58** to Langerin was observed as the fluorine peak decreased in the peak intensity in the presence of 5 mM EDTA suggesting that interaction was Ca^{2+}-independent. However, the **61** peak showed a stronger decrease in the presence of $CaCl_2$ and binding to BambL. Given the presence of the secondary sites in both lectins,[5, 11-12] we aimed to confirm that **61** targeted the secondary sites in both lectins. For this, we added a competitor (30 mM D-mannose and 10 mM MeFuc) expecting both ^{19}F peaks to remain unchanged. Indeed, competitors did not influence interactions with **61**, verifying its binding to a remote site in BambL and Langerin.

SAR study of malonate derivatives with LecB

Docking analysis proposed the interaction of **58** with the orthosteric site of LecB. Therefore, we investigated the derivatives of **58** for binding to ^{15}N LecB by TROSY NMR (**Figures S11c** and **S14**). We observed some resonances perturbed in ^{15}N LecB with 2 mM **58** and its analogs (**59**, **62**, **64**, **66** and **67**) in a similar manner to 1 mM MeFuc. Next, we determined the affinities (K_d) and LE values of the derivatives by performing a series of the titration experiments in TROSY NMR (**Figure S15**). Interestingly, all structural derivatives showed comparable affinities and LE values for ^{15}N LecB. Notably, **58**, **62** and **67** improved the binding to ^{15}N LecB unlike **64**, indicating the role of an electronegative group in the interaction. This supported the docking pose 1 of **58**, where LecB was predicted to interact with the CF_2 group of **58** through T98. Moreover, the methyl group introduced in **59** decreased its K_d and LE compared to **58**, suggesting that malonic moiety directly interacted with the protein surface and thus, a substituent in this position could disrupt this interaction. Therefore, we concluded that this position was not suitable for the fragment evolution. Furthermore, three compounds with a different scaffold (**66**, **67** and **69**) showed K_d values in a similar range to **58**. Their lower molecular weight and better LE values could render them superior starting points for fragment growing. Cumulatively, two main scaffold series have been identified for LecB: 1) **58**, **62** and **67**, and 2) **66**, **67** and **69**. Given the lack of ^{15}N LecB protein assignment, co-crystallization studies are currently ongoing to define the most potent scaffold for future fragment evolution.

SAR study of malonate derivatives with DC-SIGN

Similar to LecA and LecB, acetylated compounds (**63**, **65** and **70**) did not bind to ^{15}N DC-SIGN CRD (**Figure S16**). However, **58**, **62** and **67** affected the resonances in the EPN motif coordinating Ca_1^{2+} and D367 the strongest, whereas L321 and E324 near Ca_2^{2+} and Ca_3^{2+} showed weaker effects. Quantitative analyses of the conformational changes caused by **58** and D-mannose revealed a similar pattern of CSPs. Encouraged by these results, we derived the affinities of **58** analogs in ^{1}H-^{15}N HSQC NMR (**Figure S17**). Interestingly, all compounds showed a similar affinity and thus, three scaffold groups were defined as interchangeable (**58**, **62** and **69**). Similar to LecB, the compounds with an electronegative group on the ring being (**58**, **62, 63** and **67**) were predominant and thus, in agreement with the predicted F313 interaction of CF_2 group in **58**. In contrast to LecB, a methyl group in **59** was well tolerated in DC-SIGN CRD. Since **59** did not

interact with LecA either, this position is potentially suitable for future fragment growing to gain malonates specificity towards DC-SIGN. Together, both computational and experimental data demonstrated malonates' ability to target the Ca_1^{2+} binding site of DC-SIGN similarly to D-mannose.

Supplementary Materials and Methods

Virtual screening of drug-like molecules targeting LecA

Virtual screening (VS) of commercially available drug-like compounds was performed for LecA using the Bioinfo database (http://bioinfo-pharma.u-strasbg.fr/bioinfo/) v.18.1 applying the following filters: hydrogen bond acceptors (HBA) ≥2 and hydrogen bond donors (HBD) ≥2. Filter (OpenEye) was used to remove compounds with a poor pharmacokinetic profile and undesirable functional groups. Approximately 3 million molecules composed the VS library. LecA protein structure was retrieved from the Protein Data Bank (PDB): ID 1oko. One water molecule was conserved and retained in the carbohydrate-binding site of LecA. Other water molecules and heteroatoms were stripped from the structure leaving only the Ca^{2+} ion. Hydrogen atoms were added using PROTOSS.[13] Ligands were prepared for docking using Surflex[14] v. 3066 following the *pgeom* protocol (pose accuracy parameter set). For this, 20 poses were generated for each ligand and rescored using the GRIM[1] method, which is a knowledge-based approach used to score docking solutions by similarity of prediction interaction patterns to the ones in the PDB. The similarity was quantified using a score (GRIM score or GrSc) set to 0.7 or higher. The interaction patterns found in the virtual screening were compared to LecA-ligand complexes available in the PDB (**Table S1**). Hereby, we used the following filters: 1) the presence of the Ca^{2+} interactions, 2) the absence of 'carbohydrate-like scaffolds' namely the tetrahydrofuran or tetrahydropyran cores, 3) Surflex score ≥ 5, 4) a number of polar interactions (with the binding site residues) ≥3, and 5) a number of rings >0. Finally, the remaining ligands were clustered based on the maximum common substructure (MCS) using Chemaxon's LibMCS algorithm available in Pipeline Pilot[15] with a cutoff set to 8 (https://www.chemaxon.com). Later, 46 chemically diverse molecules were selected as hits and 37 were purchased for testing.

Virtual screening of drug-like molecules targeting LecB

Virtual screening (VS) of commercially available drug-like compounds was performed for LecB with an updated version of Bioinfo database v.18.2 following the same filtering criteria as described for LecA. Ligands were prepared as described in the section above. LecB protein structure was retrieved from the Protein Data Bank (PDB): ID 1gzt. In addition to the two conserved Ca^{2+} ions, a conserved water molecule bridging interactions between α-L-fucose O1 and O2 atoms and residues Thr98 and Asp99 was retained in the docking calculations. In addition to PDB ID: 1gzt, 13 PDB structures were selected for calculation of the similarity interaction patterns using GRIM method (**Table S2**). All co-crystallized ligands and other heteroatoms, excepting the Ca^{2+} ion and the

conserved water molecule were striped from the protein structures. Hydrogen atoms were added using PROTOSS. Docking was performed using Surflex with the same parameters described for LecA. The post-processing of the docked poses followed the workflow similar to the carbohydrate-binding site of LecA, with GrSc cutoff set to ≥0.7, and clustering by MCS with same parameters described above. Other filters used here included: 1) a number of polar interactions ≥3, 2) SurflexScore ≥5, 3) a number of rings >0, 4) a number of rotatable bonds ≤10 and 5) a number of aromatic rings <5. Additionally, we used the shape matching software ROCS[16] to select the VS hits with a Tanimoto shape-similarity to the LecB-fucose complex higher than 0.6 resulting in a total of 42 hits for LecB.

Crystallographic study

The recorded crystallographic data were indexed, integrated, and scaled using XDS[17] and merged using AIMLESS[18]. The structures were solved by molecular replacement using 1OKO as a searching template in PHASER[19], followed by further iterations of manual rebuilding in COOT[20] and restrained refinement in REFMAC5[21]. Hydroxamic acid ligand was manually built in ACEDRG[22] in CCP4i2 suite[23]. The final model was validated with MOLPROBITY[24], PDB-redo (https://PDB-redo.eu/) and wwPDB validation service (http://validate-rcsb-1.wwPDB.org/) prior to submission to the Protein Data Bank. All structural figures were prepared using CCP4MG[25]. Data processing, refinement statistics and PDB ID of the deposited structure are provided in the **Table S5**.

Chemical synthesis of hydroxamate derivatives

All reactions were performed under inert gas (N_2) by using the Schlenk technique. The chemicals and solvents were bought from TCI, Merck or Roth and used without further purifications. The reactions were followed by either TLC (aluminum plates coated with silica gel 60, Merck KGaA, Damstadt, Germany) by using molybdenum-stain (0.02 M solution of $Ce(NH_4)_4(SO_4)_4 \cdot 2H_2O$ and $(NH_4)_6Mo_7O_{24} \cdot 4H_2O$ in aqueous 10% H_2SO_4) or by HPLC-MS (Thermo Dionex Ultimate 3000 HPLC coupled to a Bruker amaZon SL mass spectrometer, with UV detection at 254 nm, using a C18 column (100/2 Nucleoshell RP18plus, 2.7 µm from Macherey-Nagel, Germany). The crude products were purified by MPLC (Teledyne Isco Combiflash Rf200) by using self-packed silica gel 60 columns (60Å, 400 mesh particle size, Fluka) as a stationary phase or by HPLC (Waters 2545 Binary Gradient Module with Waters 2489 UV/Visible detector) using RP-18 column (250/21 Nucleodur C18 Gravity SB, 5 µm from Macherey-Nagel, Germany). The synthesized compounds were analyzed by NMR spectroscopy (Bruker Avance III 500 ultra shield spectrometer) at 500 MHz (^1H) or 126 MHz (^{13}C) using deuterated solvents (Eurisotop, Saarbrücken, Germany) and analyzed with MestReNova (Version 12.0.2). Chemical shifts are given in parts per million compared to an internal solvent peak (MeOH-d_4 = 3.31, 49.00 ppm, DMSO-d_6 = 2.50, 39.52 ppm)[26] and the multiplicities as s (singlet), d (doublet), t (triplet), q (quartet) and m (multiplet). The high-resolution mass spectroscopy was recorded on an Ultimate 3000 UPLC system connected to a Q Exactive Focus Orbitrap system with HESI source (Thermo Fisher, Dreieich, Germany).

UPLC: C18 column (EC 150/2 Nucleodur C18 Pyramid, 3 µm, Macherey-Nagel, Germany).

*N,3-Dihydroxy-3-(3-methoxyphenyl)propenamide (**31**)*

The previously reported procedure of *N*,3-dihydroxy-3-(3-methoxyphenyl)propenamide (**31**) was slightly modified and optimized.[27] A: Zinc powder was activated by washing in a 1N HCl bath for 30 min, then filtrated washed with H_2O, EtOH, Et_2O and dried on high vacuum for 20 min. B: The activated zinc powder (653.8 mg, 10 mmol, 2.0 eq.) was dissolved in dry THF (8 mL) and refluxed for 5 min. The heating was stopped and a solution of ethyl bromoacetate (1 g, 6 mmol, 1.2 eq.) and *m*-anisaldehyde (680.75 mg, 5 mmol, 1.0 eq.) in dry THF (5 mL) was carefully added over 30 min (syringe pump). Then, the mixture was refluxed for 16 h. The reaction was stopped by adding NH_4Cl (20 mL) and stirred for additional 15 min. The phases were separated and the aq. phase extracted with EtOAc (3x). The combined org. phase was dried over Na_2SO_4, concentrated, purified by flash chromatography (PE:EtOAc - 8:2) and re-purified with Tol:EtOAc - 9:1. The ester intermediate was obtained as an clear oil (660 mg, 2.94 mmol, 59%, R_f = 0.35, PE: EtOAc – 8:2). C: Hydroxylamine hydrochloride (217 mg, 1.66 eq) was dissolved in MeOH (2.76 mL) and a NaOMe solution (5.66 M, 0.98 mL, 3.33 eq.) was added. The milky mixture was stirred for 15 min and the previously synthesized ester (421.2 mg, 1.88 mmol, 1.0 eq) in MeOH (1 mL, c_{final} = 0.5M) was added carefully. The reaction was stopped by adjusting the pH to 7 with 1 M HCl in 5 hours (ca. 20 drops) and H_2O was added. The mixture was diluted with H_2O and the aq. phase was extracted with EtOAc (3x). The combined org. phases were dried over Na_2SO_4 and the product was purified by flash chromatography (PE: EE, R_f = 0.4 pure EtOAc). The product was obtained as a clear oil which solidifies (128.1 mg, 0.50 mmol, 30%, Keto E/Z = 89:10). ^{1}H NMR (500 MHz, DMSO-d_6) δ 10.33 (s, 1H, Keto-E O<u>H</u>), 9.86 (s, 1H, Keto-Z O<u>H</u>), 9.07 (s, 1H, Keto-Z N<u>H</u>), 8.73 (s, 1H, Keto-E N<u>H</u>), 7.22 (t, *J* = 8.0 Hz, 1H, Ar<u>H</u>), 6.92 – 6.86 (m, 2H), Ar<u>H</u> 2x), 6.79 (ddd, *J* = 8.2, 2.6, 1.0 Hz, 1H, Ar<u>H</u>), 5.37 (d, *J* = 4.6 Hz, 1H, CHO<u>H</u>), 4.91 (dt, *J* = 8.6, 4.4 Hz, 1H, C<u>H</u>OH), 3.74 (s, 3H, OC<u>H</u>$_3$), 2.33 – 2.20 (m, 2H, C<u>H</u>$_2$). ^{13}C NMR (126 MHz, DMSO) δ 166.85 (<u>C</u>=O), 159.13 (Ar<u>C</u>), 147.07 (Ar<u>C</u>), 129.12 (Ar<u>C</u>H), 117.91 (Ar<u>C</u>H), 112.29 (Ar<u>C</u>H), 111.21 (Ar<u>C</u>H), 69.29 (<u>C</u>H), 54.93 (O<u>C</u>H$_3$), 42.76 (<u>C</u>H$_2$). LR-MS calcd $[C_{10}H_{14}NO_4]^+$: 212.09, found 212.17. The spectroscopic data differed slightly from the literautre.[36]

*2-Cyclohexyl-N-hydroxyacetamide (**6**)*

The synthesis of 2-cyclohexyl-*N*-hydroxyacetamide (6) was performed as described before by Ohtsuka et al.[28] Solutions of $H_2NOH{\cdot}HCl$ (445 mg, 6.4 mmol) in dry MeOH (3.2 mL, *c* = 2 M) and KOH (71.8 mg, 0.4 eq.) in MeOH (3.2 mL, c = 0.4) were prepared. Both solutions were cooled to 0 °C, then, the alkali solution was added to the stirred hydroxylamine and the resulting suspension was left without stirring for 5 min. The white precipitate (KCl) was removed by suction filtration and the clear filtrate was added to methyl cyclohexylacetate (500 mg, 3.20 mmol). KOH (2 pellets) was added until pH = 10 was reached and the mixture was stirred overnight. The solvent was removed and the crude was diluted in H_2O leading to white precipitate. The solution was acidified pH < 4 and the product was filtrated, resulting in a white solid (144 mg, 0.917 mmol, 29%, E/Z =

88:8, CH_2Cl_2/ MeOH – 19:1, R_f = 0.57). ^{1}H NMR (500 MHz, DMSO-d_6) δ 10.30 (br s, 1H, Keto-E OH), 9.72 (s, 1H, Keto-Z OH), 8.96 (s, 1H, Keto-Z NH) 8.65 (d, J = 1.7 Hz, 1H, Keto-E NH), 1.81 (d, J = 6.8 Hz, 2H, CH_2), 1.72 – 1.54 (m, 6H, CH_2 2.5x, CH), 1.26 – 1.04 (m, 3H, CH_2 1.5x), 0.95 – 0.82 (m, 2H, CH_2). ^{13}C NMR (126 MHz, DMSO) δ 168.19 (C=O), 39.78 (CH_2), 34.45 (CH), 32.47 (CH_2 2x), 25.83 (CH_2), 25.57 (CH_2 2x). HR-MS calcd $[C_8H_{16}NO_2]^+$: 158.1176, found 158.1174. The spectroscopic data match the literature.[29]

*N-Methoxy-2-phenylacetamide (**7**)*

N-Methoxy-2-phenylacetamide (**7**) was synthesized according to the previously reported procedure by Kawase.[30] Methoxyamine hydrochloride (446 mg, 5.3 mmol, 1.1 eq.) and Na_2CO_3 (1.03 g, 9.7 mmol, 2.0 eq.) were dissolved in a mixture of toluene and H_2O (1:1, c ≈ 0.25 M) and cooled to 0 °C. Phenylacetyl chloride (641 µL, 4.8 mmol, 1.0 eq.) was added and the reaction was stirred for 20 h. The reaction was taken up in EtOAc, separated, and the org. phase was washed with brine and dried over Na_2SO_4. The crude product was purified by flash chromatography (PE:EE – 1:2, R_f = 0.3) and compound **7** was obtained as a white solid (487 mg, 2.95 mmol, 61%). ^{1}H NMR (500 MHz, DMSO-d_6) δ 11.25 (s, 1H, NH), 7.34 – 7.27 (m, 2H, ArH 2x), 7.27 – 7.20 (m, 3H, ArH 3x), 3.57 (s, 3H, OCH_3), 3.28 (s, 2H, CH_2). ^{13}C NMR (126 MHz, DMSO) δ 166.91 (C=O), 135.52 (ArC), 128.93 (ArCH 2x), 128.30 (ArCH 2x), 126.56 (ArCH), 63.20 (OCH_3), 39.25 (CH_2). HR-MS calcd $[C_9H_{12}NO_2]^+$: 166.0863, found 166.0860. The spectroscopic data are in accordance with the literature.[31]

*N-Hydroxy-N-methyl-2-phenylacetamide (**8**)*

N-Hydroxy-*N*-methyl-2-phenylacetamide (**8**) was synthesized by following the produce of Clark et al.[32] *N*-Methylhydroxylamine hydrochloride (500 mg, 5.98 mmol, 1.0 eq.) was dissolved in CH_2Cl_2 (c = 0.5 M) and cooled to 0 °C. Et_3N (1.66 mL, 2.0 eq.) was added and the mixture was stirred for 10 min, followed by phenylacetyl chloride drop-wise (≈ 797 µL, 5.98 mmol, 1.0 eq.). The mixture was stirred at room temperature for 16 h, then washed with 1 N HCl and brine. The org. phase was dried over Na_2SO_4, concentrated under vacuum and the yellow crude material was purified by flash chromatography (PE: EtOAc – 1:2, R_f = 0.43). The product was obtained as a colorless oil (314.7 mg, 1.90 mmol, 32%). ^{1}H NMR (500 MHz, DMSO-d_6) δ 9.98 (s, 1H, NH), 7.34 – 7.25 (m, 2H, ArH 2x), 7.25 – 7.18 (m, 3H, ArH 3x), 3.69 (s, 2H CH_2), 3.10 (s, 3H, CH_3). ^{13}C NMR (126 MHz, DMSO) δ 170.77 (C=O), 135.92 (ArC), 129.43 (ArCH 2x), 128.11 (ArCH 2x), 126.24 (ArCH), 38.32 (CH_2), 35.80 (CH_3). HR-MS calcd $[C_9H_{12}NO_2]^+$: 166.0863, found 166.0861.

*General procedure for **35** to **44***

The different hydroxamic acids **35** to **44** were synthesized according to a modified procedure of Trabulsi *et al.* starting from an acid moiety.[33] The corresponding acid (1eq.) was dissolved in dry CH_2Cl_2 (c = 0.25 M) and cooled to 0 °C. Oxalyl chloride (1.75 eq.) was added dropwise and the mixture was allowed to warm to room temperature and stirred for 16 h (yellowish). The solvent was removed und reduced pressure and an equimolar solution of hydroxyl amine (7 eq.) and NaOH (7 eq.) in H_2O (c = 2.5 M) was

added. The mixture was left without stirring for 15 min (until the turbidity vanished) and the mixture was diluted with EtOAc, separated and the aq. phase was extracted with EtOAc (3x). The combined org. phases were dried over Na_2SO_4, filtered and concentrated under reduced pressure and purified by flash chromatography (CH_2Cl_2 MeOH – 19:1) resulting in white solids.

*N-Hydroxy-4-phenylbut-3-enamide (**42**)*

N-Hydroxy-4-phenylbut-3-enamide (**42**) was synthesized according to the general procedure starting from *trans*-styrylacetic acid (200 mg, 1.23 mmol, 1.0 eq.). A white solid was obtained (80 mg, 0.49 mmol, 40%, CH_2Cl_2/ MeOH – 19:1, R_f = 0.49, Keto-E/Z: 89:11). ^{1}H NMR (500 MHz, DMSO-d_6) δ 10.51 (s, 1H, Keto-E O<u>H</u>), 9.94 (s, 1H, Keto-Z O<u>H</u>), 9.19 (s, 1H, Keto-Z N<u>H</u>), 8.79 (s, 1H, Keto-E N<u>H</u>), 7.43 – 7.37 (m, 2H, ArC<u>H</u> 2x), 7.32 (dd, J = 8.5, 6.9 Hz, 2H, ArC<u>H</u> 2x), 7.26 – 7.20 (m, 1H, ArC<u>H</u>), 6.51 – 6.43 (m, 1H, C<u>H</u>CHCH$_2$), 6.29 (dt, J = 15.9, 7.1 Hz, 1H, CHC<u>H</u>CH$_2$), 2.92 (dd, J = 7.1, 1.5 Hz, 2H, CHCHC<u>H$_2$</u>). ^{13}C NMR (126 MHz, DMSO) δ 166.93 (<u>C</u>=O), 136.77 (Ar<u>C</u>), 132.09 (C<u>H</u>CHCH$_2$), 128.62 (Ar<u>C</u>H 2x), 127.33 (Ar<u>C</u>H), 125.98 (Ar<u>C</u>H 2x), 124.07 (CHC<u>H</u>CH$_2$), 36.84 (CHCH<u>C</u>H$_2$). HR-MS calcd $[C_{10}H_{12}NO_2]^+$: 178.0863, found 178.0860.

*N-Hydroxy-2-phenethoxyacetamide (**43**)*

The product was obtained from 2-phenethoxyacetic acid (100 mg, 0.55 mmol, 1.0 eq.) as a white solid (38.7 mg, 0.198 mmol, 36%, CH_2Cl_2/MeOH – 19:1, R_f = 0.575, Keto-E/Z = 92:7). ^{1}H NMR (500 MHz, DMSO-d_6) δ 10.48 (s, 1H, Keto-E O<u>H</u>), 9.96 (s, 1H, Keto-Z O<u>H</u>), 9.00 (s, 1H, Keto-Z N<u>H</u>), 8.83 (s, 1H, Keto-E N<u>H</u>), 7.32 – 7.22 (m, 4H, Ar<u>H</u> 4x), 7.22 – 7.17 (m, 1H, Ar<u>H</u>), 3.84 (s, 2H, C<u>H$_2$</u>), 3.62 (t, J = 7.0 Hz, PhCH$_2$C<u>H$_2$</u>O), 2.83 (t, J = 7.0 Hz, 1H, PhC<u>H$_2$</u>CH$_2$O). ^{13}C NMR (126 MHz, DMSO) δ 165.59 (<u>C</u>=O), 138.77 (Ar<u>C</u>), 128.86 (Ar<u>C</u>H 2x), 128.25 (Ar<u>C</u>H 2x), 126.09 (Ar<u>C</u>H), 71.73 (PhCH$_2$<u>C</u>H$_2$O), 68.68 (<u>C</u>H$_2$), 35.24 (PhC<u>H$_2$</u>CH$_2$O). HR-MS calcd $[C_{10}H_{14}NO_3]^+$: 196.0968, found 196.0967.

*N-Hydroxy-2-(3-phenylpropoxy)acetamide (**44**)*

The desired compound was obtained from 2-(3-phenylpropoxy)acetic acid (100 mg, 0.51 mmol, 1.0 eq.) as a white solid (35 mg, 0.18 mmol, 35%, CH_2Cl_2/ MeOH - 19:1, R_f = 0.85, Keto-E/Z = 93:9). ^{1}H NMR (500 MHz, DMSO-d_6) δ 10.50 (s, 1H, Keto-E O<u>H</u>), 9.95 (s, 1H, Keto-Z O<u>H</u>), 9.00 (s, 1H, Keto-Z N<u>H</u>), 8.82 (s, 1H, Keto-E N<u>H</u>), 7.27 (t, J = 7.5 Hz, 2H, ArC<u>H</u>), 7.24 – 7.14 (m, 3H, ArC<u>H</u>), 3.81 (s, 2H, C<u>H$_2$</u>), 3.41 (t, J = 6.4 Hz, 2H, PhCH$_2$CH$_2$C<u>H$_2$</u>O), 2.69 – 2.57 (m, 2H, PhC<u>H$_2$</u>CH$_2$CH$_2$O), 1.89 – 1.76 (m, 2H, PhCH$_2$C<u>H$_2$</u>CH$_2$O). ^{13}C NMR (126 MHz, DMSO) δ 165.65 (C=O), 141.79 (Ar<u>C</u>), 128.33 (Ar<u>C</u>H 2x), 128.29 (Ar<u>C</u>H 2x), 125.72 (Ar<u>C</u>H), 70.12 (PhCH$_2$CH$_2$<u>C</u>H$_2$O), 68.77 (<u>C</u>H$_2$), 31.56 (Ph<u>C</u>H$_2$CH$_2$CH$_2$O), 30.80 (PhCH$_2$<u>C</u>H$_2$CH$_2$O). HR-MS calcd $[C_{11}H_{16}NO_3]^+$: 210.1125, found 210.1121.

*N-Hydroxy-4-phenylbutanamide (**35**)*

This product was obtained from 4-phenylbutric acid (200 mg, 1.22 mmol) as a white solid (131 mg, 0.73 mmol, 60%, CH_2Cl_2/MeOH – 19:1, R_f = 0.45, Keto-E/Z 85:14). ^{1}H NMR (500 MHz, DMSO-d_6) δ 10.35 (s, 1H, NHOH), 9.76 (s, 1H, Keto-Z O<u>H</u>, 8.99 (s, 1H, Keto-Z N<u>H</u>) 8.68 (s, 1H, NHOH), 7.28 (dd, J = 8.2, 6.9 Hz, 2H, ArH (3x)), 7.23 – 7.15 (m, 3H,

ArH (3x)), 2.54 (t, J = 7.7 Hz, 2H, PhC$\underline{H}_2CH_2CH_2$CO), 1.96 (t, J = 7.4 Hz, 2H, PhCH$_2$CH$_2$C$\underline{H}_2$CO), 1.82 – 1.73 (m, 2H, PhCH$_2$C$\underline{H}_2$CH$_2$CO). ^{13}C NMR (126 MHz, DMSO) δ 168.86 ($\underline{C}$=O), 141.66 (ArC), 128.31 (Ar$\underline{C}$H, 4x), 125.79 (Ar$\underline{C}$H 1x), 34.60 (Ph$\underline{C}$H$_2$CH$_2$CH$_2$CO), 31.78 (PhCH$_2$CH$_2\underline{C}$H$_2$CO), 26.99 (PhCH$_2\underline{C}$H$_2$CH$_2$CO). HR-MS calcd $[C_{10}H_{14}NO_2]^+$: 180.1019, found 180.1017. The spectroscopic data are in accordance with the literature.[29]

*N-Hydroxy-4-(p-tolyl)butanamide (**36**)*
The desired compound was obtained from 4-(*p*-tolyl)butanoic acid (200 mg, 1.12 mmol) after purification with CH_2Cl_2/MeOH – 19:1 (R_f = 0.22) as a white solid (177 mg, 0.92 mmol, 82%, E/Z= 85:11). The compound was further purified by prep-HPLC (CH_3CN/H_2O) for biological tests. ^{1}H NMR (500 MHz, DMSO-d_6) δ 10.34 (s, 1H, Keto-E O$\underline{H}$), 9.75 (s, 1H, Keto-Z-OH), 8.68 (s, 1H, Keto-E N$\underline{H}$), 7.11 – 7.04 (m, 4H, ArC$\underline{H}$(4x)), 2.52 – 2.50 (m, 2H, PhC$\underline{H}_2$CH$_2$CH$_2$CO), 2.26 (s, 1H, PhCH$_2$CH$_2$C$\underline{H}_2$CO), 1.95 (t, J = 7.5 Hz, 2H), 1.79 – 1.70 (m, 2H, PhCH$_2\underline{C}$H$_2$CH$_2$CO). ^{13}C NMR (126 MHz, DMSO) δ 169.36 (C=O), 138.98 (ArC), 135.10 (ArC), 129.34 ArCH (2x)), 128.65 (ArCH (2x)), 34.64 (Ph$\underline{C}$H$_2$CH$_2$CH$_2$CO), 32.23 (PhCH$_2$CH$_2\underline{C}$H$_2$CO), 27.53 (PhCH$_2\underline{C}$H$_2$CH$_2$CO), 21.09 (ArC$\underline{H}_3$). HR-MS calcd $[C_{11}H_{16}NO_2]^+$: 194.1176, found 194.1174.

*N-Hydroxy-4-(thiophen-2-yl)butanamide (**37**)*
This compound was obtained from 4-(thiophen-2-yl)butanoic acid (200 mg, 1.18 mmol) after purification with CH_2Cl_2/MeOH – 19:1 (R_f = 0.50) as a slightly orange solid (62.2 mg, 0.33 mmol, 29%, E/Z = 84:16). ^{1}H NMR (500 MHz, DMSO-d_6) δ 10.37 (s, 1H, Keto-E O$\underline{H}$), 9.79 (s, 1H, Keto-Z OH), 9.01 (s, 1H, Keto-Z NH), 8.69 (s, 1H, Keto-E N$\underline{H}$), 7.32 (d, J = 1.2 Hz, 1H, thiophene-H), 6.93 (d, J = 5.2 Hz, 1H, thiophene-H), 6.84 (d, J = 1.1 Hz, 1H, thiophene-H), 2.77 (s, 2H, thiopheneC$\underline{H}_2$CH$_2$CH$_2$CO), 2.00 (d, J = 7.4 Hz, 2H, thiopheneCH$_2$CH$_2$C$\underline{H}_2$CO), 1.82 (s, 2H, thiopheneCH$_2$C$\underline{H}_2$CH$_2$CO). ^{13}C NMR (126 MHz, DMSO) δ 169.13 ($\underline{C}$=O), 144.65 (thiophene-$\underline{C}$), 127.40 thiophene-$\underline{C}$H, 125.01 thiophene-$\underline{C}$H, 124.03 thiophene-$\underline{C}$H, 32.00 (thiophene$\underline{C}$H$_2$CH$_2$CH$_2$CO), 29.10 (thiopheneCH$_2$CH$_2\underline{C}$H$_2$CO), 27.83 (thiopheneCH$_2\underline{C}$H$_2$CH$_2$CO). HR-MS calcd $[C_8H_{12}NO_2S]^+$: 186.0583, found 186.0580.

*4-(4-Bromophenyl)-N-hydroxybutanamide (**38**)*
This compounds was obtained from 4-(4-bromophenyl)butanoic (200 mg, 0.82 mmol) after purification with CH_2Cl_2/MeOH – 19:1 (R_f = 0.19) as a white solid (100.8 mg, 0.39 mmol, 48%, E/Z= 87:13) and was further purified by prep-HPLC (CH_3CN/H_2O) for biological tests. ^{1}H NMR (500 MHz, DMSO-d_6) δ 10.35 (s, 1H, Keto-E O$\underline{H}$), 9.77 (s, 1H, Keto-Z OH), 8.98 (s, 1H, Keto-Z NH), 8.69 (s, 1H, Keto-E N$\underline{H}$), 7.57 – 7.29 (m, 2H (ArC$\underline{H}$ (2x)), 7.29 – 6.90 (m, 2H, ArC$\underline{H}$ (2x)), 2.53 (d, J = 7.6 Hz, 2H, PhC$\underline{H}_2$CH$_2$CH$_2$CO), 1.94 (t, J = 7.4 Hz, 2H, PhCH$_2$CH$_2$C$\underline{H}_2$CO), 1.86 – 1.53 (m, 2H, PhCH$_2$C$\underline{H}_2$CH$_2$CO). ^{13}C NMR (126 MHz, DMSO) δ 168.74 (C=O, 141.09 (Ar$\underline{C}$), 131.14 (Ar$\underline{C}$H), 130.63 (Ar$\underline{C}$H), 118.82 (Ar$\underline{C}$), 33.84 (Ph$\underline{C}$H$_2$CH$_2$CH$_2$CO), 31.59 (PhCH$_2$CH$_2\underline{C}$H$_2$CO), 26.71 (PhCH$_2\underline{C}$H$_2$CH$_2$CO). HR-MS calcd $[C_{10}H_{13}BrNO_2]^+$: 258.0124, found 258.0119.

*N-Hydroxy-4-(4-nitrophenyl)butanamide (**39**)*

The product was obtained from 4-(4-nitrophenyl)butanoic acid (200 mg, 0.96 mmol) after purification with CH_2Cl_2/MeOH – 19:1 (R_f = 0.19) as a white solid (166 mg, 0.74 mmol, 77%, E/Z= 87:13). This compound was further purified for biological tests by prep-HPLC (CH_3CN/ H_2O). ^1H NMR (500 MHz, DMSO-d_6) δ 10.55 – 10.18 (m, 1H, Keto-E O$\underline{H}$), 9.81 (s, 1H, Keto-Z OH), 9.01 (s, 1H, Keto-Z NH), 8.71 (s, 1H, Keto-E N$\underline{H}$), 8.15 (d, *J* = 8.5 Hz, 2H, ArC$\underline{H}$ (2x)), 7.48 (d, *J* = 8.3 Hz, 2H, (ArC$\underline{H}$ (2x)), 2.70 (t, *J* = 7.7 Hz, 2H, PhC$\underline{H}_2$CH$_2$CH$_2$CO), 1.98 (t, *J* = 7.4 Hz, 2H, PhCH$_2$CH$_2$C$\underline{H}_2$CO), 1.83 (t, *J* = 7.3 Hz, 2H, PhCH$_2$C$\underline{H}_2$CH$_2$CO). ^{13}C NMR (126 MHz, DMSO) δ 168.61 ($\underline{C}$=O), 150.18 (Ar$\underline{C}$), 145.89 (Ar$\underline{C}$), 129.65 (Ar$\underline{C}$H (2x)), 123.48 (Ar$\underline{C}$H (2x)), 34.29 (Ph$\underline{C}$H$_2$CH$_2$CH$_2$CO), 31.55 (PhCH$_2$CH$_2\underline{C}$H$_2$CO), 26.39 (PhCH$_2\underline{C}$H$_2$CH$_2$CO). HR-MS calcd $[C_{10}H_{13}N_2O_4]^+$: 225.0870, found 225.0865.

*N-Hydroxy-4-(4-methoxyphenyl)butanamide (**40**)*

The product was obtained from 4-(4-methoxyphenyl)butanoic (200 mg, 1.03 mmol) as a white solid (81 mg, 0.39 mmol, 38%, E/Z= 83:17, R_f = 0.50, CH_2Cl_2/MeOH – 19:1). ^1H NMR (500 MHz, DMSO-d_6) δ 10.34 (s, 1H, Keto-E O$\underline{H}$), 9.75 (s, 1H Keto-Z OH), 8.98 (s, 1H, Keto-Z NH), 8.67 (s, 1H, Keto-E N$\underline{H}$), 7.09 (d, *J* = 8.5 Hz, 2H, ArCH (2x)), 6.84 (d, *J* = 8.6 Hz, 2H, ArCH (2x)), 3.71 (s, 3H, OC$\underline{H}_3$), 2.47 (d, *J* = 7.8 Hz, 2H, PhC$\underline{H}_2$CH$_2$CH$_2$CO), 1.94 (t, *J* = 7.5 Hz, 2H, PhCH$_2$CH$_2$C$\underline{H}_2$CO), 1.86 – 1.61 (m, 2H, PhCH$_2$C$\underline{H}_2$CH$_2$CO). ^{13}C NMR (126 MHz, DMSO) δ 168.91 ($\underline{C}$=O), 157.42 (Ar$\underline{C}$), 133.49 (Ar$\underline{C}$), 129.21 (Ar$\underline{C}$H (2x)), 113.71 (Ar$\underline{C}$H (2x)), 54.96 (O$\underline{C}$H$_3$), 33.70 (Ph$\underline{C}$H$_2$CH$_2$CH$_2$CO), 31.73 (PhCH$_2$CH$_2\underline{C}$H$_2$CO), 27.23 (PhCH$_2\underline{C}$H$_2$CH$_2$CO). HR-MS calcd $[C_{11}H_16NO_3]^+$: 210.1125, found 210.1121.

*N-Hydroxy-4-(4-hydroxyphenyl)butanamide (**41**)*

The reaction was performed in analogy to a previously reported procedure.[34] *N*-hydroxy-4-(4-methoxyphenyl)butanamide (15.8 mg, 0.076 mmol, 1 eq.) was dissolved in dry CH_2Cl_2 and cooled to -78 °C. BBr_3 (380 µL, 0.38 mmol, 5 eq.) was added and the reaction was warmed to room temperature and stirred for 3 days (CH_2Cl_2/MeOH – 19:1 R_f = 0.28). The reaction was taken up in aqueous saturated $KHSO_4$ and extracted with EtOAc (3x). The combined org. phases were dried over Na_2SO_4 and concentrated under reduced pressure. The crude product was purified by prep-HPLC (CH_3CN/ H_2O) to give the product as a white solid (3.2 mg, 16 µmol, 22%, E/Z= 84:16). ^1H NMR (500 MHz, DMSO-d_6) δ 10.33 (s, 1H, Keto-E O$\underline{H}$), 9.74 (s, 1H, Keto-Z OH), 9.12 (s, 1H, ArO$\underline{H}$), 8.97 (s, 1H, Keto-Z-NH), 8.66 (s, 1H, Keto-E N$\underline{H}$), 6.95 (d, *J* = 8.4 Hz, 2H, ArC$\underline{H}$ (2x)), 6.66 (d, *J* = 8.4 Hz, 2H, ArC$\underline{H}$ (2x)), 2.42 (t, *J* = 7.6 Hz, 2H, PhC$\underline{H}_2$CH$_2$CH$_2$CO), 1.93 (t, *J* = 7.5 Hz, 2H, PhCH$_2$CH$_2$C$\underline{H}_2$CO), 1.76 – 1.67 (m, 2H, PhCH$_2$C$\underline{H}_2$CH$_2$CO). ^{13}C NMR (126 MHz, DMSO) δ 168.97 (C=O), 155.35 (Ar$\underline{C}$), 131.67 (Ar$\underline{C}$), 129.12 (Ar$\underline{C}$H (2x)), 115.04 (Ar$\underline{C}$H (2x)), 33.78 (Ph$\underline{C}$H$_2$CH$_2$CH$_2$CO), 31.77 (PhCH$_2$CH$_2\underline{C}$H$_2$CO), 27.31 (PhCH$_2\underline{C}$H$_2$CH$_2$CO). HR-MS calcd $[C_{10}H_{14}NO_3]^+$: 196.0968, found 196.0965.

Supplementary Figures

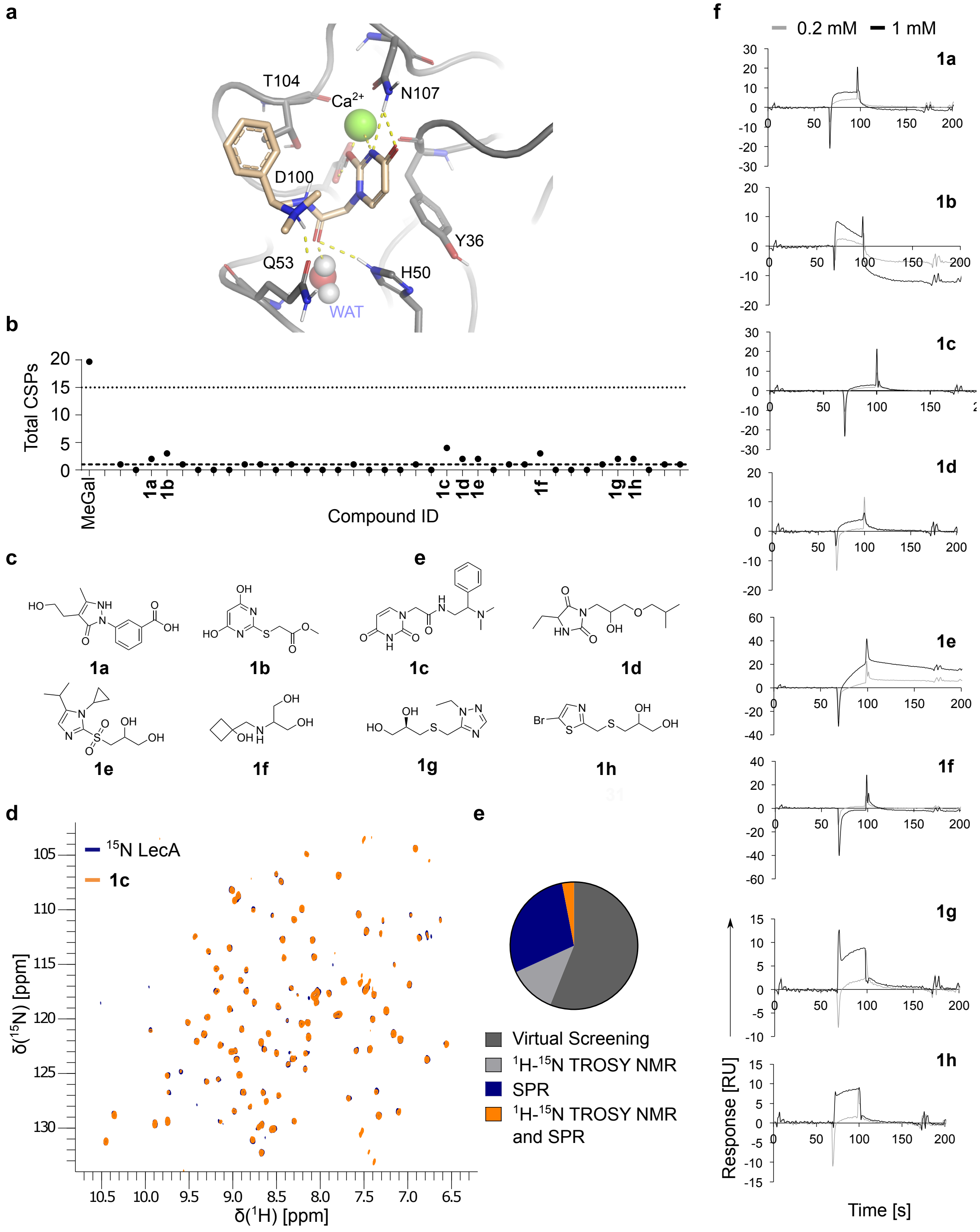

Figure S1 *Virtual screening of fragment and drug-like libraries for LecA.*
(**a**) Shown is the binding mode of **1c** to the carbohydrate-binding pocket of LecA.
(**b**) Virtual screening hits were validated in TROSY NMR. The plot shows the total number of chemical shift perturbations (CSPs) that were promoted in ^{15}N LecA in the presence of 2 mM hits. (**c**) The structures of fragments binding LecA similarly to MeGal above the threshold (*dashed line*) shown in (**b**). (**d**) TROSY NMR spectrum of 0.15 mM ^{15}N LecA in the presence of 2 mM **1c** shows only small changes in protein upon fragment addition. (**e**) Overview of 36 LecA virtual screening hits, whereas 8 were tested positive in TROSY NMR, 19 in SPR and 2 in both assays. (**f**) SPR analysis of the most promising hits identified in TROSY NMR.

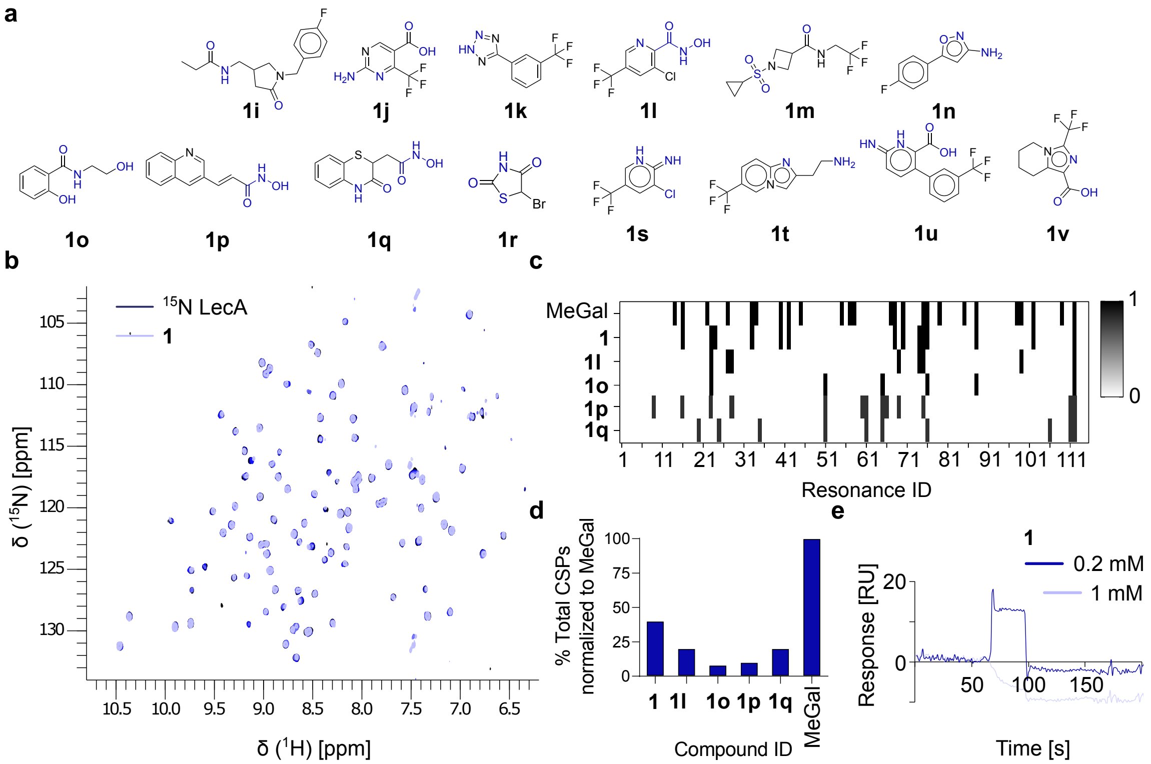

Figure S2 *MBP-like fragments identified for LecA.*
(**a**) Example structures of best hits identified for LecA in ^{19}F (**1i-n, 1s-v**) and TROSY NMR (**1o-1q**) screenings of the general and MBP libraries. The metal-binding scaffold is highlighted in *blue*. Hydroxamates (**1o-q**) are the most potent binders compared to ^{19}F hits (**1i-n**). (**b**) Shown is the fingerprint of ^{15}N LecA in TROSY NMR in the presence of a hydroxamate 0.25–2 mM **1**. (**c**) Shown is a quantitative analysis of perturbed ^{15}N LecA resonances upon addition of 2 mM hydroxamates **1**, **1l**, **1o-q** and 1 mM MeGal in form of a 1:0 plot. The changes in a chemical shift perturbation (CSP) or peak intensity are set to 1. **1** promoted the strongest effect on ^{15}N LecA perturbing resonances similarly to MeGal. (**d**) The plot shows a total % of CSPs normalized to MeGal in ^{15}N LecA upon addition of hydroxamates, where **1** promoted 40% of CSPs in ^{15}N LecA similarly to MeGal. (**e**) Shown is a dose-dependent binding of **1** to LecA in SPR.

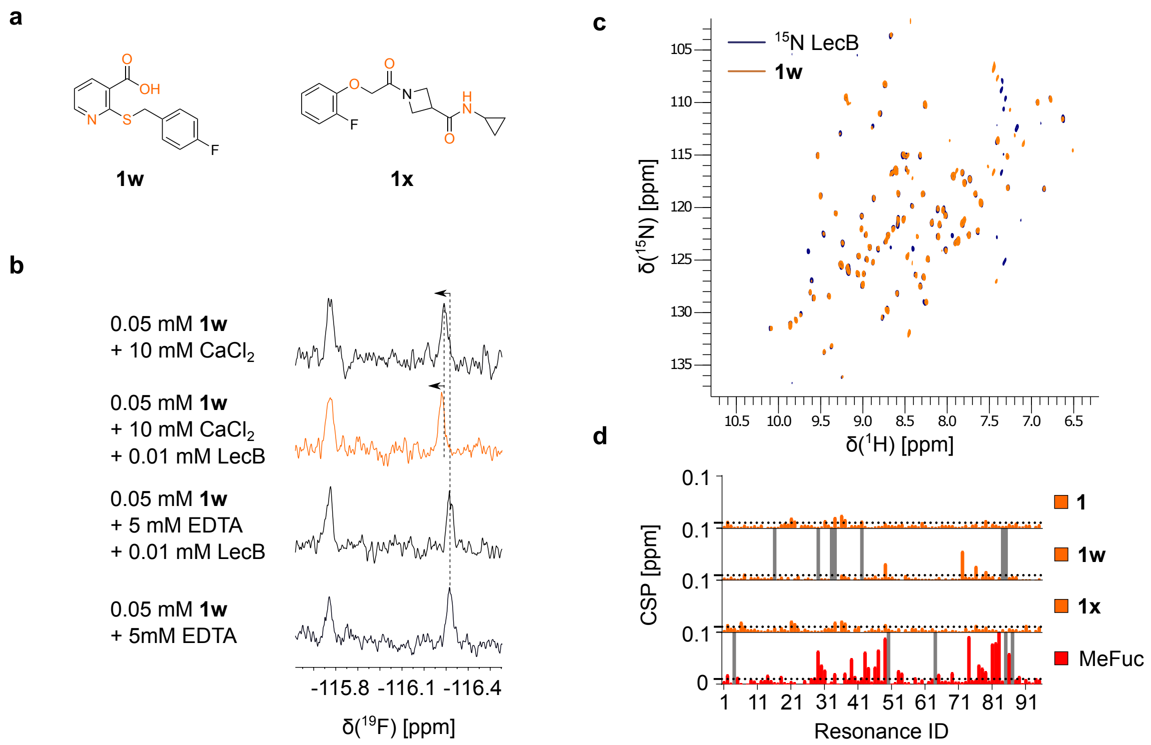

Figure S3 *MBP-like fragments identified for LecB.*
(**a**) Shown are example structures of 2 out of 24 hits identified for LecB in ^{19}F NMR (**1w-x**) screening of the general library. The metal-binding scaffold is highlighted in *orange*. (**b**) ^{19}F NMR spectra of a fragment mixture containing 0.05 mM **1w** and EDTA. Given the chemical shift perturbation of ^{19}F resonance, **1w** bound LecB only in the presence of 10 mM $CaCl_2$ (*orange*). (**c**) Fingerprint of ^{15}N LecB in TROSY NMR alone (*blue*) and in the presence of 2 mM **1w**. (**d**) Shown is a quantitative analysis of perturbed ^{15}N LecB resonances (CSPs>0.01 ppm, *dashed line*) upon addition of 2 mM **1**, **1w**, **1x** and 1 mM MeFuc. The decreased peak intensities are highlighted (*gray*). The compound **1w** perturbed some resonances similarly to MeFuc. Notably, hydroxamate **1** did not bind to ^{15}N LecB.

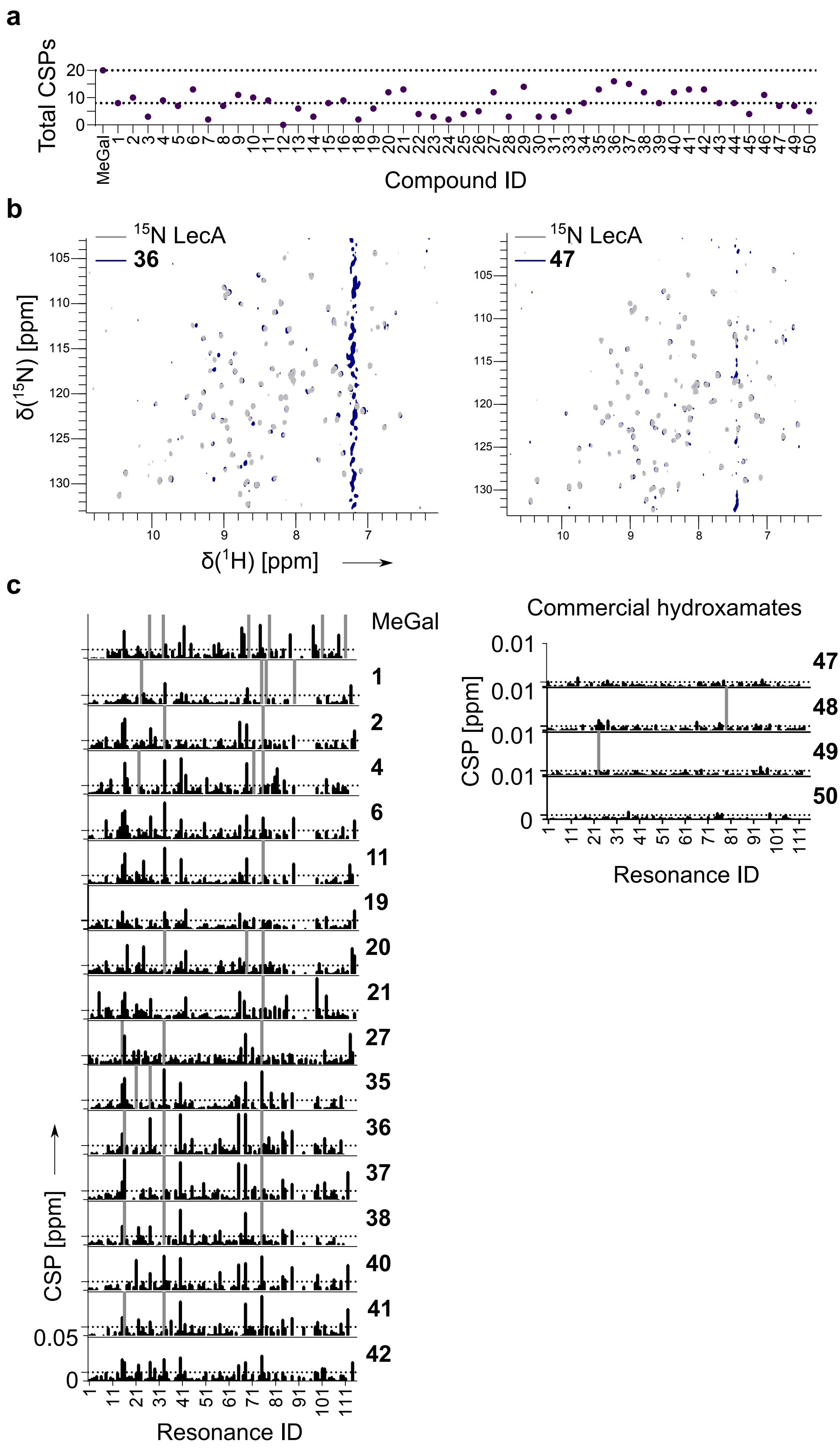
a
Total CSPs
Compound ID
b
15N LecA
36
47
δ(15N) [ppm]
δ(1H) [ppm]
c
Commercial hydroxamates
CSP [ppm]
Resonance ID

Figure S4 *Ranking hydroxamate derivatives in TROSY NMR.*

(**a**) The plot shows a total number of chemical shifts perturbed (CSPs) in ^{15}N LecA upon binding to 4 mM hydroxamate derivatives. To rank the analogs of **1**, we derived the total number of CSPs upon addition of compounds or MeGal. The initial hit **1** was set as the lower limit. Further, compounds that demonstrated binding in this range were subjected to FP assay and PrOF NMR. (**b**) Shown are TROSY NMR spectra of ^{15}N LecA in the presence of 4 mM **36** and **47**. The compound **36** is a derivative of **35** demonstrated a slightly improved binding to ^{15}N LecA compared to **35**. Notably, no binding of the marketed drugs such as **47** has been observed. (**c**) Quantitative analysis of chemical shifts perturbed upon addition of the positive control 1 mM MeGal, 4 mM analogs of **1** or 1 mM marketed hydroxamates (**47-50**). A lower concentration of the marketed drugs has been used due to its high MW and thus, poor solubility at the concentrations above 1 mM. Notably, **35** and its derivatives (**36-40**) promoted the largest changes compared to the initial hit **1**, whereas the marketed hydroxamates did not interact with ^{15}N LecA.

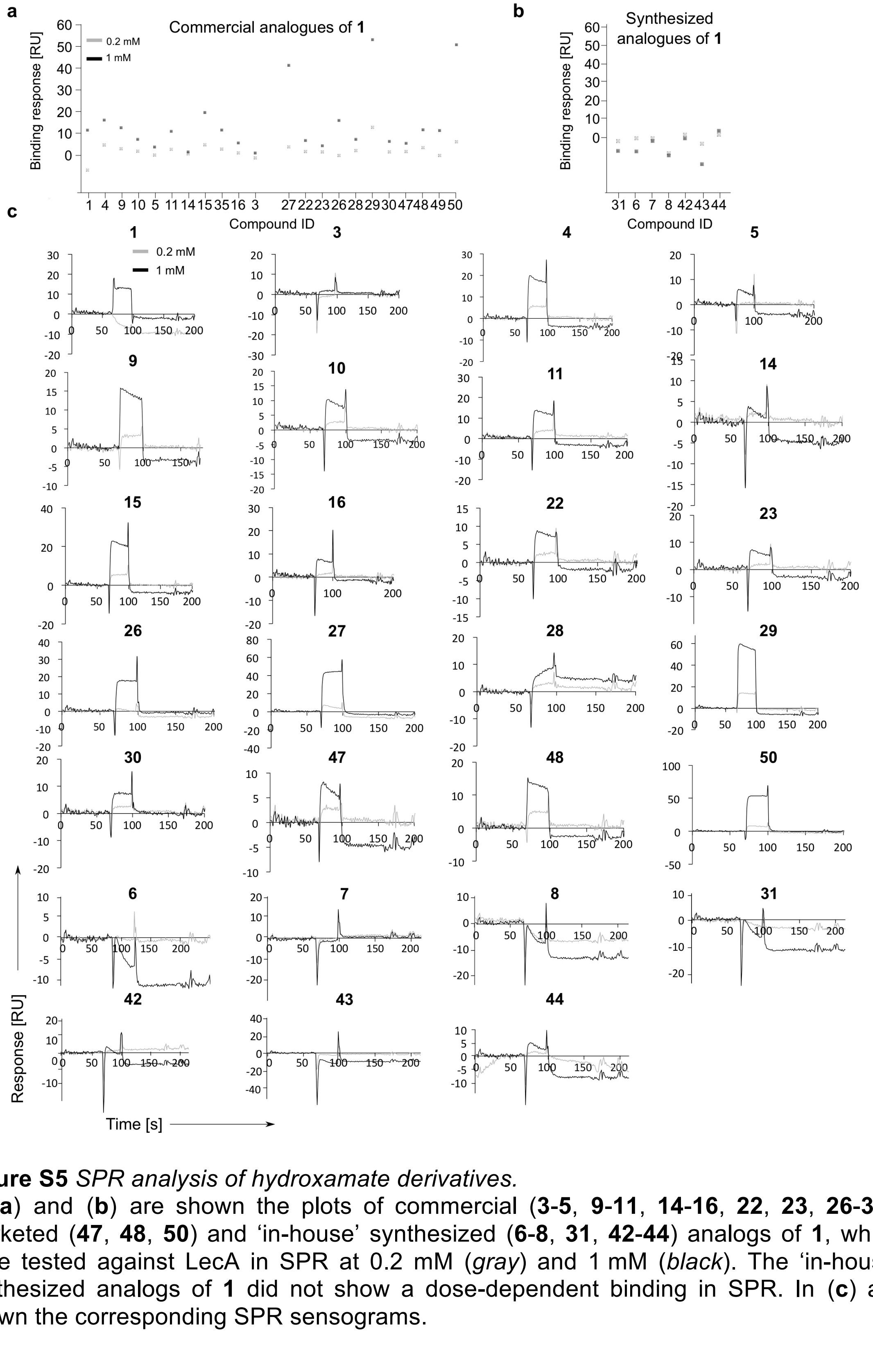

Figure S5 *SPR analysis of hydroxamate derivatives.*
In (**a**) and (**b**) are shown the plots of commercial (**3-5**, **9-11**, **14-16**, **22**, **23**, **26-30**), marketed (**47**, **48**, **50**) and 'in-house' synthesized (**6-8**, **31**, **42-44**) analogs of **1**, which were tested against LecA in SPR at 0.2 mM (*gray*) and 1 mM (*black*). The 'in-house' synthesized analogs of **1** did not show a dose-dependent binding in SPR. In (**c**) are shown the corresponding SPR sensograms.

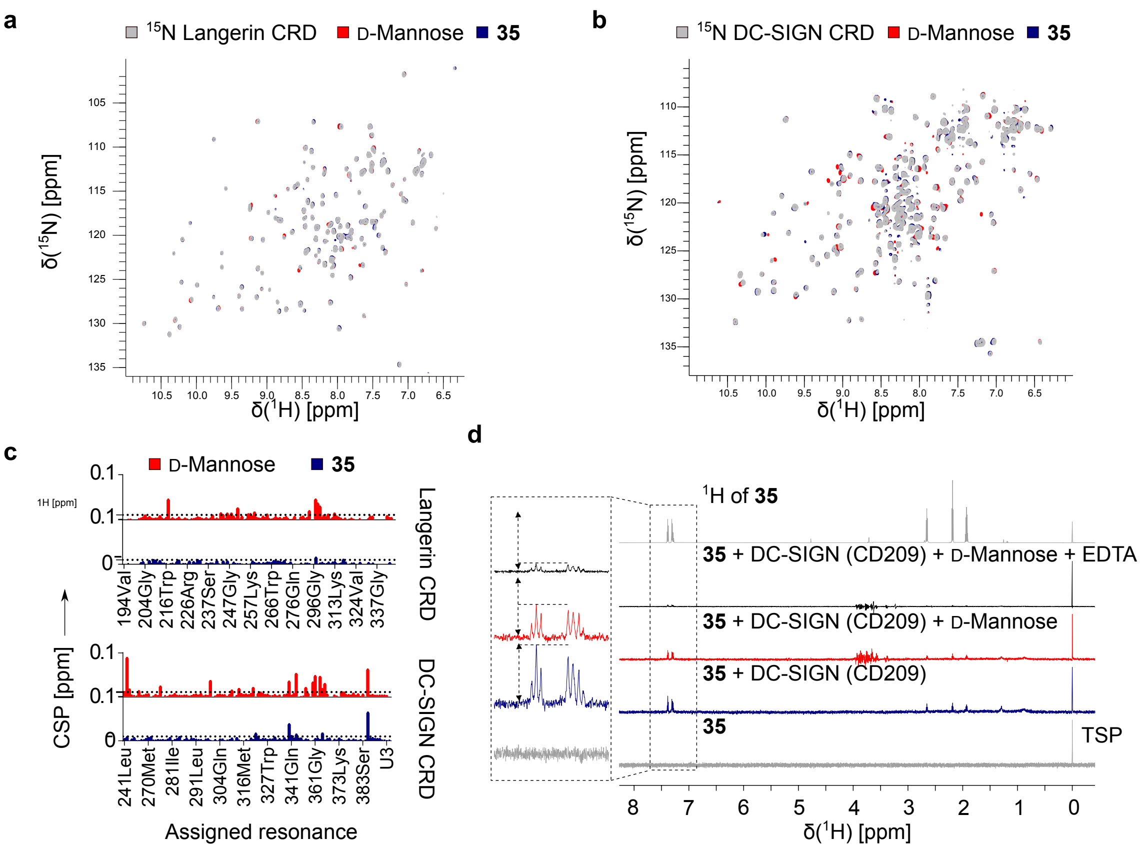

Figure S6 *Investigation of the interactions between* ***35*** *and DC-SIGN or Langerin.* (**a**) and (**b**) are ^{1}H-^{15}N HSQC NMR spectra of ^{15}N-labeled DC-SIGN CRD and Langerin CRD (*gray*), respectively. Next, are shown ^{15}N fingerprints of both lectins with 3 mM **35** (*blue*) and 5 mM D-mannose (*red*). (**c**) Quantitative analysis of chemical shift perturbations (CSPs) in the proteins in the presence of **35** and D-mannose. The small CSPs above 0.01 ppm (*dashed line*) were observed for DC-SIGN CRD, but not Langerin CRD. (**d**) The ^{1}H and STD NMR spectra of 0.5 mM **35** (*gray*) are shown on top and bottom, respectively. The STD NMR spectrum of **35** with 0.02 mM DC-SIGN ECD demonstrated **35** binding given the recovery of ^{1}H resonances in the STD spectrum. Following addition of 100 mM D-mannose a partial competition of the ligand has been observed. This supports hydroxamate binding to a secondary binding site of DC-SIGN ECD rather than to the carbohydrate-binding site. The addition of 10 mM EDTA almost displaced **35** verifying it.

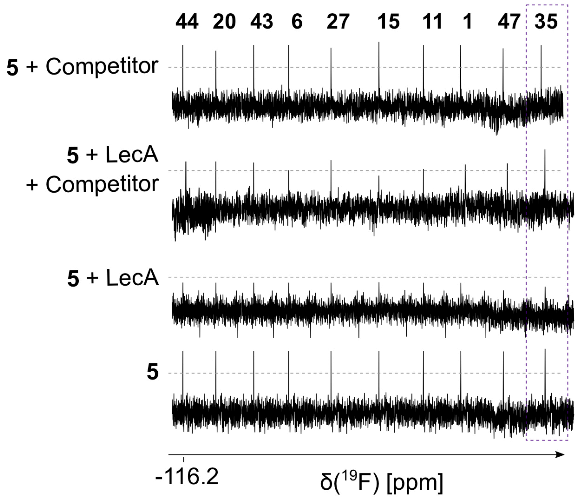

Figure S7 *Competitive* ^{19}F *T_2-filtered NMR study using hydroxamate derivative* ***5*** *as a reporter.*

Shown are the ^{19}F T_2-filtered NMR spectra of 0.1 mM **5** spy molecule in the presence of 10 mM $CaCl_2$ and upon the addition of 0.02 mM LecA. The reduction in peak intensity demonstrated the interaction of **5** with LecA, whereas the addition of 3 mM **35** displaced **5** from the binding site resulting in a recovery of ^{19}F resonance (*violet dashed line*). Notably, the initial hit **1** and the marketed hydroxamate derivative (SAHA, **47**) did not displace **5**.

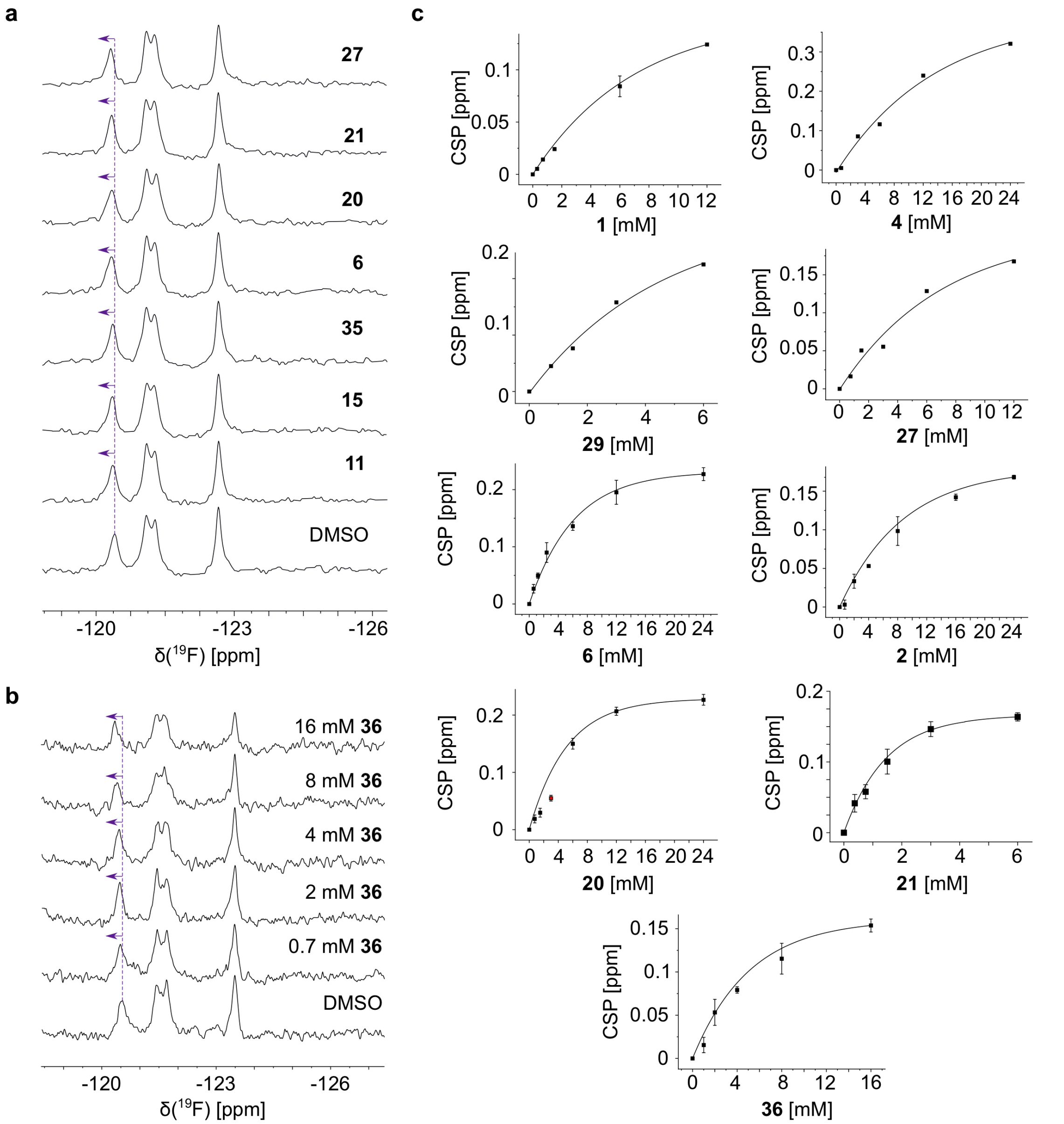

Figure S8 *PrOF NMR of hydroxamate derivatives.*

(**a**) Shown are PrOF NMR spectra of 0.15 mM 5FW-labeled LecA, where the addition of 2 mM hydroxamate derivatives promoted a chemical shift perturbation (CSP) of W42. (**b**) Changes in the chemical shift of W42 were used to derive the affinities (K_d) of hydroxamates for 5FW LecA in PrOF NMR, as shown on example of **36**. (**c**) Titration data using CSPs of W42 were fitted to the one-site binding model to derive the K_d values of the hydroxamate derivatives. The error bars are showing the mean (**1**, **2**, **20**: *n=2*; **6, 21, 36**: *n=3*).

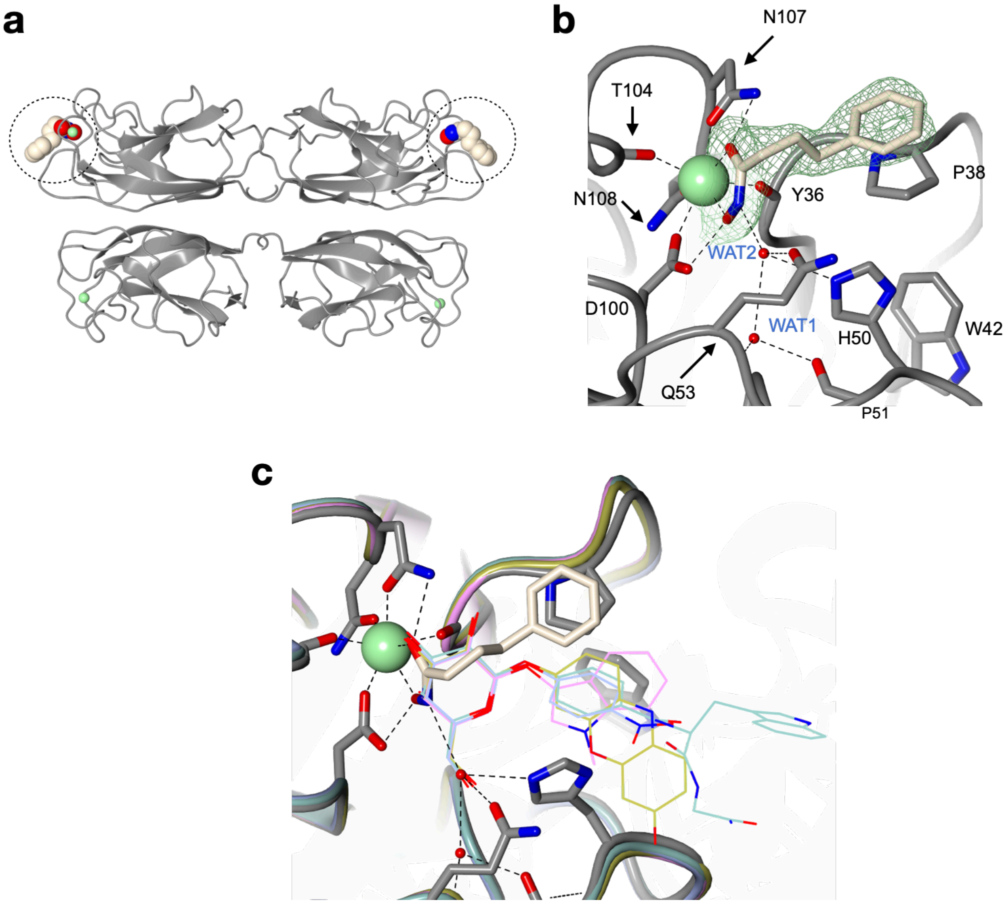

Figure S9 *Crystal structure of LecA in complex with* ***35****.*
(**a**) Overall structure of LecA tetramer is shown in ribbon. Compound 35 is shown in sphere. (**b**) The binding site of **35** in LecA. Electron density corresponding to **35** is shown in green mesh at 1σ. (**c**) Superposition of LecA-**35** with other LecA structures in complex with previously reported monovalent glycomimetics. PDB structures are shown as followed: 4ljh (*pink*), 4lk7 (*gold*), 4lke (*green*) and 3yzf (*blue*).

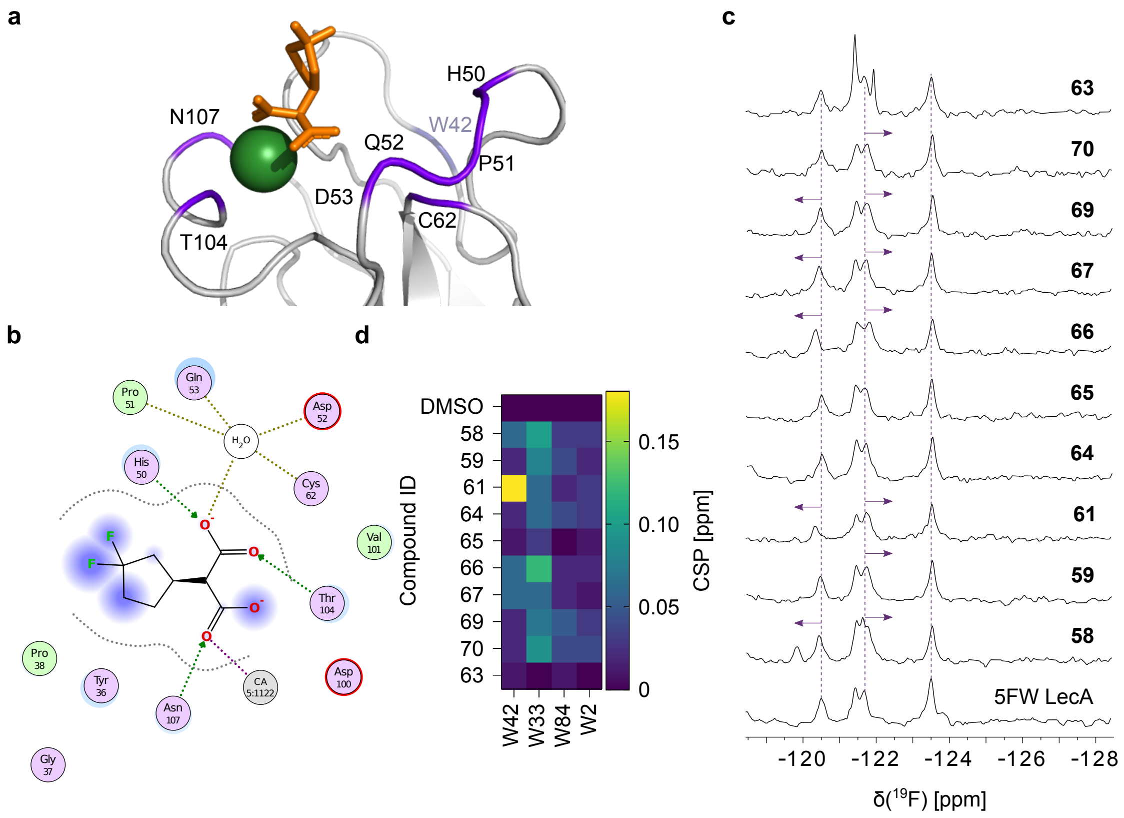

Figure S10 *LecA interacts with* ***58*** *malonate derivatives.*
Shown are (**a**) a docking pose and (**b**) the interaction map of **58** interacting with the orthosteric site of LecA. The residues of the binding site coordinating **58** are highlighted (*violet*). The carbohydrate-binding site residues are shown as follows: polar (*pink*), hydrophobic (*green*) and acidic (*red contour ring*). Shown are the contacts of **58** to Ca^{2+} (*pink line*), hydrogen bonds to side-chains (*green arrow*, pose 1: H50, T104 and N107) and to a water molecule (*yellow*). The solvent exposed surface area of **58** atoms is shown as a gradient of dark-to-light blue 'clouds' meaning more and less solvent exposure, respectively. Light-blue 'corona' around the residues indicates the degree of interaction with **58**, i.e. larger and darker is more interaction. (**c**) One-point-titration experiments in PrOF NMR using 0.15 mM 5FW LecA and 2 mM malonic acid derivatives shows the compounds that perturbed W42 and W33 and LecA similarly to **58**. DMSO and MeGal served as negative and positive controls, respectively. (**d**) Heat map shows the magnitude of CSP of the 5FW resonances in the presence of **58** analogs, where malonates **58**, **61**, **66** and **67** perturbed W42.

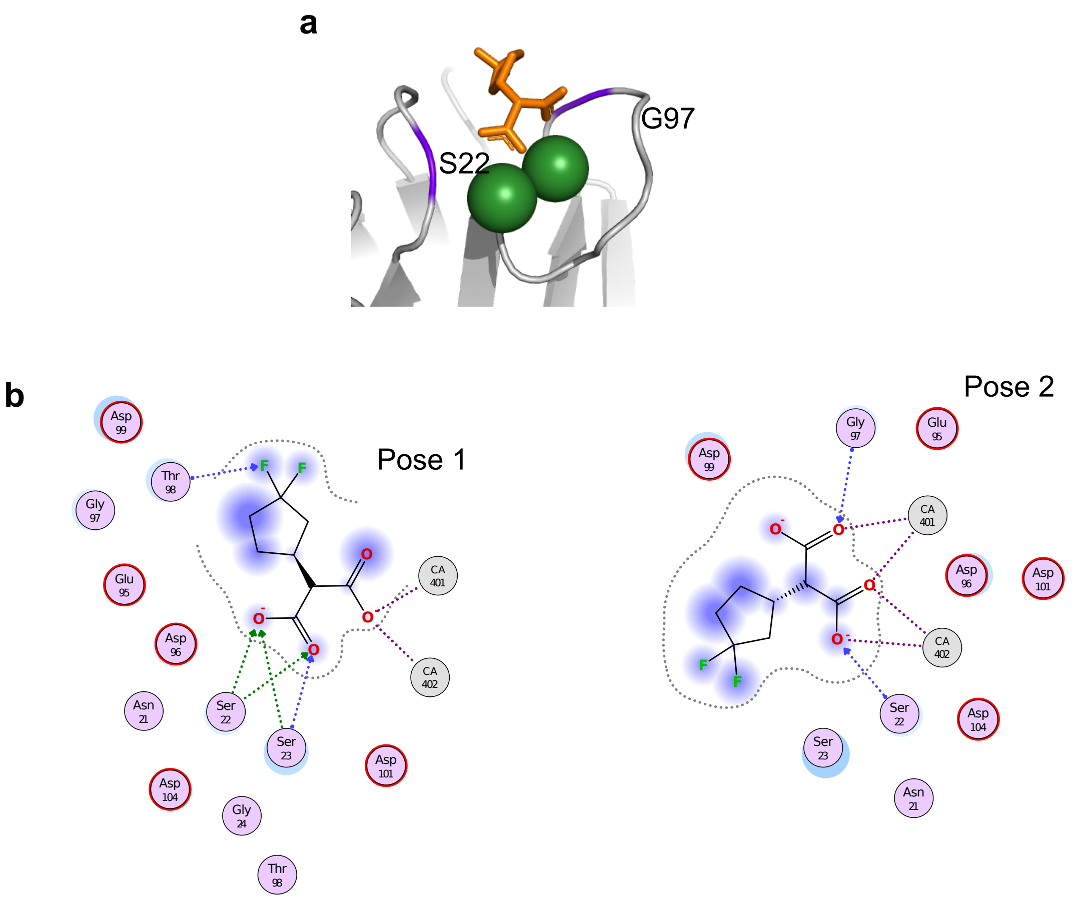

Figure S11 *Docking study of LecB with **58**.*
(**a**) Shown is the docking pose 2 of **58** to the orthosteric site of LecB. The Ca^{2+} ions are shown in *green* and the interacting residues are in *violet*. (**b**) Interaction maps of poses 1 and 2 of **58** and LecB. The residues of the carbohydrate-binding site are shown as follows: polar (*pink*), hydrophobic (*green*) and acidic (*red contour ring*). Additionally, shown are the contacts of **58** to Ca^{2+} (*pink line*), hydrogen bonds to sidechains (*green arrow*, pose 1: S22 and S23) and backbone atoms (*blue arrow*, pose 1: S23. T98 and pose 2: S22 and G97). The solvent exposed surface area of **58** atoms is shown as a gradient of darker and larger blue 'clouds' meaning less and more solvent exposure, respectively. Light-blue "corona" around the residues S23, D96 and D99 indicated the degree of interaction with **58** (larger and darker indicates more interaction). Pose 1 suggested a potential interaction of CF_2-group with the protein surface through T98.

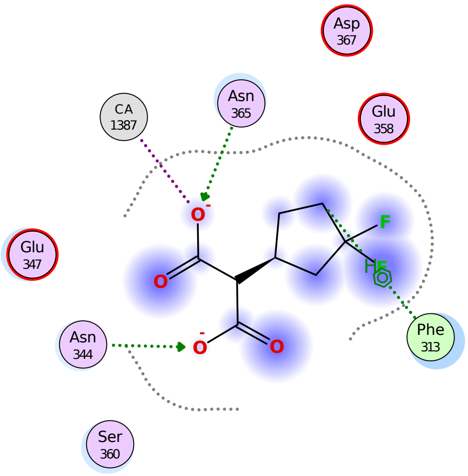

Figure S12 *Interaction map of **58** with DC-SIGN CRD.*
Shown are the carbohydrate-binding site residues of DC-SIGN CRD as follows: polar (*pink*), hydrophobic (*green*) and acidic (*red contour ring*). Shown are the contacts of **58** to Ca^{2+} (*pink line*), hydrogen bonds to side chains (*green arrow*, pose 1: N365 and N344) and a benzene icon next to F313 representing a CH-π staking interaction. The solvent exposed surface area of **58** is shown as a gradient of darker and larger blue 'clouds' meaning more and less solvent exposure, respectively. Light-blue 'corona' around residues indicates the degree of interaction with **58** (larger and darker indicates more interaction).

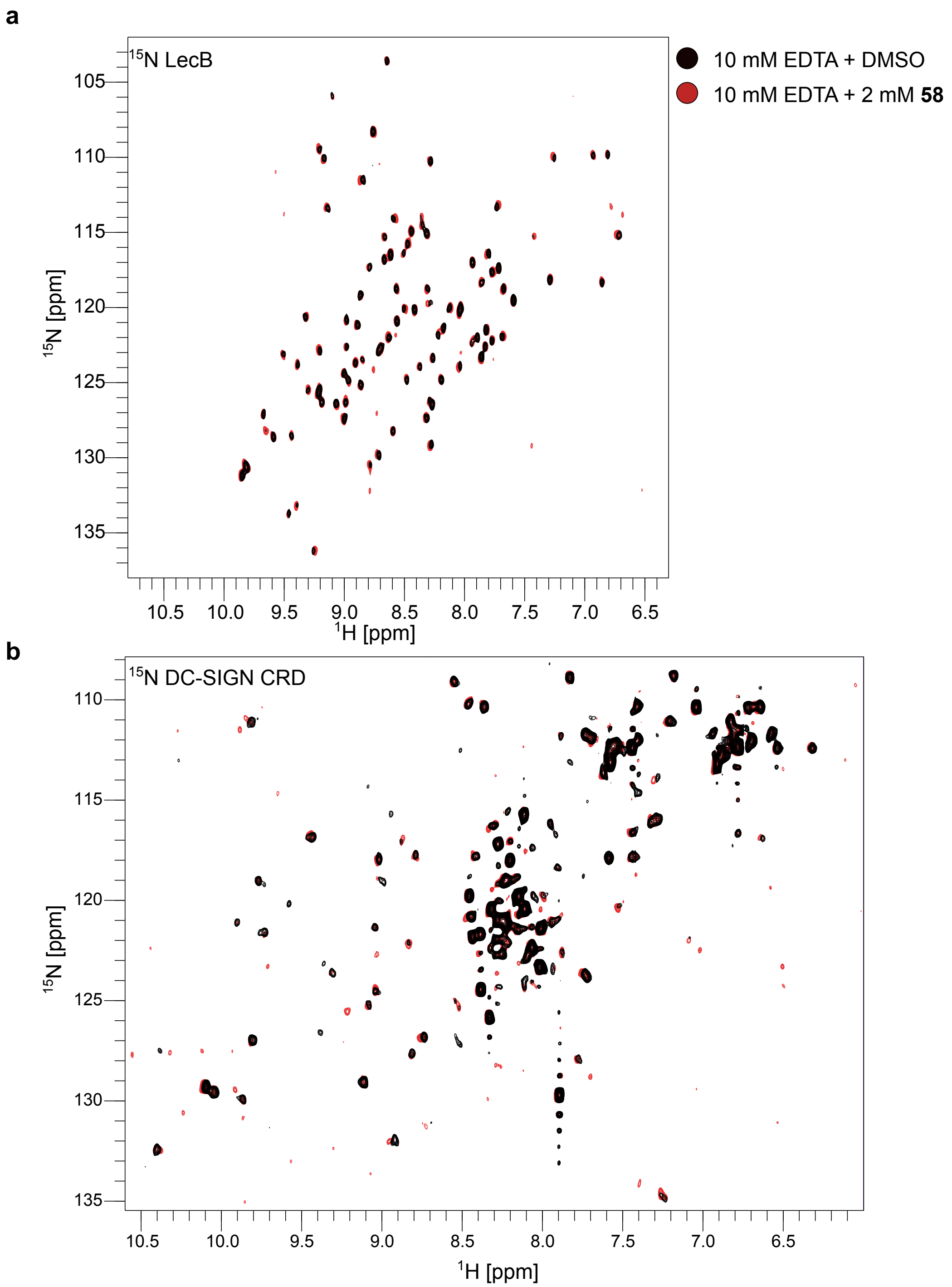

Figure S13 *^{1}H-^{15}N HSQC/TROSY NMR:* ***58*** *binding is Ca^{2+}-dependent.*
Shown are ^{1}H-^{15}N TROSY/HSQC NMR spectra of (**a**) 0.07 mM ^{15}N LecB and (**b**) 0.1 mM ^{15}N DC-SIGN CRD in the presence of 10 mM EDTA with DMSO (*black*) or 2 mM **58** (*red*). Malonate **58** did not bind neither ^{15}N LecB not ^{15}N DC-SIGN in the presence of EDTA demonstrating Ca^{2+}-dependent nature of **58**-lectin interaction.

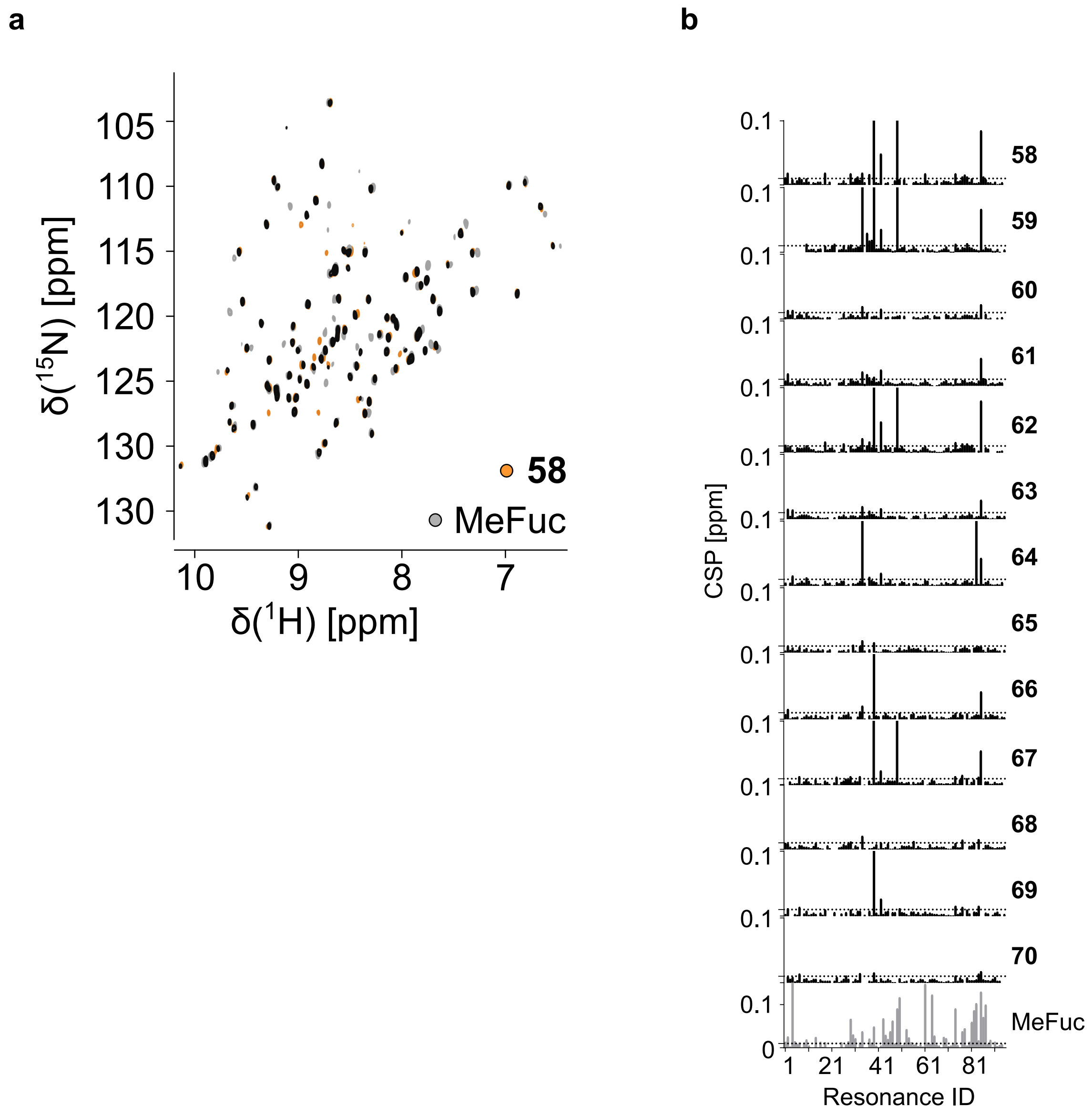

Figure S14 *TROSY NMR of ^{15}N LecB with **58** analogs.*
(**a**) TROSY NMR spectra of ^{15}N LecB fingerprints (*gray*) in the presence of 2 mM **58** (*orange*) or MeFuc (*gray*). (**b**) Quantitative analysis of TROSY NMR shown as the CSP plots of ^{15}N LecB resonance IDs in the presence of 2 mM malonic acid derivatives of **58** and 1 mM MeFuc. MeFuc is a positive control verifying the protein is active. Compounds harboring a malonate group **58**, **59**, **62**, **66** and **67** promoted more CSPs in ^{15}N LecB similar to MeFuc suggesting malonates targeted its orthosteric site. Compared to **64**, **58** and **59** showed more CSPs in ^{15}N LecB suggesting a role of an electronegative group in the interaction with ^{15}N LecB, which was proposed in the docking study.

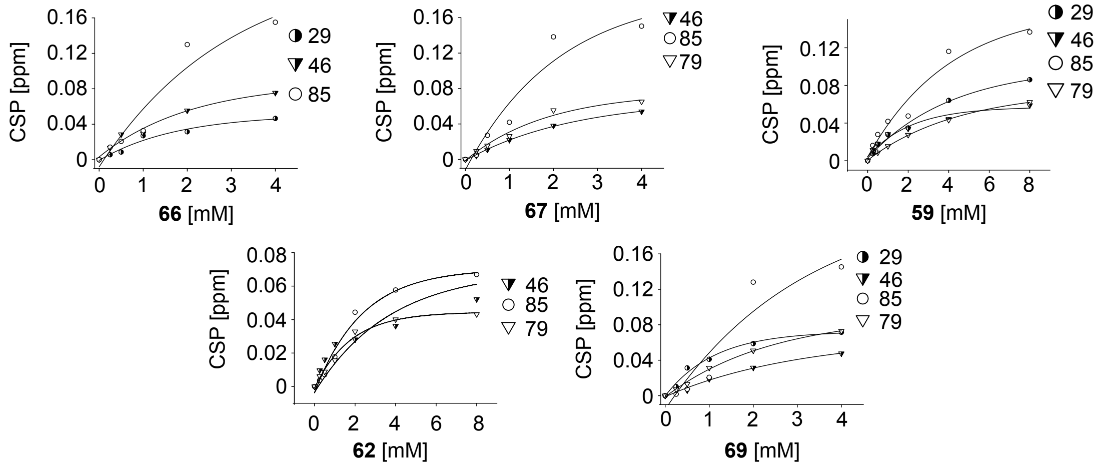

Figure S15 *Titration TROSY NMR studies of **58** derivatives and LecB.*
Titration data using chemical shift perturbations (CSPs) of resonance IDs perturbed in the presence of malonic acid derivatives were fitted to the one-site binding model to derive K_d values.

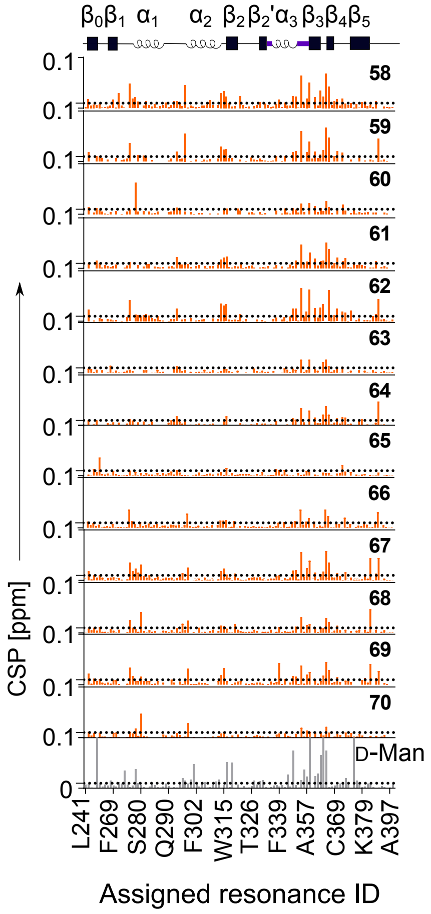

Figure S16 *Interaction study of DC-SIGN CRD with malonates.*
Shown are the chemical shift perturbations (CSPs) of 0.1 mM ^{15}N DC-SIGN CRD resonances in ^{1}H-^{15}N HSQC NMR in the presence of 2 mM malonic acid derivatives or D-mannose as a positive control. Compounds harboring a malonate group **58**, **59**, **62**

and **67** perturbed the most resonances in ^{15}N DC-SIGN. Notably, malonates promoted the CSPs in a similar manner to D-mannose suggesting these compounds targeted the orthosteric site of ^{15}N DC-SIGN CRD.

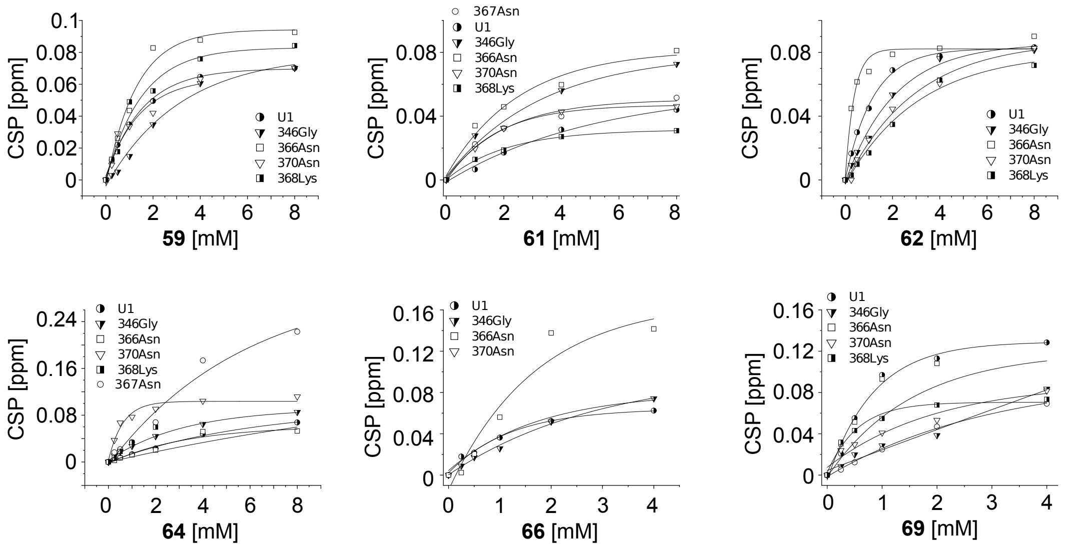

Figure S17 *^{1}H-^{15}N HSQC NMR titration study for malonate derivatives with ^{15}N DC-SIGN CRD.*

Titration data using chemical shift perturbations (CSPs) of resonances perturbed in the presence of **58** derivatives were fitted to the one-site binding model to derive K_d values. The assigned resonances of DC-SIGN CRD are shown directly in graphs, whereas U1 was an unassigned resonance.

Supplementary Tables

Table S1 *List of LecA PDB structures and co-crystalized ligands analyzed for virtual screening.*

PDB ID	Resolution (Å)
1l71	1.5
1uoj	2.4
1oko	1.6
2vxj	1.9
2wyf	2.4
3zyb	2.29
3zyf	1.94
3zyh	1.5
4a6s	2.15
4al9	1.75
4cp9	1.65
4cpb	1.57
4ljh	1.45
4lk6	2.86
4lk7	1.76

4lkd	2.31
4lke	1.65
4lkf	1.64
4yw6	1.4
4yw7	1.82
4ywa	1.19
5d21	1.9
5mih	1.8

Table S2 *List of LecB PDB structures and co-crystalized ligands analyzed for virtual screening.*

PDB-ID	Resolution (Å)
1ous	1.2
1oux	2.0
5a6q	1.7
1gzt	1.3
1our	1.42
1ovp	1.4
1ovs	1.75
1oxc	1.2
1uzv	1.0
1w8f	1.05
1w8h	1.75
2boj	1.8
2bp6	2.5
2jdh	1.1
2jdk	1.1
2jdm	1.7
2jdn	1.3
2jdp	1.3
2jdu	1.5
2jdy	1.7
2vuc	1.3
2vud	1.7
3dcq	1.8
3zdv	1.41
4ce8	0.9
5a3o	1.6
5a6x	1.55
5a6y	1.4
5a6z	1.5
5a70	1.6
5d2a	2.13
5hch	2.9
5i8m	2.13
5i8x	1.89
5nes	1.61
5ney	1.55
5nf0	1.27

5ngq	1.17
5may	1.65
5maz	1.45
5mb1	1.65

Table S3 *Commercial and synthesized hydroxamates.*
Shown are the structures of hydroxamates and its affinity and LE values derived in PrOF NMR, [%] inhibition in competitive fluorescence polarization (FP) assay and [%] efficiency in SPR towards LecA. Group: 1) commercial and synthesized derivatives of the hydroxamate **1**, 2) diversity oriented, 3) derivatives of the hydroxamate **35** and 4) marketed drugs for metalloenzymes.

ID	Compound	PA-IL (LecA)			
		K_d in PrOF NMR [mM]	LE [kcal mol^{-1} HA^{-1}]	Inhibition in FP [%]	Efficiency in SPR [%]
	Group 1				
**1**	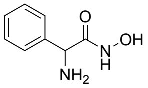	7.2 ± 1.4	0.25	6 *	11.6
**2**	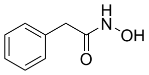	6.1 ± 0.9	0.29	18 ± 1	*n.d.*
**3**	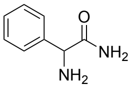	*n.d.*	*n.d.*	*No inhibition*	*No dose response*
**4**	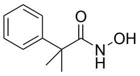	9.4 ± 2.5	0.22	10%	14.8
**5**	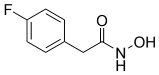	*n.d.*	*n.d.*	*n.d.*	4.1
**6**	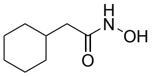	4.4 ± 0.6	0.30	21 ± 1	*n.d.*
**7**	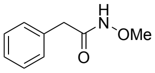	*n.d.*	*n.d.*	*No inhibiton*	*n.d.*
**8**	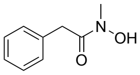	*n.d.*	*n.d.*	20 ± 3	*n.d.*
**9**	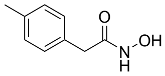	*n.d.*	*n.d.*	16 ± 2	12.8
**10**	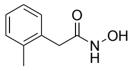	*n.d.*	*n.d.*	14 ± 4	7.7
**11**	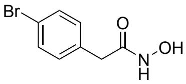	*n.d.*	*n.d.*	14 ± 1	14
**12**	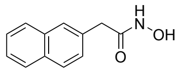	*n.d.*	*n.d.*	*n.d.*	*n.d.*
**13**	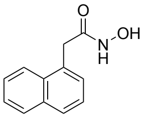	*n.d.*	*n.d.*	*n.d.*	*n.d.*
**14**	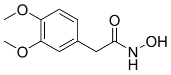	*n.d.*	*n.d.*	*n.d.*	3.5

**15**	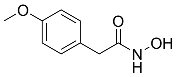	*n.d.*	*n.d.*	16 ± 4	10.2
**16**	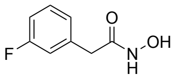	*n.d.*	*n.d.*	*n.d.*	6
	Group 2				
**17**	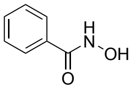	*n.d.*	*n.d.*	*n.d.*	14.2
**18**	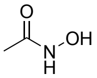	*n.d.*	*n.d.*	*n.d.*	*n.d.*
**19**	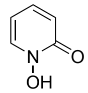	*n.d*	*n.d*	37 ± 2	*n.d.*
**20**	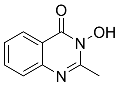	4.5 ± 0.2	0.26	33	*n.d.*
**21**	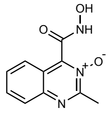	2.4 ± 0.4	0.23	*n.d.*	*n.d.*
**22**	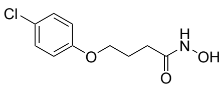	*n.d.*	*n.d.*	*n.d.*	5.2
**23**	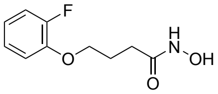	*n.d.*	*n.d.*	*n.d.*	3.4
**24**	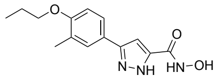	*n.d.*	*n.d.*	*n.d.*	*n.d.*
**25**	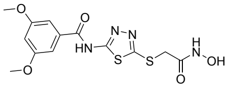	*n.d.*	*n.d.*	*n.d.*	3.1
**26**	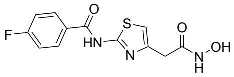	*Precipitated at 2 mM*	*n.d.*	*n.d.*	9.2
**27**	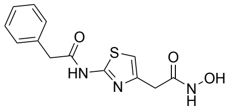	4.8 ± 1.3	0.17	15	23.1
**28**	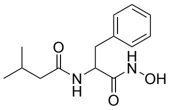	*n.d.*	*n.d.*	*n.d.*	4.9
**29**	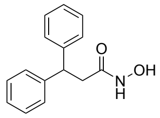	3.1 ± 0.9	0.20	*n.d.*	36.1
**30**	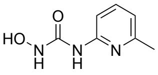	*n.d.*	*n.d.*	*n.d.*	6.8
**31**	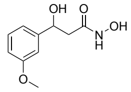	*n.d.*	*n.d.*	9 ± 2	*n.d.*

**32**	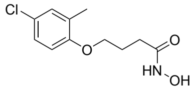	*n.d.*	*n.d.*	5 ± 3	*n.d.*
**33**	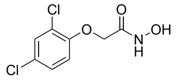	*n.d.*	*n.d.*	*Precipitated at 10 mM*	*n.d.*
**34**	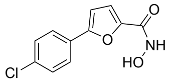	*n.d.*	*n.d.*	*Precipitated at 10 mM*	*n.d.*
	Group 3				
**35**	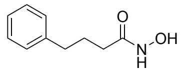	4.6 ± 0.9	0.26	26 ± 1	10.9
**36**	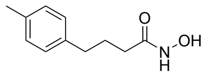	3.6 ± 2.2	0.25	35 ± 3	*n.d.*
**37**	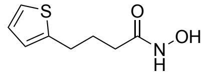	*n.d.*	*n.d.*	25 ± 3	*n.d.*
**38**	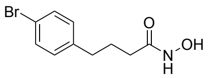	*n.d.*	*n.d.*	25 ± 1	*n.d.*
**39**	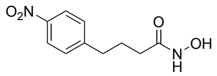	*n.d.*	*n.d.*	4 ± 1	*n.d.*
**40**	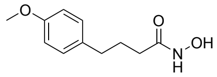	*n.d.*	*n.d.*	27 ± 1	*n.d.*
**41**	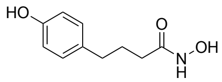	*n.d.*	*n.d.*	39 ± 1	*n.d.*
**42**	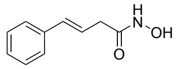	*n.d.*	*n.d.*	13 ± 3	*n.d.*
**43**	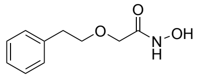	*n.d.*	*n.d.*	8 ± 1	*n.d.*
**44**	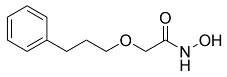	*n.d.*	*n.d.*	17 ± 2	*n.d.*
**45**	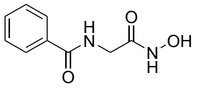	*n.d.*	*n.d.*	*No inhibiton*	*n.d.*
**46**	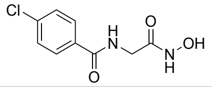	*n.d.*	*n.d.*	*n.d.*	*n.d.*
	Group 4				
**47**	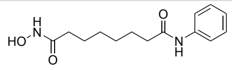	*n.d.*	*n.d.*	*n.d.*	3.8
**48**	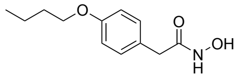	*Precipitated at 1 mM*	*n.d.*	*n.d.*	9.0
**49**	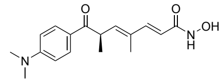	*n.d.*	*n.d.*	*n.d.*	27.7
**50**	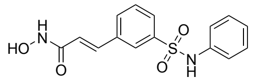	*n.d.*	*n.d.*	*n.d.*	7.5

Inhibition [%] compared to MeGal at 10 mM (16 h). * Measured at 4 mM
n.d. = not determined due to poor binding of compounds in TROSY NMR or FP assay

Table S4 *Quantitative analysis of PrOF NMR.*

ID	W42 [ppm]	W33 [ppm]	W2 [ppm]	W84 [ppm]
**DMSO**	120.50	121.67	121.43	123.50
**58**	120.44	121.77	121.47	123.53
**59**	120.48	121.75	121.46	123.54
**60**	*n.d.*	*n.d.*	*n.d.*	*n.d.*
**61**	120.32	121.73	121.46	123.52
**62**	*n.d.*	*n.d.*	*n.d.*	*n.d.*
**63**	120.49	121.67	121.44	123.51
**64**	120.52	121.73	121.47	123.54
**65**	120.51	121.70	121.44	123.50
**66**	120.43	121.80	121.46	123.52
**67**	120.44	121.74	121.44	123.52
**68**	120.48	121.74	121.47	123.55
**69**	120.51	121.76	121.48	123.54
**70**	120.50	121.67	121.43	123.50

n.d. = not determined

Table S5 *Statistics for data collection and refinement of LecA-***35** *complex*.

**PDB ID**	**7FJH**
**Data collection**	
Beamline	PROXIMA-2 (SOLEIL)
Wavelength (Å)	0.979
Detector	EIGER X 9M
Resolution (Å)	46.85-1.79 (1.83-1.79)
Space group	$P2_12_12_1$
a, b, c (Å)	48.98, 51.68, 160.53
α, β, γ (°)	90.0, 90.0, 90.0
Total observations	332865
Unique reflections	39017
Multiplicity	8.5 (7.5)
Mean $I/\sigma(I)^a$	14.2 (2.2)
Completeness (%)[a]	99.4 (89.9)
$R_{merge}^{a,b}$	0.073 (0.717)
$CC_{1/2}^{a,c}$	0.999 (0.949)
Refinement	
Reflections: working/free[d]	38945/2019
R_{work}/R_{free}^{e}	0.180/0.220
Ramachandran plot:	
allowed/favoured/outliers (%)	3/97/0
r.m.s bond deviations (Å)	0.0113
r.m.s angle deviations (°)	1.560
Mean *B*-factors: protein/ligand[f]/water (Å^2)	35/39/38

[a] Values for the outer resolution shell are given in parentheses.
[b] Rmerge = $\sum_{hkl} \sum_i |I_i(hkl) - \langle I(hkl)\rangle| / \sum_{hkl} \sum_i I_i(hkl)$.
[c] $CC_{½}$ is the correlation coefficient between symmetry-related intensities taken from random halves of the dataset.
[d] The data set was split into "working" and "free" sets consisting of 95 and 5% of the data, respectively. The free set was not used for refinement.

[e] The R-factors R_{work} and R_{free} are calculated as follows: $R = \sum(| F_{obs} - F_{calc} |)/\sum| F_{obs} |$, where F_{obs} and F_{calc} are the observed and calculated structure factor amplitudes, respectively

Table S6 *Commercial derivatives of malonic acid **58**.*
Shown are the structures of malonates, its affinities and LE values for DC-SIGN CRD and LecB derived in ^{1}H-^{15}N HSQC and TROSY NMR, respectively.

ID	Compound	DC-SIGN CRD (CD209)		PA-IIL (LecB)	
		K_d [mM] in HSQC NMR	LE [kcal mol^{-1} HA^{-1}]	K_d [mM] in TROSY NMR	LE [kcal mol^{-1} HA^{-1}]
**58**	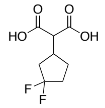	1.2 ± 0.5	0.28	1.2 ± 04	0.29
**59**	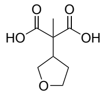	1.2 ± 0.4	0.31	2.7 ± 0.6	0.28
**60**	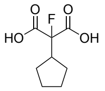	*n.d.*	*n.d.*	*n.d.*	*n.d.*
**61**	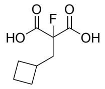	1.9 ± 0.8	0.28	*n.d.*	*n.d.*
**62**	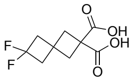	1.5 ± 0.7	0.26	1.5 ± 0.2	0.27
**63**	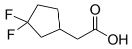	*n.d.*	*n.d.*	*n.d.*	*n.d.*
**64**	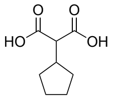	2.6 ± 0.9	0.29	2.6 ± 0.6	0.31
**65**	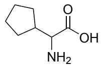	*n.d.*	*n.d.*	*n.d.*	*n.d.*
**66**	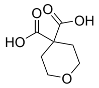	1.3 ± 0.4	0.33	1.6 ± 0.4	0.33
**67**	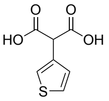	1.2 ± 0.5	0.33	1.6 ± 0.3	0.31
**68**	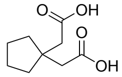	*n.d.*	*n.d.*	*n.d.*	*n.d.*
**69**	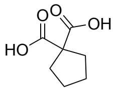	1.0 ± 0.6	0.37	1.7 ± 0.6	0.36
**70**	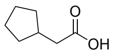	*n.d.*	*n.d.*	*n.d.*	*n.d.*

n.d. = not determined due to poor binding of compounds in TROSY or HSQC NMR

Table S7 *List of assigned resonances in ^{15}N DC-SIGN CRD.*

Assigned resonance ID	^{1}H [ppm]	^{15}N [ppm]
241Leu	7.86	123.52
242Val	7.10	121.48
260Trp	8.43	120.81

261Thr	9.79	119.27
262Phe	9.13	129.22
263Phe	8.82	127.47
264Gln	8.76	127.15
266Asn	7.79	118.38
267Cys	9.40	116.79
268Tyr	9.91	121.05
269Phe	8.54	124.96
270Met	7.79	128.09
271Ser	7.77	120.49
273Ser	7.35	113.65
274Gln	8.32	116.32
275Arg	9.72	123.15
276Asn	9.46	119.69
277Trp	8.22	122.07
278His	7.44	114.82
279Asp	9.03	118.97
280Ser	8.11	124.48
281Ile	7.07	122.05
282Thr	7.34	116.19
283Ala	7.79	123.60
284Cys	7.77	113.18
285Lys	8.27	124.36
286Glu	8.22	118.87
287Val	7.18	108.85
288Gly	7.83	108.91
289Ala	8.32	122.35
290Gln	8.05	117.44
291Leu	8.93	132.33
292Val	7.86	122.73
293Val	7.02	126.95
295Lys	9.05	122.43
296Ser	7.95	116.33
298Glu	9.00	117.46
299Glu	8.01	122.45
300Gln	7.82	121.63
301Asn	8.20	117.05
302Phe	7.47	119.76
304Gln	8.91	122.02
305Leu	7.38	117.77
306Gln	7.20	115.79
307Ser	7.44	113.37
308Ser	8.59	117.80
310Ser	7.12	110.97
311Asn	8.00	118.59
312Arg	7.52	116.49
313Phe	8.54	127.65
315Trp	10.02	127.32
316Met	8.46	113.23
317Gly	9.74	111.28
318Leu	8.27	128.26

319Ser	8.45	118.78
320Asp	7.86	123.58
321Leu	6.65	119.47
322Asn	7.93	115.42
323Gln	7.70	120.63
324Glu	8.06	128.40
326Thr	8.55	121.22
327Trp	8.83	129.05
328Gln	9.08	123.37
329Trp	9.47	128.87
330Val	8.92	115.00
331Asp	7.58	117.81
332Gly	8.57	109.13
333Ser	8.29	119.38
335Leu	7.83	120.43
336Leu	8.76	132.12
339Phe	8.33	120.52
340Lys	7.48	118.84
341Gln	7.30	113.96
342Tyr	6.69	118.67
343Trp	6.47	117.55
344Asn	10.06	123.48
346Gly	8.01	121.43
347Glu	8.16	118.88
352Gly	8.33	112.55
356Cys	8.06	116.52
357Ala	7.99	125.80
358Glu	9.08	116.63
360Ser	9.03	116.35
361Gly	8.97	116.57
363Gly	7.58	113.28
364Trp	8.51	122.38
365Asn	9.13	117.80
366Asp	9.08	121.51
367Asp	9.91	125.47
368Lys	9.61	120.43
369Cys	8.46	119.89
370Asn	7.87	111.75
371Leu	8.07	124.17
372Ala	8.26	122.78
373Lys	8.55	123.68
374Phe	7.27	117.80
375Trp	8.91	117.05
376Ile	6.57	116.99
377Cys	9.03	121.38
378Lys	9.27	123.66
379Lys	9.07	125.34
380Ser	8.77	117.67
381Ala	7.66	123.70
382Ala	9.03	124.56
383Ser	7.74	112.53

386Arg	8.43	121.81
388Glu	8.28	120.03
390Gln	8.15	119.85
392Leu	8.02	123.43
393Ser	8.20	118.01
397Ala	8.38	124.45
398Thr	8.11	115.71

Table S8 *List of resonance IDs in ^{15}N LecB.*

Resonance ID	^{1}H [ppm]	^{15}N [ppm]
1	9.24	136.17
2	9.45	133.97
3	9.37	133.16
4	10.10	131.56
5	9.86	131.19
6	9.79	130.74
7	9.73	130.21
8	8.70	129.77
9	8.25	129.06
10	9.58	128.71
11	9.40	128.35
12	8.60	128.21
13	9.62	128.16
14	8.32	127.51
15	9.00	127.39
16	9.61	126.92
17	8.28	126.58
18	8.36	126.35
19	9.05	126.32
20	8.98	126.33
21	9.16	126.10
22	9.17	125.74
23	9.26	125.40
24	8.88	125.23
25	8.22	124.83
26	8.95	124.92
27	8.46	124.69
28	9.05	124.57
29	9.64	124.16
30	8.02	124.04
31	8.81	123.93
32	8.91	123.78
33	8.40	123.86
34	8.67	123.96
35	9.24	123.39
36	7.88	123.46
37	8.74	123.30
38	7.87	123.28
39	8.36	122.80
40	8.11	122.75

41	8.70	122.64
42	7.93	122.64
43	7.74	122.67
44	9.46	122.48
45	8.97	122.65
46	7.63	122.26
47	9.02	122.05
48	8.63	122.00
49	8.59	121.67
50	8.10	121.66
51	7.81	121.50
52	8.18	121.40
53	8.51	121.09
54	7.79	121.22
55	8.58	121.10
56	9.01	120.79
57	8.01	120.72
58	9.32	120.58
59	8.05	120.19
60	8.11	120.14
61	8.41	119.94
62	7.60	119.64
63	8.87	119.09
64	9.50	118.90
65	8.57	118.68
66	8.28	118.72
67	7.66	118.72
68	6.85	118.31
69	7.28	118.18
70	7.78	117.66
71	7.72	117.28
72	7.93	117.01
73	8.65	116.74
74	7.82	116.60
75	8.61	116.45
76	8.48	116.31
77	7.28	115.18
78	8.53	114.96
79	8.47	115.13
80	8.32	115.13
81	9.54	115.09
82	7.40	113.64
83	7.96	113.62
84	9.27	112.98
85	8.88	112.25
86	6.62	111.58
87	8.80	111.13
88	9.16	110.09
89	8.26	110.24
90	6.93	109.96
91	6.78	109.72

92	9.20	109.56
93	8.73	108.30
94	8.66	103.62
95	9.25	125.49

Table S9 *List of resonances in ^{15}N LecA.*

Resonance ID	^{1}H [ppm]	^{15}N [ppm]
1	8.66	132.29
2	10.44	131.27
3	8.77	131.12
4	8.53	130.16
5	8.69	129.87
6	8.66	129.72
7	9.88	129.63
8	9.13	129.49
9	9.74	129.41
10	8.30	129.43
11	10.36	128.83
12	7.33	128.87
13	7.10	128.52
14	8.97	128.42
15	8.80	128.18
16	8.62	127.56
17	7.68	127.33
18	8.47	127.06
19	8.65	126.79
20	8.88	126.74
21	9.73	126.75
22	7.35	126.63
23	9.31	125.71
24	9.18	125.52
25	9.75	125.26
26	8.92	125.15
27	9.59	124.75
28	8.18	124.62
29	8.36	124.16
30	6.77	123.75
31	8.95	123.71
32	8.31	123.66
33	8.50	123.16
34	8.16	123.21
35	9.01	123.04
36	8.34	122.98
37	8.97	122.99
38	7.59	122.98
39	8.64	122.80
40	7.42	122.79
41	9.14	122.45
42	6.54	122.36

43	8.67	122.16
44	9.41	122.10
45	7.08	121.93
46	8.13	121.72
47	9.33	121.44
48	8.59	121.36
49	7.15	121.38
50	9.94	121.11
51	8.89	121.03
52	8.21	120.48
53	8.14	120.42
54	9.52	120.32
55	7.40	120.06
56	9.28	119.84
57	7.82	119.77
58	7.77	119.58
59	7.24	119.30
60	9.05	119.14
61	8.48	118.90
62	8.03	118.54
63	8.75	118.52
64	7.39	117.89
65	8.24	117.74
66	8.05	117.72
67	7.89	117.71
68	9.18	117.54
69	8.84	117.49
70	7.48	117.42
71	8.34	117.31
72	6.97	116.86
73	7.73	116.60
74	8.90	116.54
75	9.12	116.20
76	8.84	115.56
77	9.18	115.39
78	7.15	115.03
79	8.42	114.46
80	8.07	114.36
81	9.28	113.86
82	9.04	113.55
83	6.96	112.87
84	8.09	112.85
85	8.43	112.77
86	6.87	112.58
87	7.30	113.51
88	9.42	112.48
89	6.73	112.54
90	6.88	112.39
91	7.30	112.24
92	6.78	112.25
93	7.47	112.00

94	7.19	111.28
95	6.72	111.38
96	7.28	111.04
97	6.62	110.91
98	8.30	110.95
99	8.54	110.59
100	7.55	110.45
101	6.96	110.48
102	8.76	110.05
103	8.21	109.95
104	8.97	109.13
105	8.94	108.67
106	9.01	108.20
107	8.45	107.41
108	7.79	106.90
109	8.50	106.77
110	8.16	104.95
111	6.91	104.43
112	8.38	120.11
113	7.71	122.25
114	6.74	105.68
115	6.78	113.52

Supplementary Schemes

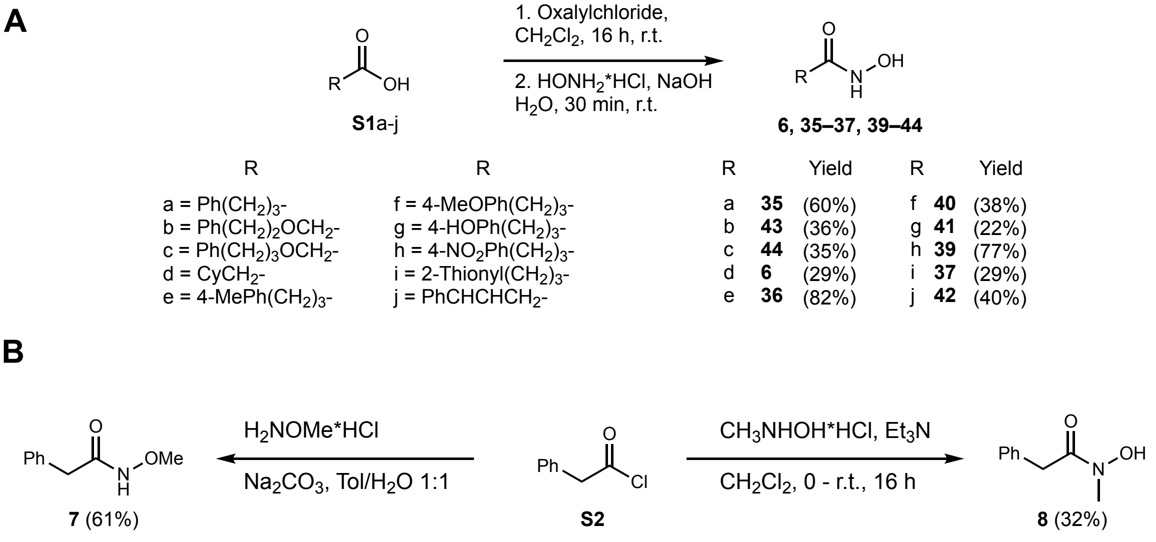

Scheme 1: Synthetic modifications of acids/ acyl chlorides for generating a library of hydroxamic acids: (**A**) Synthesis of hydroxamic acid derivatives. (**B**) Modifications at the hydroxamic acid functional group

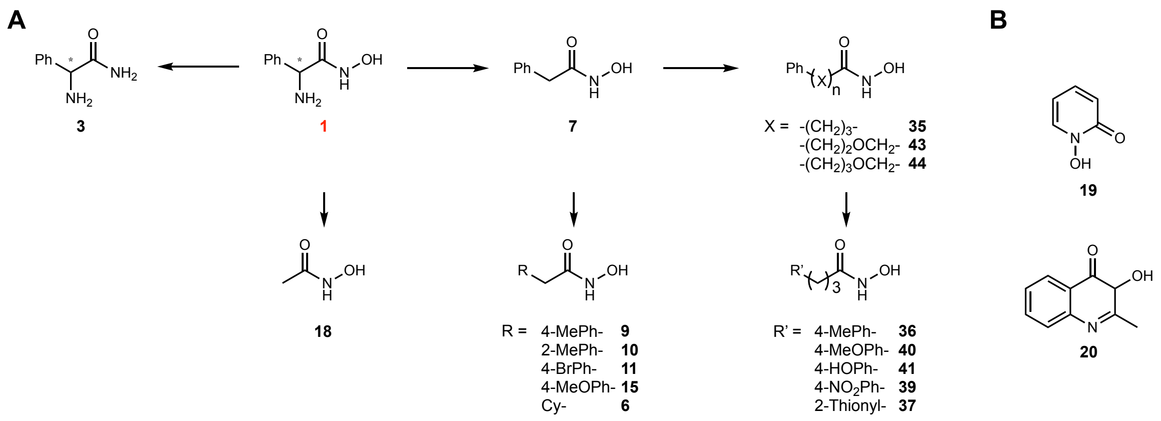

Scheme 2: Structures of the hydroxamic acid library (**A**) Optimization of the initial hit **1** leading to the *N*-hydroxy-4-phenylbutanamide series. (**B**) Structures with cyclic hydroxamic acid functional groups.

Supplementary Note 1

NMR Spectra

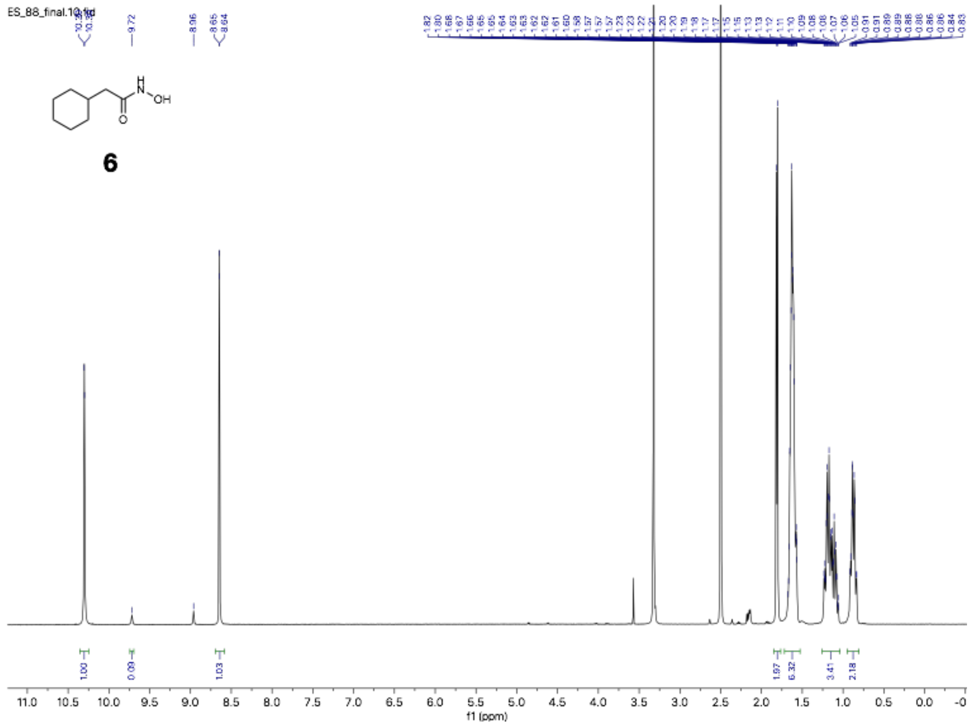

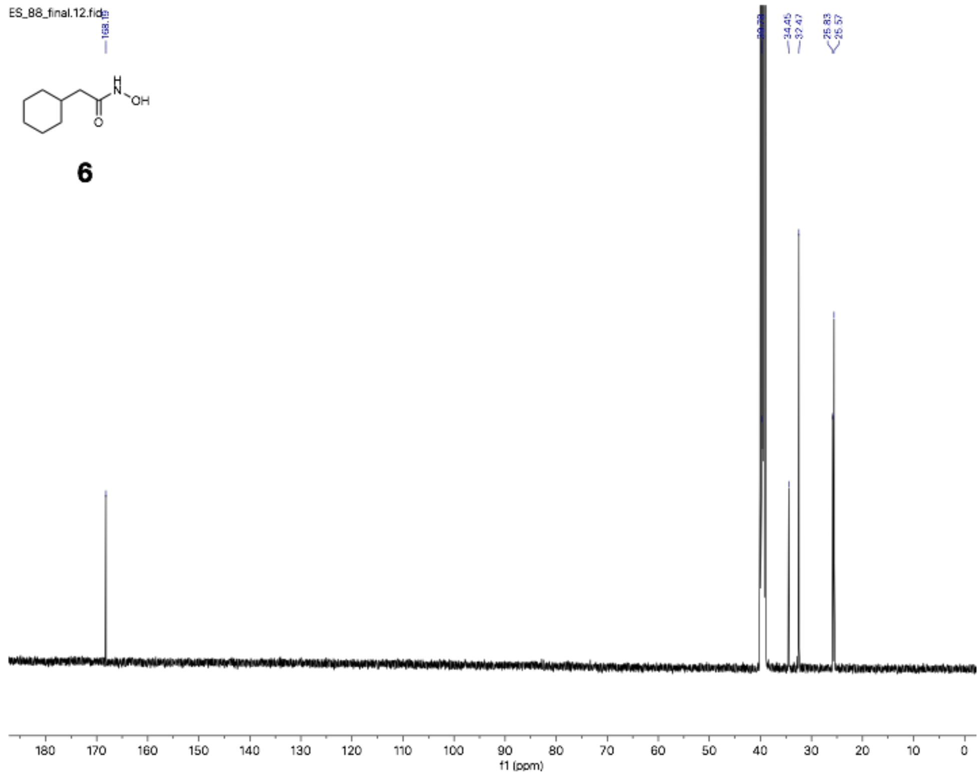

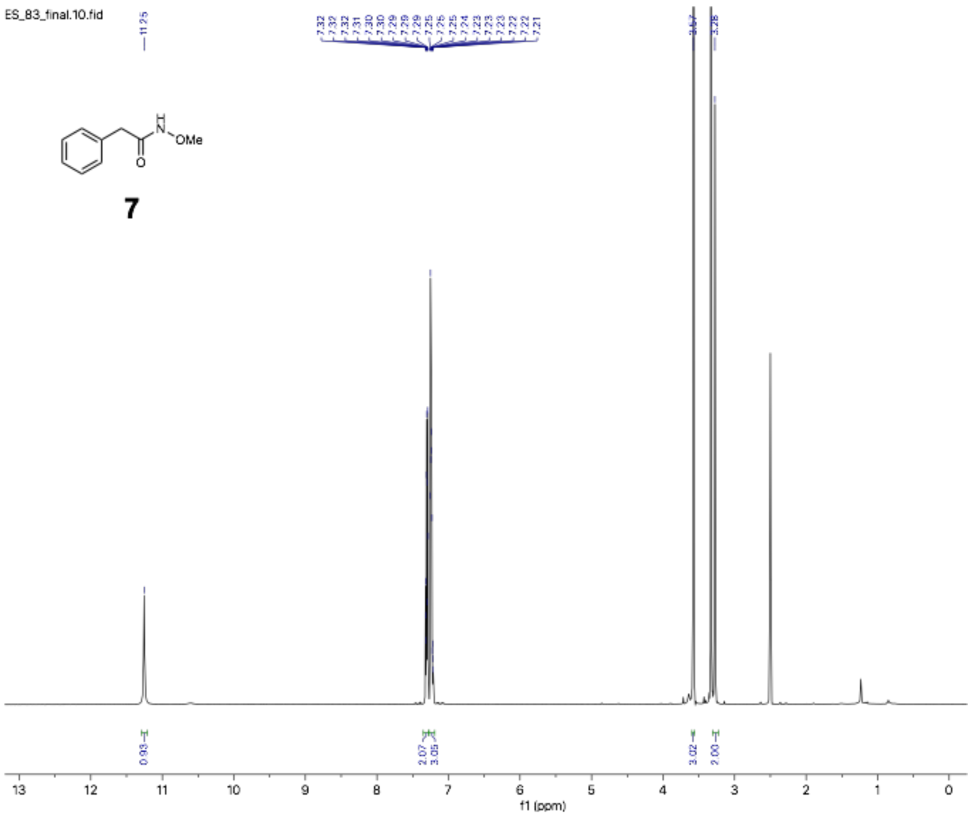
ES_83_final.10.fid
11.25
7.32 7.32 7.32 7.31 7.30 7.30 7.29 7.29 7.29 7.25 7.25 7.25 7.24 7.23 7.23 7.23 7.22 7.22 7.22 7.21
3.57
3.28
OMe
7
0.93
2.07
3.05
3.02
2.00
13 12 11 10 9 8 7 6 5 4 3 2 1 0
f1 (ppm)

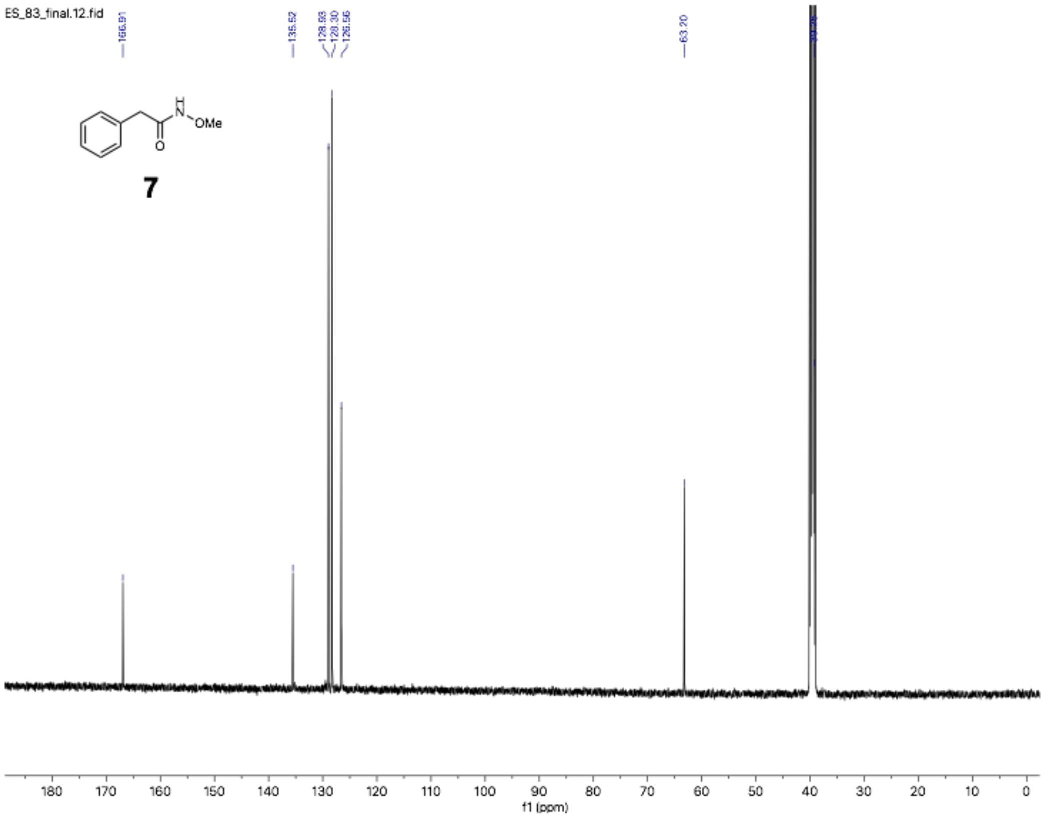
ES_83_final.12.fid
166.91
135.52
128.93
128.30
126.56
63.20
OMe
7
180 170 160 150 140 130 120 110 100 90 80 70 60 50 40 30 20 10 0
f1 (ppm)

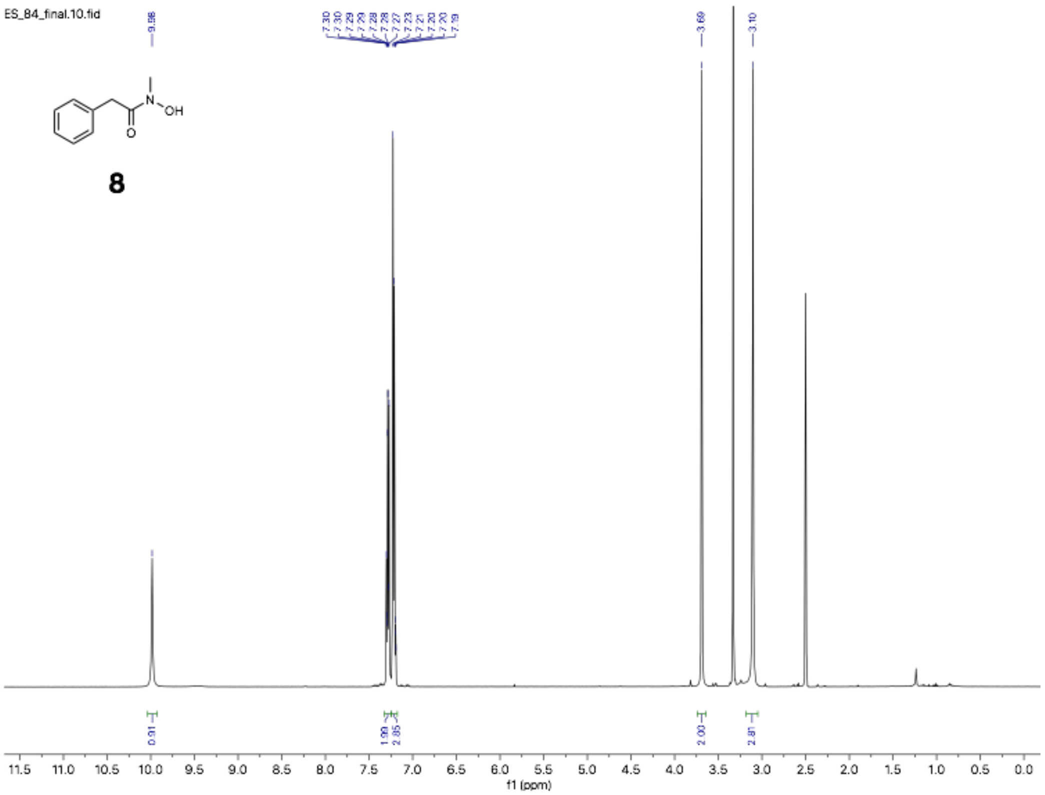
ES_84_final.10.fid
9.98
7.30 7.30 7.29 7.29 7.28 7.28 7.27 7.23 7.21 7.20 7.20 7.19
3.69
3.10
N
OH
O
8
f1 (ppm)

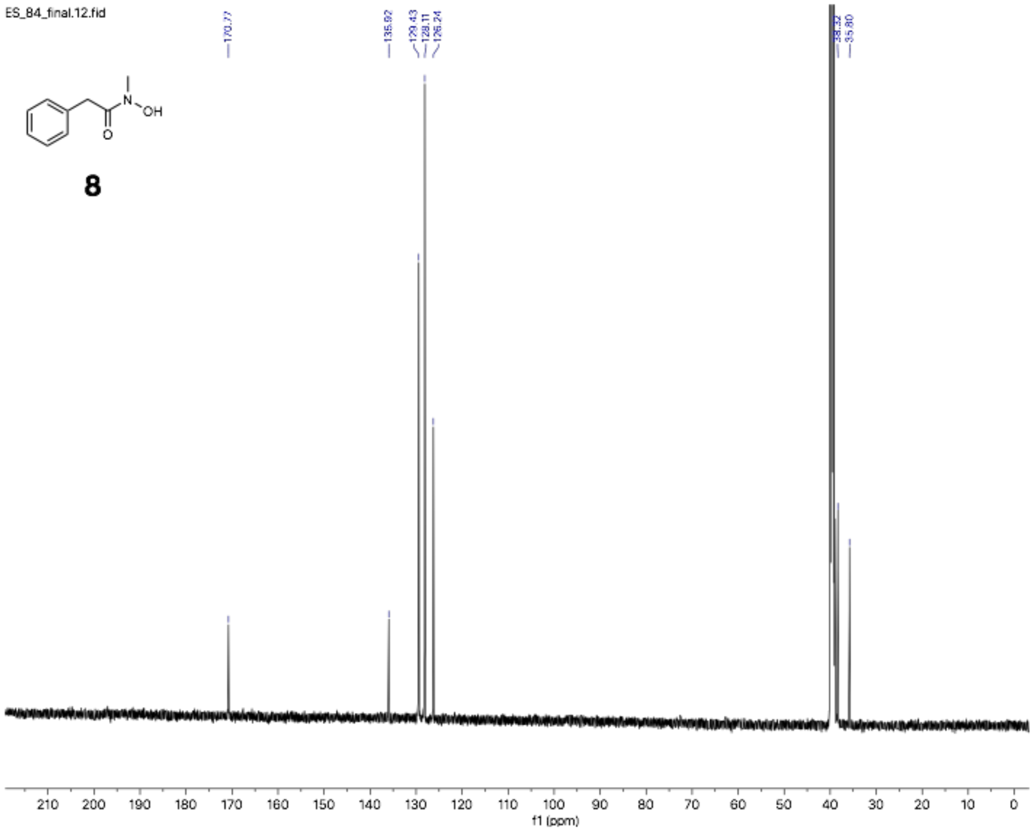
ES_84_final.12.fid
170.77
135.92
129.43
128.11
126.24
38.32
35.80
N
OH
O
8
f1 (ppm)

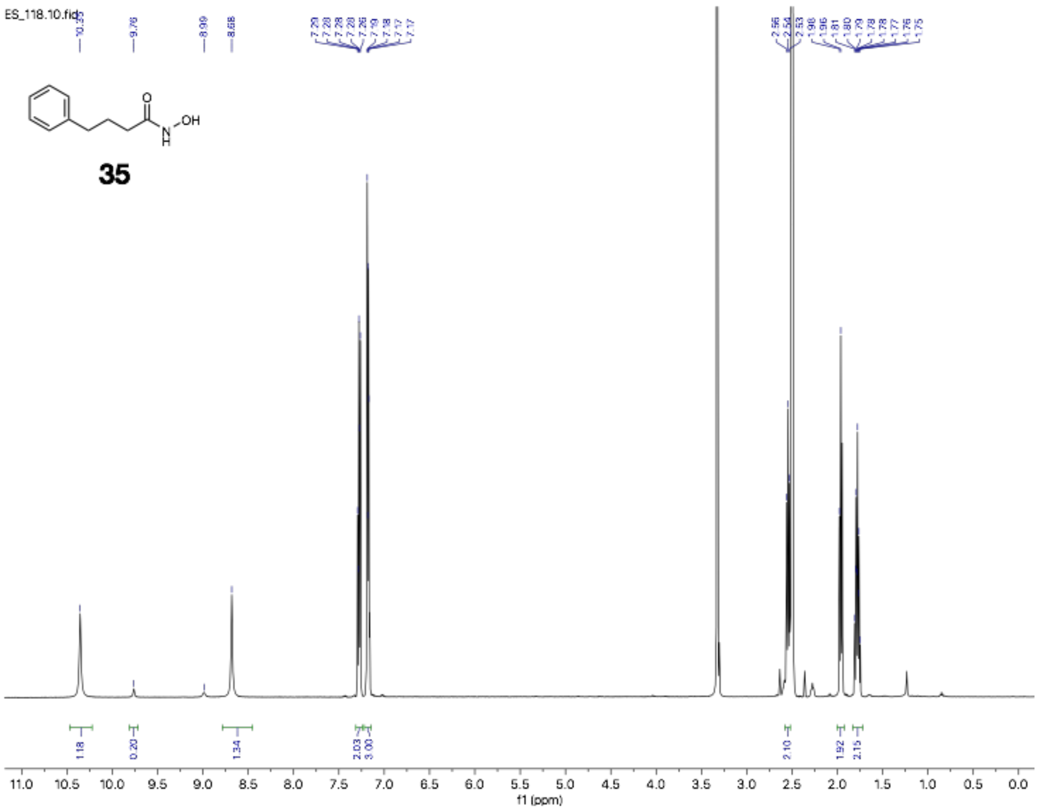
ES_118.10.fid
35
f1 (ppm)

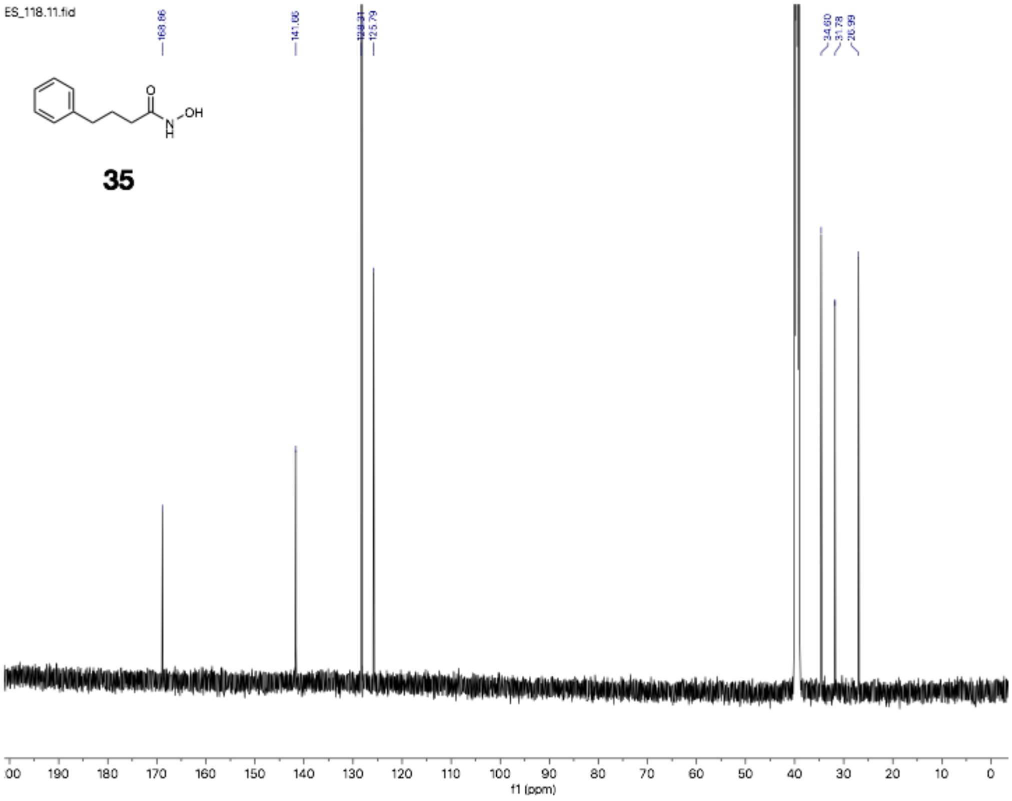
ES_118.11.fid
168.86
141.66
125.79
34.60
31.78
26.99
35
f1 (ppm)

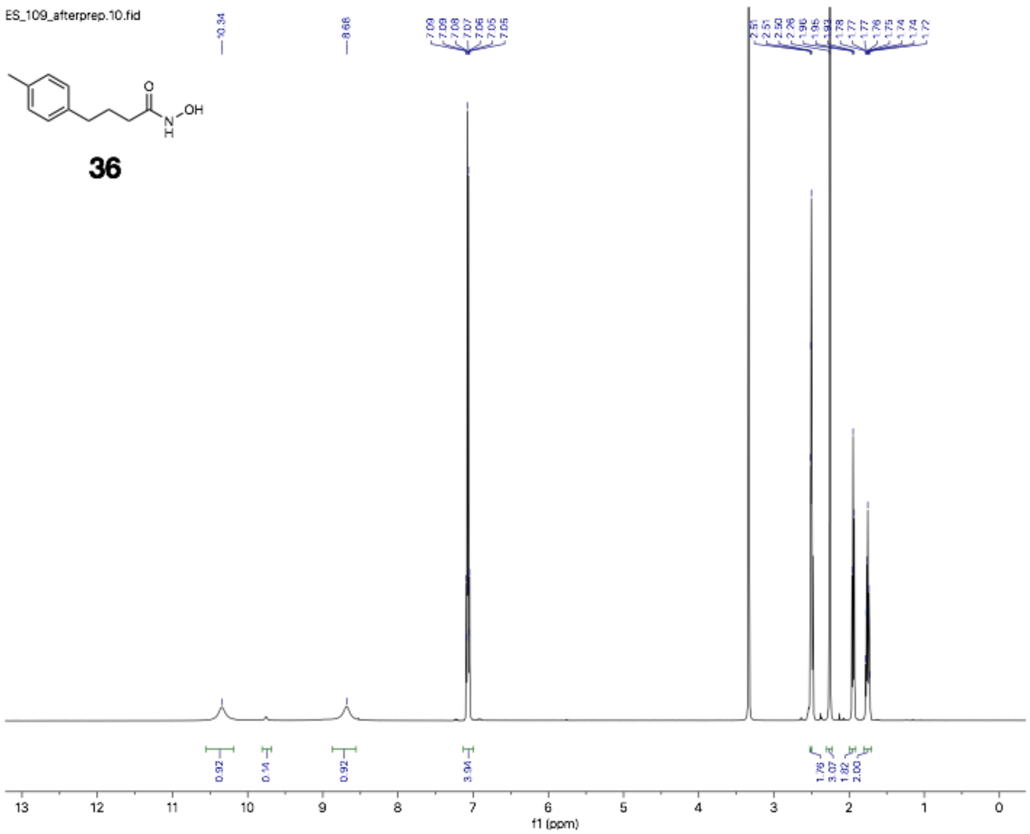
ES_109_afterprep.10.fid
36
10.34
8.68
0.92
0.14
0.92
3.94
f1 (ppm)

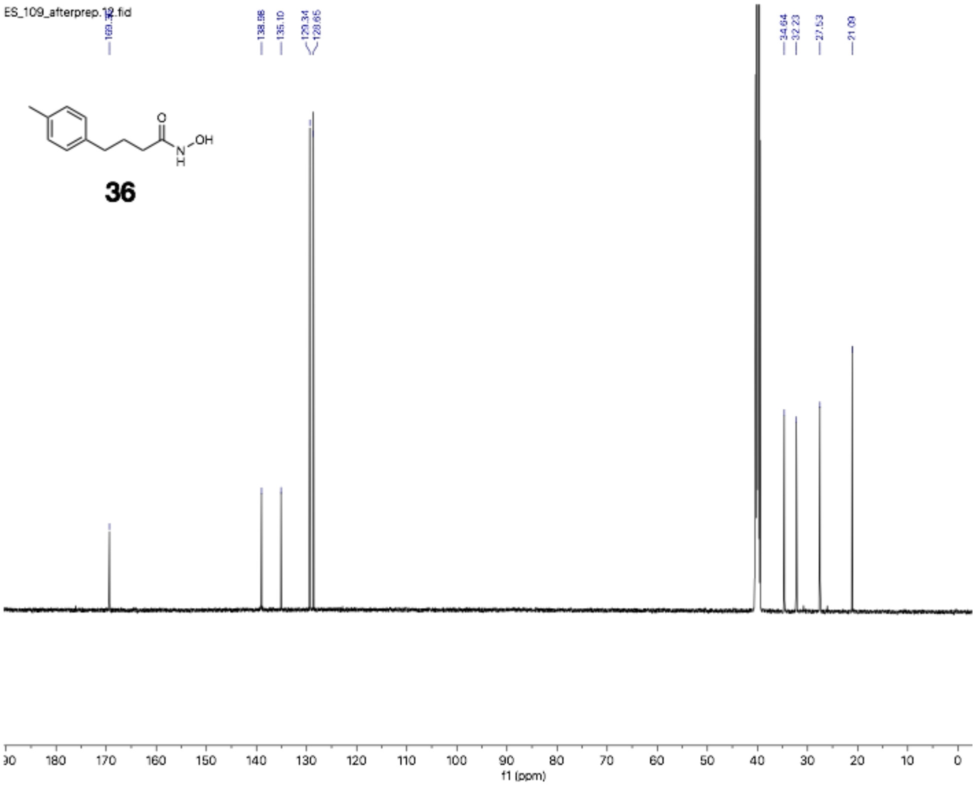
ES_109_afterprep.12.fid
36
138.98
135.10
129.34
128.65
34.64
32.23
27.53
21.09
f1 (ppm)

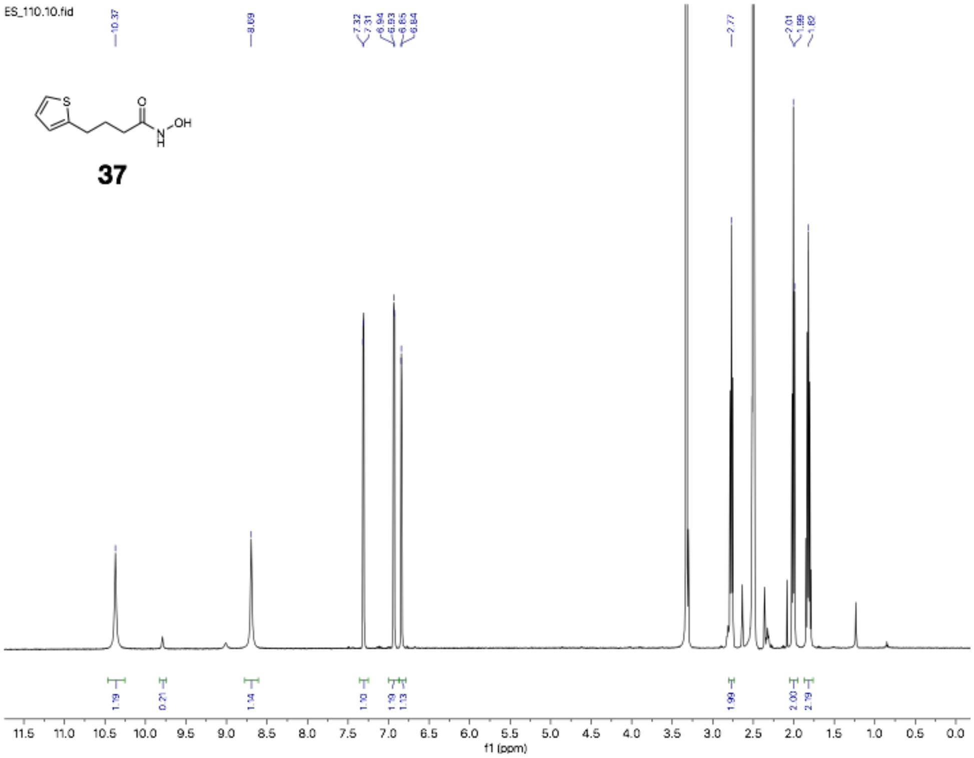
ES_110.10.fid
10.37
8.69
7.32
7.31
6.94
6.93
6.85
6.84
2.77
2.01
1.99
1.82
37
1.19
0.21
1.14
1.10
1.19
1.13
1.99
2.00
2.19
11.5 11.0 10.5 10.0 9.5 9.0 8.5 8.0 7.5 7.0 6.5 6.0 5.5 5.0 4.5 4.0 3.5 3.0 2.5 2.0 1.5 1.0 0.5 0.0
f1 (ppm)

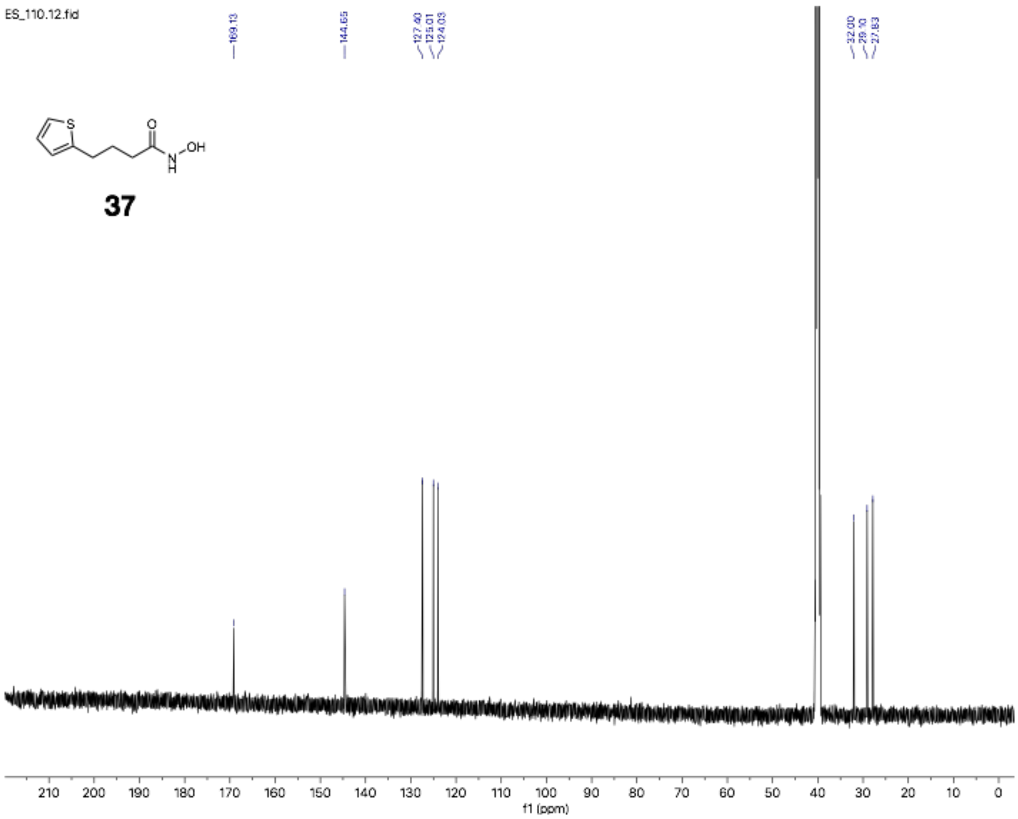
ES_110.12.fid
169.13
144.65
127.40
125.01
124.03
32.00
29.10
27.83
37
210 200 190 180 170 160 150 140 130 120 110 100 90 80 70 60 50 40 30 20 10 0
f1 (ppm)

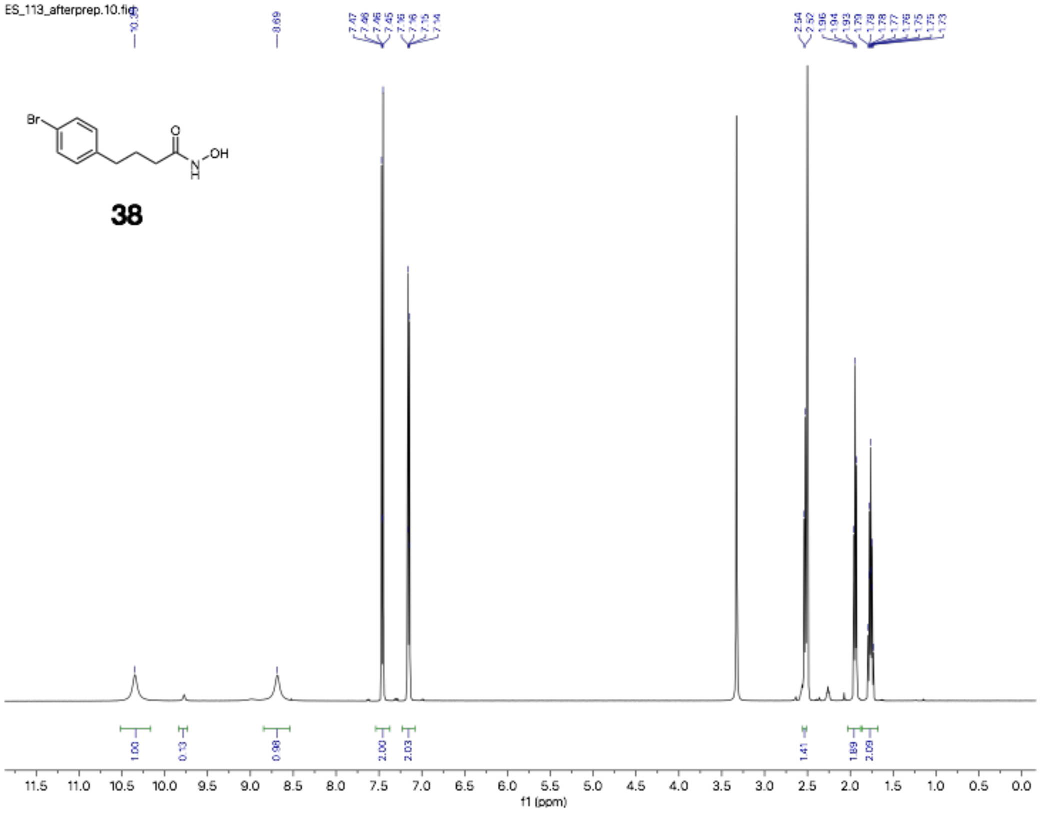
ES_113_afterprep.10.fid
38
f1 (ppm)

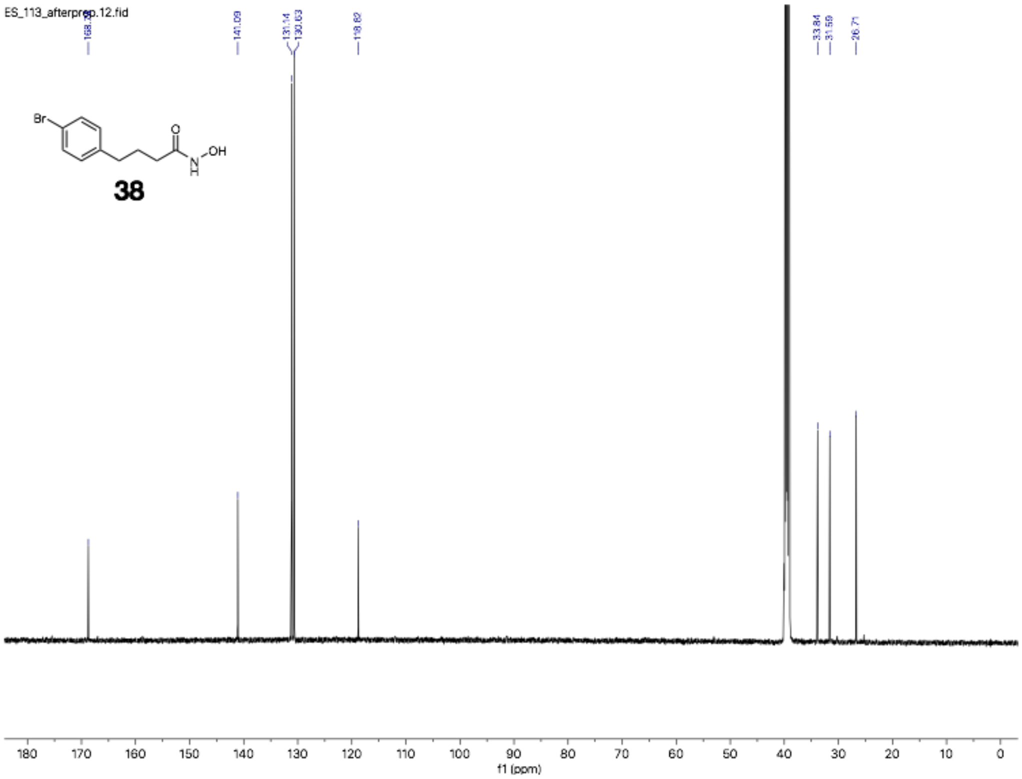
ES_113_afterprep.12.fid
38
f1 (ppm)

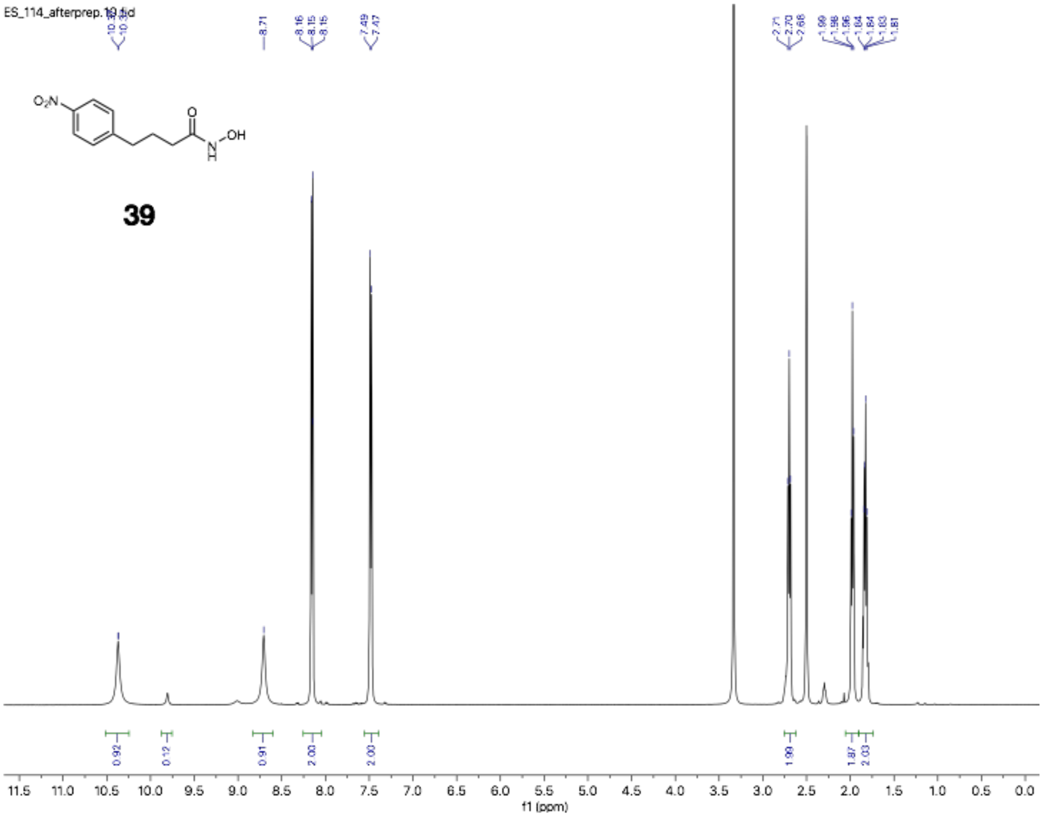
ES_114_afterprep.10.fid
39
f1 (ppm)

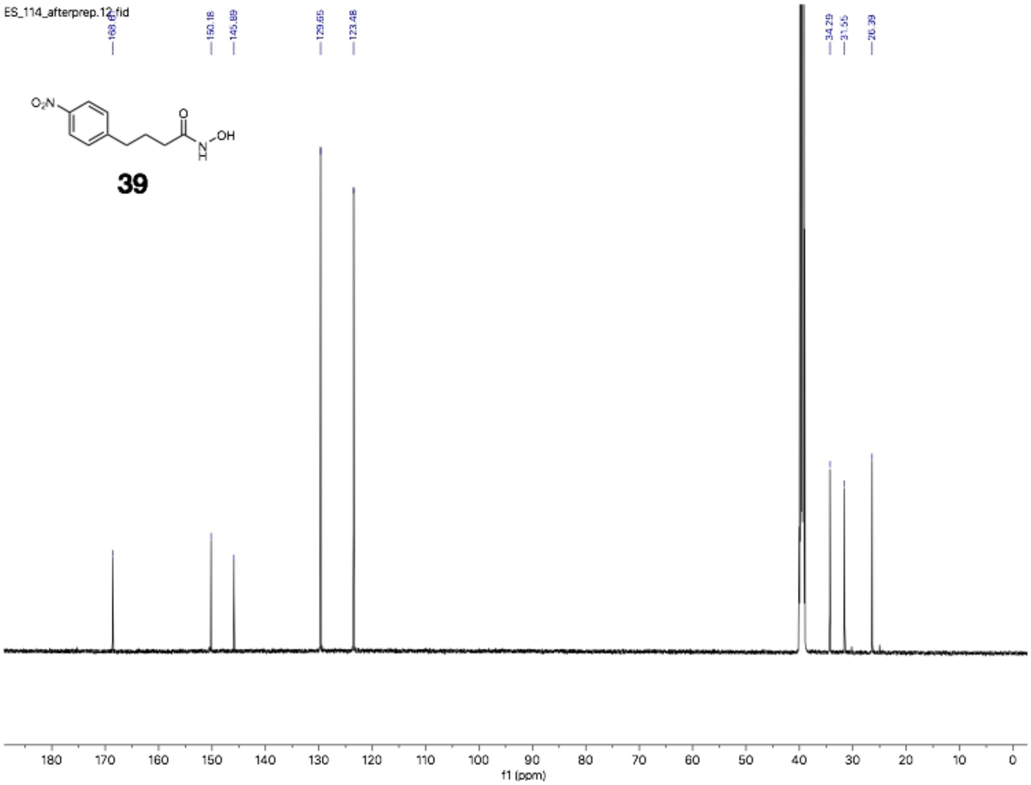
ES_114_afterprep.12.fid
39
f1 (ppm)

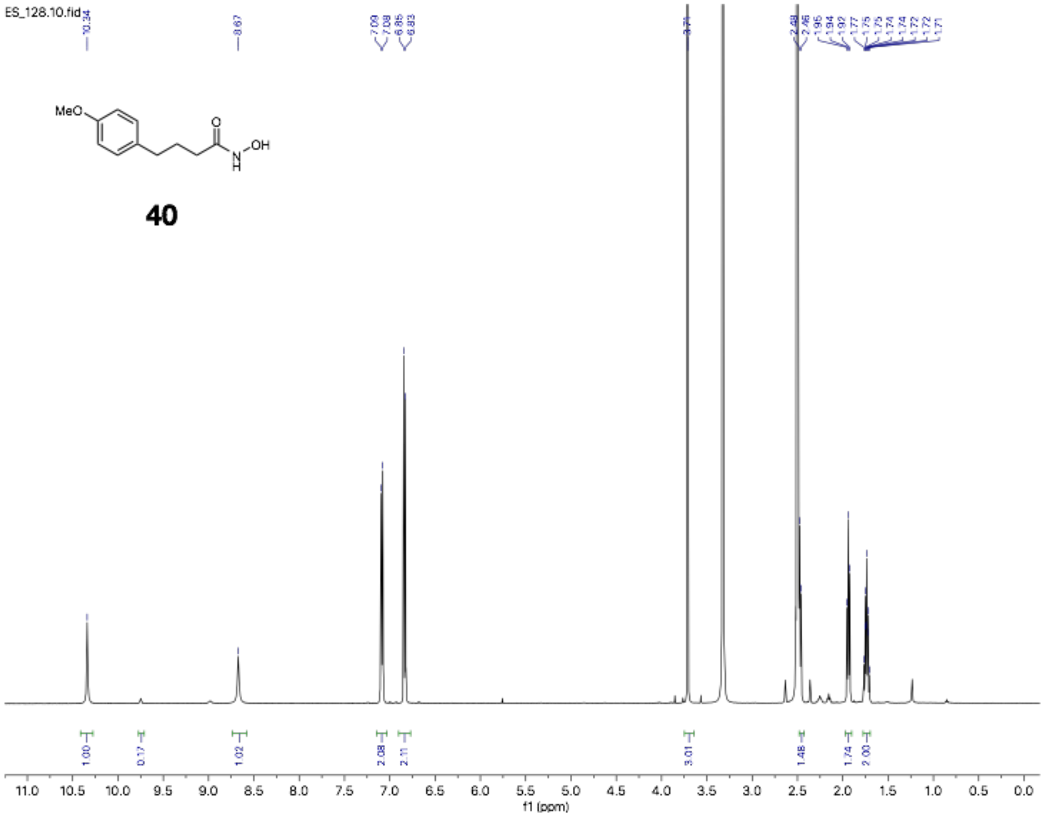

40

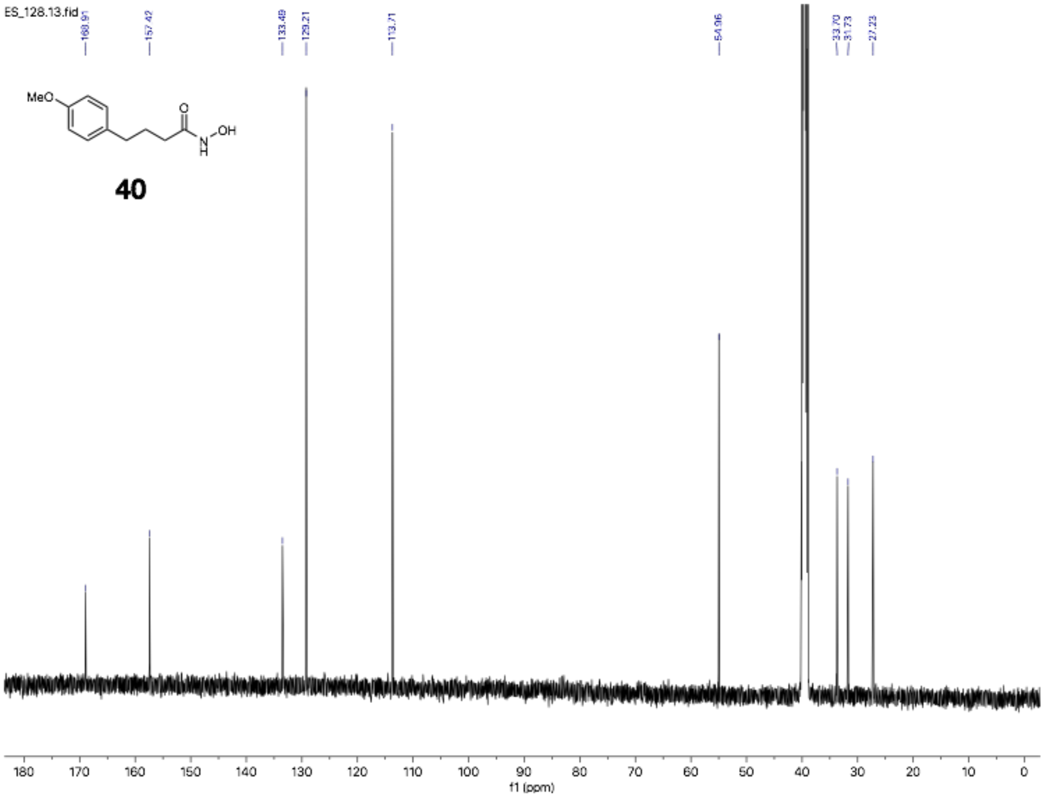

40

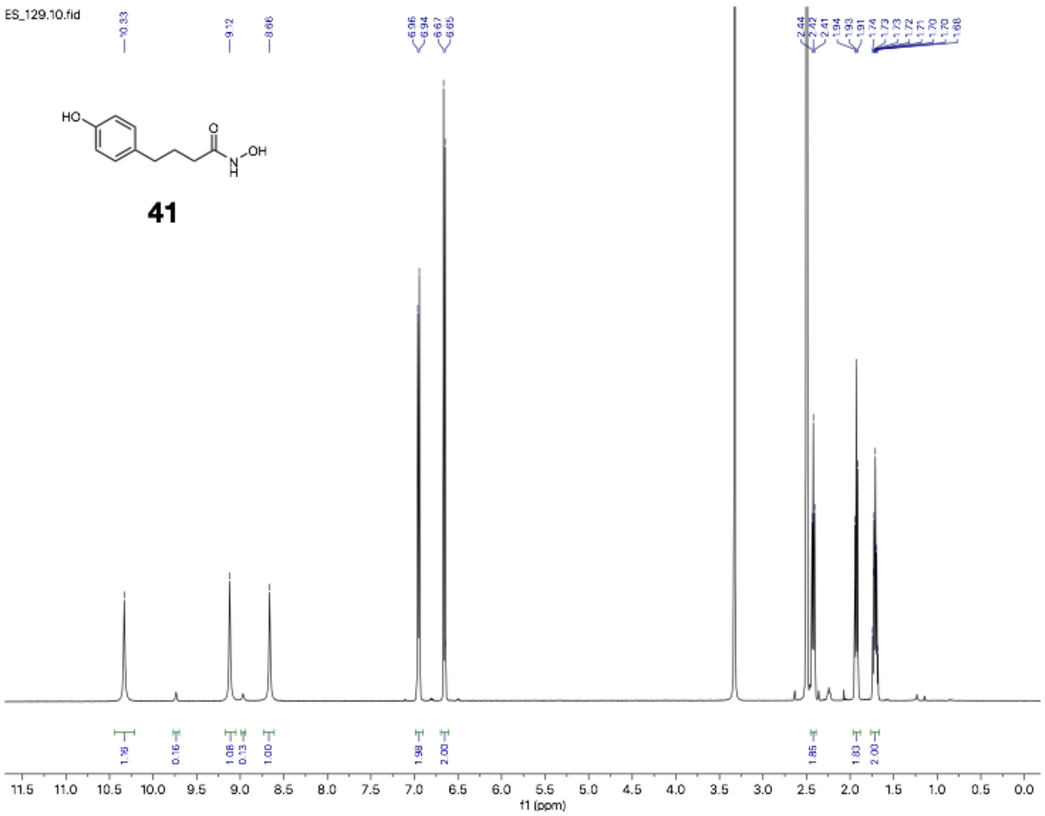
ES_129.10.fid
10.33
9.12
8.66
6.96
6.94
6.67
6.65
2.44
2.42
2.41
1.94
1.93
1.91
1.74
1.73
1.73
1.72
1.71
1.70
1.70
1.68
HO
OH
N
H
O
41
1.16
0.16
1.08
0.13
1.00
1.98
2.00
1.85
1.83
2.00
f1 (ppm)

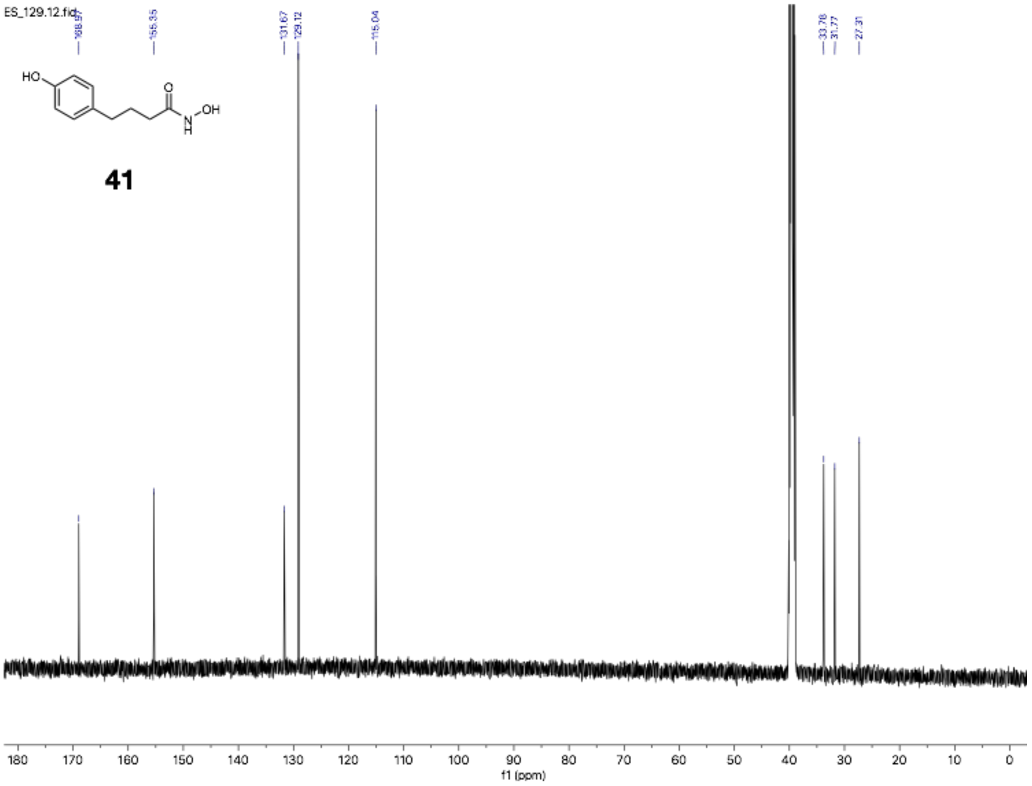
ES_129.12.fid
168.97
155.35
131.67
129.12
115.04
33.78
31.77
27.31
HO
OH
N
H
O
41
f1 (ppm)

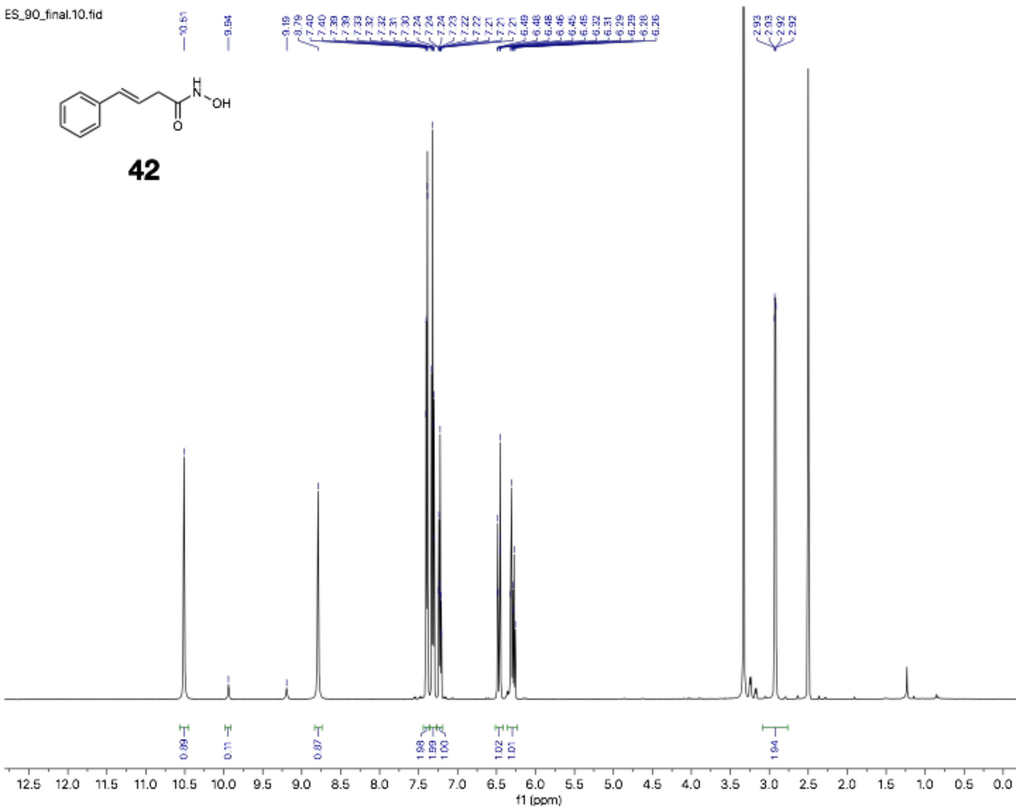
ES_90_final.10.fid
42
f1 (ppm)

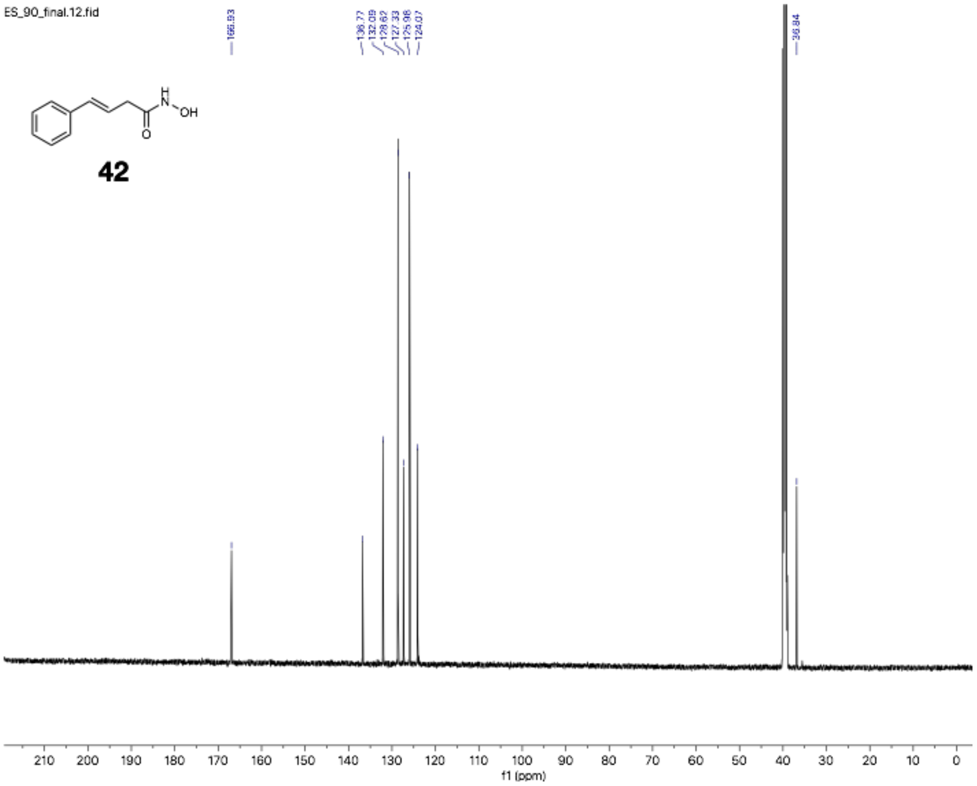
ES_90_final.12.fid
42
f1 (ppm)

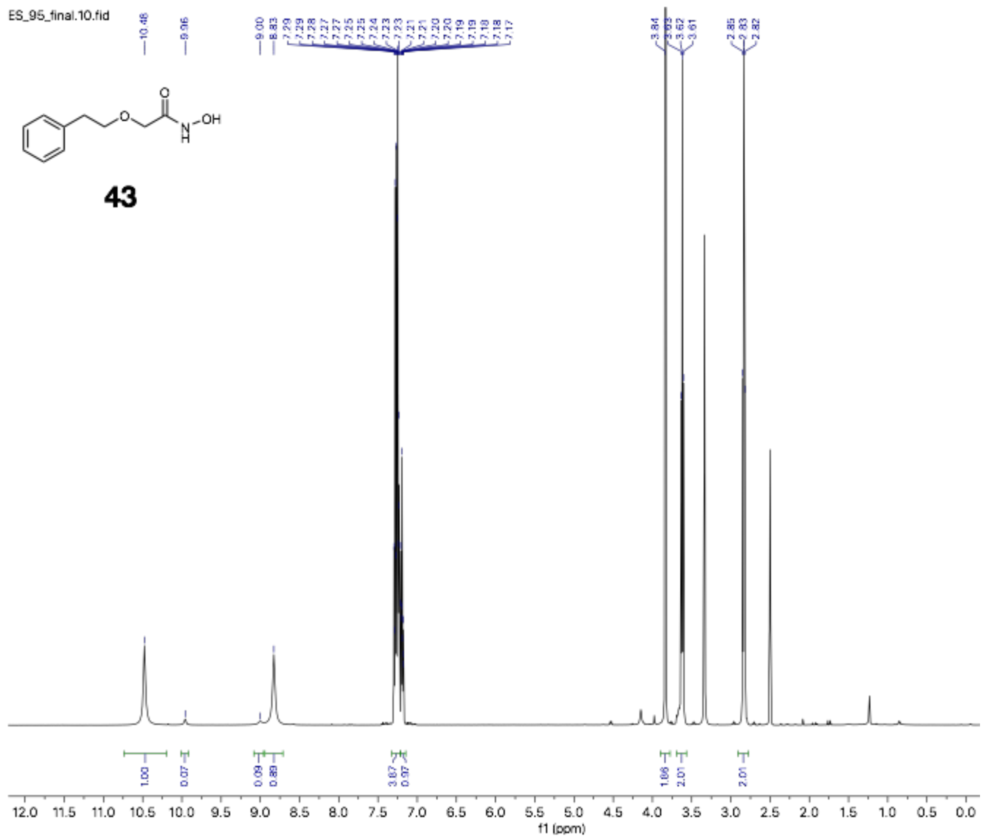

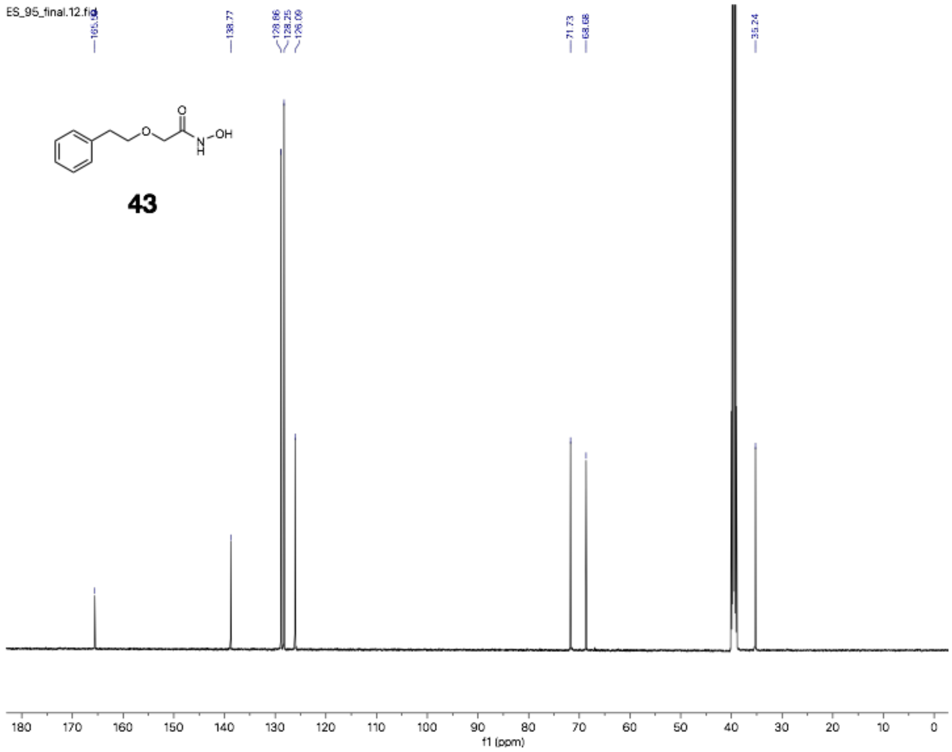

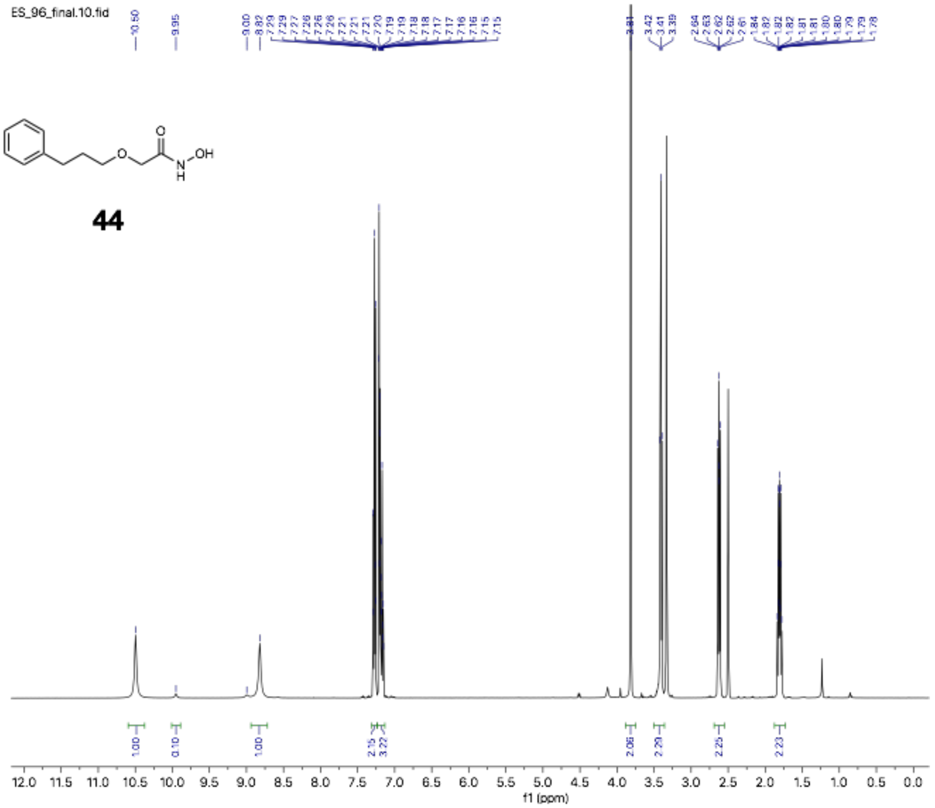
ES_96_final.10.fid
44
f1 (ppm)
10.50
9.95
9.00
8.82
3.81
3.42
3.41
3.39
1.00
0.10
1.00
2.15
3.22
2.06
2.29
2.29
2.23
12.0 11.5 11.0 10.5 10.0 9.5 9.0 8.5 8.0 7.5 7.0 6.5 6.0 5.5 5.0 4.5 4.0 3.5 3.0 2.5 2.0 1.5 1.0 0.5 0.0

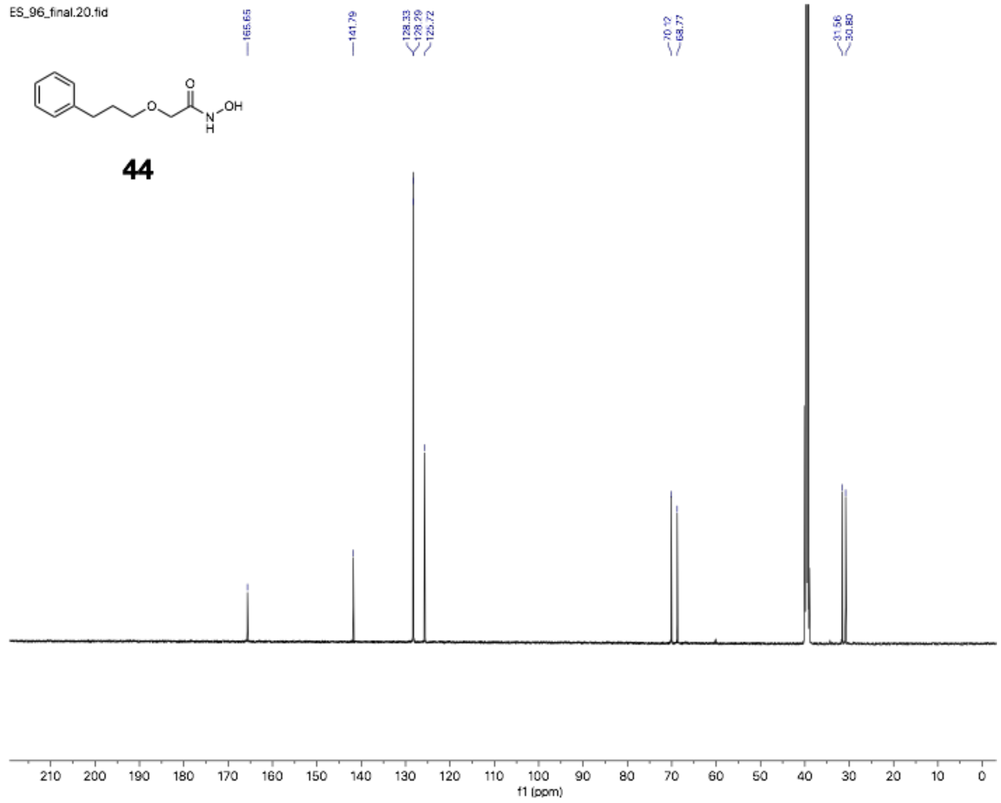
ES_96_final.20.fid
44
f1 (ppm)
165.65
141.79
128.33
128.29
125.72
70.12
68.77
31.56
30.80
210 200 190 180 170 160 150 140 130 120 110 100 90 80 70 60 50 40 30 20 10 0

References

1. Desaphy, J.; Raimbaud, E.; Ducrot, P.; Rognan, D., Encoding protein-ligand interaction patterns in fingerprints and graphs. *Journal of chemical information and modeling* **2013,** *53* (3), 623-37.
2. Troelsen, N. S.; Shanina, E.; Gonzalez-Romero, D.; Danková, D.; Jensen, I. S. A.; Śniady, K. J.; Nami, F.; Zhang, H.; Rademacher, C.; Cuenda, A.; Gotfredsen, C. H.; Clausen, M. H., The 3F Library: Fluorinated Fsp3-Rich Fragments for Expeditious 19F NMR Based Screening. *Angewandte Chemie International Edition* **2020,** *59* (6), 2204-2210.
3. Hajduk, P. J.; Huth, J. R.; Fesik, S. W., Druggability indices for protein targets derived from NMR-based screening data. *J Med Chem* **2005,** *48* (7), 2518-25.
4. Aretz, J.; Wamhoff, E.-C.; Hanske, J.; Heymann, D.; Rademacher, C., Computational and Experimental Prediction of Human C-Type Lectin Receptor Druggability. *Frontiers in Immunology* **2014,** *5* (323).
5. Shanina, E.; Kuhaudomlarp, S.; Lal, K.; Seeberger, P. H.; Imberty, A.; Rademacher, C., Druggable Allosteric Sites in β-Propeller Lectins. *Angewandte Chemie International Edition* **2022,** *61* (1), e202109339.
6. Harner, M. J.; Frank, A. O.; Fesik, S. W., Fragment-based drug discovery using NMR spectroscopy. *J Biomol NMR* **2013,** *56* (2), 65-75.
7. Chen, A. Y.; Adamek, R. N.; Dick, B. L.; Credille, C. V.; Morrison, C. N.; Cohen, S. M., Targeting Metalloenzymes for Therapeutic Intervention. *Chemical reviews* **2019,** *119* (2), 1323-1455.
8. Davis, B. J.; Erlanson, D. A., Learning from our mistakes: the 'unknown knowns' in fragment screening. *Bioorg Med Chem Lett* **2013,** *23* (10), 2844-52.
9. Hermann, J. C.; Chen, Y.; Wartchow, C.; Menke, J.; Gao, L.; Gleason, S. K.; Haynes, N.-E.; Scott, N.; Petersen, A.; Gabriel, S.; Vu, B.; George, K. M.; Narayanan, A.; Li, S. H.; Qian, H.; Beatini, N.; Niu, L.; Gan, Q.-F. Metal impurities cause false positives in high-throughput screening campaigns *ACS Med Chem Lett* [Online], 2013, p. 197-200. PubMed. http://europepmc.org/abstract/MED/24900642
https://www.ncbi.nlm.nih.gov/pmc/articles/pmid/24900642/pdf/?tool=EBI
https://www.ncbi.nlm.nih.gov/pmc/articles/pmid/24900642/?tool=EBI
https://doi.org/10.1021/ml3003296
https://europepmc.org/articles/PMC4027514
https://europepmc.org/articles/PMC4027514?pdf=render (accessed 2013/02//).
10. Advanced Chemistry Development (ACD/Labs) Software V11.02 (© 1994-2021 ACD/Labs), **2005**.
11. Aretz, J.; Baukmann, H.; Shanina, E.; Hanske, J.; Wawrzinek, R.; Zapol'skii, V. A.; Seeberger, P. H.; Kaufmann, D. E.; Rademacher, C., Identification of Multiple Druggable Secondary Sites by Fragment Screening against DC-SIGN. *Angewandte Chemie International Edition* **2017,** *56* (25), 7292-7296.
12. Aretz, J.; Anumala, U. R.; Fuchsberger, F. F.; Molavi, N.; Ziebart, N.; Zhang, H.; Nazaré, M.; Rademacher, C., Allosteric Inhibition of a Mammalian Lectin. *Journal of the American Chemical Society* **2018,** *140* (44), 14915-14925.

13. Bietz, S.; Urbaczek, S.; Schulz, B.; Rarey, M., Protoss: a holistic approach to predict tautomers and protonation states in protein-ligand complexes. *Journal of Cheminformatics* **2014,** *6* (1), 12.
14. Jain, A. N., Surflex: fully automatic flexible molecular docking using a molecular similarity-based search engine. *J Med Chem* **2003,** *46* (4), 499-511.
15. Hassan, M.; Brown, R. D.; Varma-O'brien, S.; Rogers, D., Cheminformatics analysis and learning in a data pipelining environment. *Molecular diversity* **2006,** *10* (3), 283-99.
16. Hawkins, P. C. D.; Skillman, A. G.; Nicholls, A., Comparison of Shape-Matching and Docking as Virtual Screening Tools. *J Med Chem* **2007,** *50* (1), 74-82.
17. Kabsch, W., XDS. *Acta crystallographica. Section D, Biological crystallography* **2010,** *66* (Pt 2), 125-32.
18. Evans, P. R., An introduction to data reduction: space-group determination, scaling and intensity statistics. *Acta crystallographica. Section D, Biological crystallography* **2011,** *67* (Pt 4), 282-92.
19. McCoy, A. J., Solving structures of protein complexes by molecular replacement with Phaser. *Acta crystallographica. Section D, Biological crystallography* **2007,** *63* (Pt 1), 32-41.
20. Emsley, P.; Lohkamp, B.; Scott, W. G.; Cowtan, K., Features and development of Coot. *Acta crystallographica. Section D, Biological crystallography* **2010,** *66* (Pt 4), 486-501.
21. Murshudov, G. N.; Skubak, P.; Lebedev, A. A.; Pannu, N. S.; Steiner, R. A.; Nicholls, R. A.; Winn, M. D.; Long, F.; Vagin, A. A., REFMAC5 for the refinement of macromolecular crystal structures. *Acta Crystallographica Section D* **2011,** *67* (4), 355-367.
22. Long, F.; Nicholls, R. A.; Emsley, P.; Graǽulis, S.; Merkys, A.; Vaitkus, A.; Murshudov, G. N., AceDRG: a stereochemical description generator for ligands. *Acta Crystallogr D Struct Biol* **2017,** *73* (Pt 2), 112-122.
23. Potterton, L.; Agirre, J.; Ballard, C.; Cowtan, K.; Dodson, E.; Evans, P. R.; Jenkins, H. T.; Keegan, R.; Krissinel, E.; Stevenson, K.; Lebedev, A.; McNicholas, S. J.; Nicholls, R. A.; Noble, M.; Pannu, N. S.; Roth, C.; Sheldrick, G.; Skubak, P.; Turkenburg, J.; Uski, V.; von Delft, F.; Waterman, D.; Wilson, K.; Winn, M.; Wojdyr, M., CCP4i2: the new graphical user interface to the CCP4 program suite. *Acta Crystallogr D Struct Biol* **2018,** *74* (Pt 2), 68-84.
24. Chen, V. B.; Arendall, W. B., 3rd; Headd, J. J.; Keedy, D. A.; Immormino, R. M.; Kapral, G. J.; Murray, L. W.; Richardson, J. S.; Richardson, D. C., MolProbity: all-atom structure validation for macromolecular crystallography. *Acta crystallographica. Section D, Biological crystallography* **2010,** *66* (Pt 1), 12-21.
25. McNicholas, S.; Potterton, E.; Wilson, K. S.; Noble, M. E., Presenting your structures: the CCP4mg molecular-graphics software. *Acta crystallographica. Section D, Biological crystallography* **2011,** *67* (Pt 4), 386-94.
26. Fulmer, G. R.; Miller, A. J. M.; Sherden, N. H.; Gottlieb, H. E.; Nudelman, A.; Stoltz, B. M.; Bercaw, J. E.; Goldberg, K. I., NMR Chemical Shifts of Trace Impurities:

Common Laboratory Solvents, Organics, and Gases in Deuterated Solvents Relevant to the Organometallic Chemist. *Organometallics* **2010,** *29* (9), 2176-2179.
27. Xiao, Z.-P.; Peng, Z.-Y.; Dong, J.-J.; Deng, R.-C.; Wang, X.-D.; Ouyang, H.; Yang, P.; He, J.; Wang, Y.-F.; Zhu, M.; Peng, X.-C.; Peng, W.-X.; Zhu, H.-L., Synthesis, molecular docking and kinetic properties of β-hydroxy-β-phenylpropionyl-hydroxamic acids as Helicobacter pylori urease inhibitors. *Eur J Med Chem* **2013,** *68*, 212-221.
28. Ohtsuka, N.; Okuno, M.; Hoshino, Y.; Honda, K., A base-mediated self-propagative Lossen rearrangement of hydroxamic acids for the efficient and facile synthesis of aromatic and aliphatic primary amines. *Organic & Biomolecular Chemistry* **2016,** *14* (38), 9046-9054.
29. Hermant, P.; Bosc, D.; Piveteau, C.; Gealageas, R.; Lam, B.; Ronco, C.; Roignant, M.; Tolojanahary, H.; Jean, L.; Renard, P. Y.; Lemdani, M.; Bourotte, M.; Herledan, A.; Bedart, C.; Biela, A.; Leroux, F.; Deprez, B.; Deprez-Poulain, R., Controlling Plasma Stability of Hydroxamic Acids: A MedChem Toolbox. *J Med Chem* **2017,** *60* (21), 9067-9089.
30. Kawase, M.; Kitamura, T.; Kikugawa, Y., Electrophilic aromatic substitution with N-methoxy-N-acylnitrenium ions generated from N-chloro-N-methoxy amides: syntheses of nitrogen heterocyclic compounds bearing a N-methoxy amide group. *The Journal of Organic Chemistry* **1989,** *54* (14), 3394-3403.
31. Kukosha, T.; Trufilkina, N.; Belyakov, S.; Katkevics, M., Copper-Catalyzed Cross-Coupling of O-Alkyl Hydroxamates with Aryl Iodides. *Synthesis* **2012,** *44* (15), 2413-2423.
32. Clark, A. J.; Al-Faiyz, Y. S. S.; Broadhurst, M. J.; Patel, D.; Peacock, J. L., Base catalysed rearrangement of N-alkyl-O-acyl hydroxamic acids: synthesis of 2-acyloxyamides. *Journal of the Chemical Society, Perkin Transactions 1* **2000,** (7), 1117-1127.
33. Trabulsi, H.; Guillot, R.; Rousseau, G., Preparation of Imino Lactones by Electrophilic Cyclization of β,γ-Unsaturated Hydroxamates: Formation of 3-Cyanoprop-2-en-1-ones through Fragmentation Reactions. *European Journal of Organic Chemistry* **2010,** *2010* (30), 5884-5896.
34. A. Publication, O. S., **1969**, 49, 50.
