# Supplementary material for: Targeting undruggable carbohydrate recognition sites through focused fragment library design
Source: Commun Chem. 2022 May 20;5:64. doi: 10.1038/s42004-022-00679-3 (PMC9814205; doi:10.1038/s42004-022-00679-3)
Supplement: Supplementary file 2 — Reporting Summary [file 42004_2022_679_MOESM2_ESM.pdf]

nature portfolio

Corresponding author(s): Prof. Dr. Christoph Rademacher

Last updated by author(s): Mar 10, 2022

Reporting Summary

Nature Portfolio wishes to improve the reproducibility of the work that we publish. This form provides structure for consistency and transparency in reporting. For further information on Nature Portfolio policies, see our Editorial Policies and the Editorial Policy Checklist.

Statistics

For all statistical analyses, confirm that the following items are present in the figure legend, table legend, main text, or Methods section.

n/a	Confirmed	
☐	☒	The exact sample size (*n*) for each experimental group/condition, given as a discrete number and unit of measurement
☐	☒	A statement on whether measurements were taken from distinct samples or whether the same sample was measured repeatedly
☒	☐	The statistical test(s) used AND whether they are one- or two-sided<br>*Only common tests should be described solely by name; describe more complex techniques in the Methods section.*
☒	☐	A description of all covariates tested
☒	☐	A description of any assumptions or corrections, such as tests of normality and adjustment for multiple comparisons
☐	☒	A full description of the statistical parameters including central tendency (e.g. means) or other basic estimates (e.g. regression coefficient) AND variation (e.g. standard deviation) or associated estimates of uncertainty (e.g. confidence intervals)
☒	☐	For null hypothesis testing, the test statistic (e.g. *F*, *t*, *r*) with confidence intervals, effect sizes, degrees of freedom and *P* value noted<br>*Give P values as exact values whenever suitable.*
☒	☐	For Bayesian analysis, information on the choice of priors and Markov chain Monte Carlo settings
☒	☐	For hierarchical and complex designs, identification of the appropriate level for tests and full reporting of outcomes
☒	☐	Estimates of effect sizes (e.g. Cohen's *d*, Pearson's *r*), indicating how they were calculated

Our web collection on statistics for biologists contains articles on many of the points above.

Software and code

Policy information about availability of computer code

Data collection	X-ray diffraction data were collected at SOLEIL-PROXIMA2 (Saint Aubin, France) using EIGER X 9M (Dectris) detector.
Data analysis	The recorded crystallographic data were indexed, integrated, and scaled using XDS and merged using AIMLESS. The structures were solved by molecular replacement using 1OKO as a searching template in PHASER, followed by further iterations of manual rebuilding in COOT and restrained refinement in REFMAC5. Hydroxamic acid ligand was manually built in ACEDRG22 in CCP4i2 suite. The final model was validated with MOLPROBITY, PDB-redo (https://PDB-redo.eu/) and wwPDB validation service (http://validate-rcsb-1.wwPDB.org/) prior to submission to the Protein Data Bank. All structural figures were prepared using CCP4MG. Data processing, refinement statistics and PDB ID of the deposited structure are provided in the SI (Table S5).

For manuscripts utilizing custom algorithms or software that are central to the research but not yet described in published literature, software must be made available to editors and reviewers. We strongly encourage code deposition in a community repository (e.g. GitHub). See the Nature Portfolio guidelines for submitting code & software for further information.

Data

Policy information about availability of data

All manuscripts must include a data availability statement. This statement should provide the following information, where applicable:

- Accession codes, unique identifiers, or web links for publicly available datasets
- A description of any restrictions on data availability
- For clinical datasets or third party data, please ensure that the statement adheres to our policy

Relevant data are available from the corresponding author on reasonable request. Atomic coordinates for LecA bound to hydroxamic acid 35 crystal structure has been

deposited in the Protein Data Bank under accession numbers 7FJH.

Field-specific reporting

Please select the one below that is the best fit for your research. If you are not sure, read the appropriate sections before making your selection.

☒ Life sciences ☐ Behavioural & social sciences ☐ Ecological, evolutionary & environmental sciences

For a reference copy of the document with all sections, see nature.com/documents/nr-reporting-summary-flat.pdf

Life sciences study design

All studies must disclose on these points even when the disclosure is negative.

Sample size	PrOF and HSQC / TROSY NMR: two-three independent experiments. The Kd values derived in PrOF NMR were two-three independent experiments. Competition 19F NMR experiments: two-three independent experiments. CellFy: two independent experiments (duplicates). FP assay were three independent experiments.
Data exclusions	No data was excluded.
Replication	SPR experiments were not reproduced due to the inconsistency with PrOF and FP assay data. Other replication experiments were successful.
Randomization	not applied
Blinding	not applied

Reporting for specific materials, systems and methods

We require information from authors about some types of materials, experimental systems and methods used in many studies. Here, indicate whether each material, system or method listed is relevant to your study. If you are not sure if a list item applies to your research, read the appropriate section before selecting a response.

Materials & experimental systems

n/a	Involved in the study	
☒	☐	Antibodies
☐	☒	Eukaryotic cell lines
☒	☐	Palaeontology and archaeology
☒	☐	Animals and other organisms
☒	☐	Human research participants
☒	☐	Clinical data
☒	☐	Dual use research of concern

Methods

n/a	Involved in the study	
☒	☐	ChIP-seq
☐	☒	Flow cytometry
☒	☐	MRI-based neuroimaging

Eukaryotic cell lines

Policy information about cell lines

Cell line source(s)	Langerin+ and DC-SIGN+ Raji cells: generated in our laboratory
Authentication	Langerin+ and DC-SIGN+ Raji cells were confirmed in flow cytometry to over-express Langerin or DC-SIGN.
Mycoplasma contamination	Raji cells were tested regularly for mycoplasma contamination in our laboratory.
Commonly misidentified lines (See ICLAC register)	Raji cell, suitable for the binding studies

Flow Cytometry

Plots

Confirm that:

- [x] The axis labels state the marker and fluorochrome used (e.g. CD4-FITC).
- [x] The axis scales are clearly visible. Include numbers along axes only for bottom left plot of group (a 'group' is an analysis of identical markers).
- [ ] All plots are contour plots with outliers or pseudocolor plots.
- [ ] A numerical value for number of cells or percentage (with statistics) is provided.

Methodology

Sample preparation	The cellFy experiments using Langerin+ and DC-SIGN+ Raji cell lines were performed as described before.54 Briefly, 50k cells were plated in a 96-well plate (clear, round bottom; Greiner Bio-One and mixed with varying concentrations of malonates 58, D-mannose and 0.025 mg mL-1 FITC-conjugated dextran (500 kDa, Sigma Aldrich) in a final volume of 50 µL following incubation for 30 min on ice. After centrifugation at 500 g for 3 min at 4 °C, supernatant was discarded. After washing cells were treated with 50 µL 4 % paraformaldehyde (Roti-Histofix, Carl Roth) for 20 min on ice and resuspended in 100 µL fresh culture medium.
Instrument	MACSQuant Analyzer 16
Software	FlowJo
Cell population abundance	Cell line: one population for both cell-lines Langerin+ and DC-SIGN+ Raji cell lines
Gating strategy	Doublets and dead cells were excluded, FITC channel was observed

- [ ] Tick this box to confirm that a figure exemplifying the gating strategy is provided in the Supplementary Information.
